# Assessment tools addressing avoidable care transitions in older adults: a systematic literature review

**DOI:** 10.1007/s41999-024-01106-7

**Published:** 2024-11-29

**Authors:** Rustem Makhmutov, Alicia Calle Egusquiza, Cristina Roqueta Guillen, Eva-Maria Amor Fernandez, Gabriele Meyer, Moriah E. Ellen, Steffen Fleischer, Anna Renom Guiteras

**Affiliations:** 1 Medical Faculty, Institute for Health and Nursing Science, Martin Luther University Halle-Wittenberg, Magdeburger Straße 8, 06112 Halle (Saale), Germany; 2 Geriatrics Department, Hospital del Mar, Llull 410, 08019 Barcelona, Spain; 3 Department of Health Policy and Management, Guilford Glazer Faculty of Business and Management and Faculty of Health Sciences, Ben-Gurion University of the Negev, David Ben Gurion Blvd 1, POB 653, 84105 Beer-Sheva, Israel; 4 Institute of Health Policy Management and Evaluation, Dalla Lana School of Public Health, University of Toronto, Toronto, Canada; 5 Universitat Pompeu Fabra (UPF), 08003 Barcelona, Spain

**Keywords:** Decision-making, Preventable care transitions, Decision support tools, Older adults

## Abstract

**Purpose:**

The phenomenon of avoidable care transitions has received increasing attention over the last decades due to its frequency and associated burden for the patients and the healthcare system. A number of assessment tools to identify avoidable transitions have been designed and implemented. The selection of the most appropriate tool appears to be challenging and time-consuming. This systematic review aimed to identify and comprehensively describe the assessment tools that can support stakeholders´ care transition decisions on older adults.

**Methods:**

This study was conducted as part of the TRANS-SENIOR research network. A systematic search was conducted in MEDLINE via PubMed, CINAHL, and CENTRAL. No restrictions regarding publication date and language were applied.

**Results:**

The search in three electronic databases revealed 1266 references and screening for eligibility resulted in 58 articles for inclusion. A total of 48 assessment tools were identified covering different concepts, judgement processes, and transition destinations. We found variation in the comprehensiveness of the tools with regard to dimensions used in the judgement process.

**Conclusion:**

All tools are not comprehensive with respect to the dimensions covered, as they address only one or a few perspectives. Although assessment tools can be useful in clinical practice, it is worth it to bear in mind that they are meant to support decision-making and supplement the care professional´s judgement, instead of replacing it. Our review might guide clinicians and researchers in choosing the right tool for identification of avoidable care transitions, and thus support informed decision-making.

**Supplementary Information:**

The online version contains supplementary material available at 10.1007/s41999-024-01106-7.

## Key summary points

**Aim:**

To identify and comprehensively describe the assessment tools addressing avoidable care transitions that can support stakeholders´ decisions on older adults.

**Findings:**

All of the 48 reviewed tools are not comprehensive with respect to the dimensions covered, making them less useful in addressing avoidable care transitions. The review findings are systematically summarised in a clinically accessible website (www.decision4transition.com), which allows to instantly filter assessment tools based on their properties.

**Message:**

The review findings and the online database are now ready for use in clinical routine to support informed decision-making of stakeholders when choosing the right assessment tool addressing avoidable care transitions.

**Supplementary Information:**

The online version contains supplementary material available at 10.1007/s41999-024-01106-7.

## Background

Older adults have an increased risk of frequent transitions across care settings. These transitions are often associated with negative outcomes for the person concerned, such as a decline in autonomy, reduced quality of life, more adverse medical events, and even increased mortality, as well as increased direct and opportunity costs for the healthcare system [1–3]. Care transitions have been defined as changes in the care provision setting [4, 5], encompassing care settings, such as hospitals, nursing homes, primary care, home care, and palliative care. Therefore, a care transition is an umbrella term that covers different types of transitions, such as (re)admissions and discharges. In addition, care transitions occur not only between care settings but also within care settings [6, 7], for example between wards and medical departments in the same hospital.

Some of these care transitions are regarded as avoidable. The phenomenon of avoidable care transitions has received increasing attention over the last decades due to its frequency and associated burden for the patients and the healthcare system [8–13].

Hospital (re)admission is one of the most common types of avoidable care transitions. International studies indicate a wide range of avoidable (re)admission rates from 5 to 79% [11, 13–17]. To a certain extent, the variation is likely due to different data collection methods, different populations among studies and countries, and differences in the definition of “avoidable” across settings and agencies [11, 13, 14, 16, 18].

It is of utmost importance to reduce the number of avoidable care transitions to minimize the burden on patients and healthcare systems. To achieve this, it is essential, first and foremost, to establish a clear definition of avoidable care transitions. Second, the identification of these avoidable care transitions becomes imperative. A recent study by Makhmutov et al. [19], addressed the challenge of a transparent definition and delivered a comprehensive consensus-based definition for “avoidable care transitions” endorsed by an international panel of experts. Existing tools are intended for specific settings and/or patients, implying that there is no “gold standard” tool that can be applied to all types of care transitions. Even within a specific type of care transition (e.g., care transitions from long-term care facilities to hospital), studies apply different assessment tools to identify avoidable care transitions [14].

Reviews that overviewed assessment tools or interventions dealing with avoidability of care transitions focused on specific types of care transitions [14, 20–23]. However, no systematic literature review has evaluated the scientific evidence on the existing assessment tools dealing with avoidable care transitions among older adults without any restriction to particular care settings. Such a systematic review would not only extend and update the previous reviews but also might guide researchers and clinicians in informed decision-making in choosing the right tool.

Thus, the aim of our systematic literature review is twofold: (1) to provide a comprehensive overview of assessment tools dealing with avoidable care transitions among older adults and (2) to provide a critical analysis of the identified assessment tools.

## Methods

### Search strategy and selection criteria

The review protocol has been registered under PROSPERO registration number CRD42022312516. A systematic search was conducted in MEDLINE via PubMed, CINAHL, and CENTRAL on June 23, 2022. No restrictions regarding publication date and language were applied. Since the review is embedded within the TRANS-SENIOR project on older adults and European long-term care systems, we included studies that examined older adults aged 60 years and above and that were conducted not only in European countries, but also in other Western countries. Western countries cover European countries (EU 27 countries and the UK, Iceland, Norway, Switzerland), North America (USA, Canada), Australia, and New Zealand.

We subdivided the articles into two categories and included studies which used (1) assessment tools (fully or partially) as an intervention to support decision-making on avoidability of care transitions compared to usual care (e.g., including alternative interventions or no interventions at all, depending on normal care standards), and (2) assessment tools as an instrument to determine risk for or incidence of avoidable care transitions. For the first category, RCTs and controlled trials were eligible for inclusion. For the second category, all study designs were considered, except for editorials, conference abstracts, commentaries, and opinion papers. Articles were excluded when they did not meet the inclusion criteria.

The search string was developed by one author (RM), reviewed by the co-authors (SF, GM, and ME), and tested multiple times until consensus on the final search string was reached by all of the authors. A medical librarian was not involved in designing the search strategy. The complete final search string is displayed in Supplementary file 7 (*Final search string*), and embraces search terms such as “tool”, “avoidable”, “transition”, and “older adults” and their related terms.

All search results from the three databases were imported to the EndNote citation manager software and checked for duplicates automatically and manually. Remaining studies were exported to the Covidence systematic review management software. A list of included and de-duplicated studies was screened twice in Covidence software, to identify eligible articles for both categories. Title and abstract, and full-text screening were performed independently by a pair of reviewers (RM and ARG/ACE/EMAF/CRG/SF). Forward citation tracking was conducted, and reference lists of included studies were screened to identify relevant references.

### Data analysis

#### Included studies

The systematic literature review followed the PRISMA reporting guidelines.

Two PRISMA flow diagrams display the screening process of studies included in category 1 and 2, followed by summary of the included studies.

Two different data extraction forms were developed and pilot-tested for the two categories. Two researchers (RM and ARG/ACE/EMAF/CRG) independently extracted data on the study characteristics and the assessment tools. In the case of disagreement, a third author was consulted to reach consensus.

Data extraction forms included I) information on study characteristics such as design, aims, population, and settings; II) details on identified assessment tools such as concepts covered by the assessment tools, target population, type of care transitions targeted, how an assessment tool is organised (i.e. table form, check-list or other), where used or tested; and III) details on study outcomes as reported.

We conducted a risk-of-bias assessment of included studies within category 1. Two reviewers from the research team independently reviewed each study using the revised Cochrane risk-of-bias tool for randomized trials (RoB 2) [24]. Results were compared, and in the case of disagreement, a third reviewer from the research team was consulted to reach consensus. We refrained from formal risk-of-bias assessment of studies within category 2. The research team considered that risk-of-bias assessment in this case would not have provided any substantial information.

#### Identified assessment tools

To fulfil aim 2 of this review, we conducted a critical analysis of the identified assessment tools addressing four major criteria that were inspired by Bühner [25], i.e., objectivity (process, evaluation, interpretation), reliability (inter- and intra-rater), validity (convergent), and costs (time for completion of a tool, specific input required for a tool, specific training required to use a tool). Supplementary file 1 displays the template for the analysis of the tools. This template was designed by two authors (RM, SF) and reviewed by the co-authors. This review did not aim to report only on the best-performing assessment tools, but rather to describe and analyse all identified assessment tools.

We clustered concepts covered by assessment tools into overarching so-called patient, clinical, social, and system level factors [26], because it would enhance comparison among assessment tools and unify findings. These factors are also reflected in four recent publications [17, 27–29], confirming relevancy and importance of these factors. Patient factors refer to socioeconomic status, health status, and behaviour of a person such as noncompliance with treatment or failure to seek medical attention when needed [13, 26]. Clinical factors cover appropriateness of assessment and treatment, such as adequacy of clinical management, appropriate discharge planning, or outpatient care following discharge [13, 26]. Social factors include three elements, namely, coping, carer system, and community service [13, 26]. System factors relate to the availability, accessibility, and coordination of care across the health care system, for example, provision of resources at home according to a person´s needs [13, 26].

Overall, assessment tools were summarised with respect to concepts covered, judgement process, focus of measurement, and usage by specific group of persons. A fictional vignette case was also developed to illustrate application of different tools in actual clinical practice. Furthermore, the tools identified from studies included as part of category 1 were summarised in more detail within the article, as these tools were reported in RCTs and controlled trials, thus allowing to draw compelling evidence on the tools. Representing our review findings through an interactive and practical format seemed to be the next logical step, hence, a new online interactive database summarising the review findings was developed and launched.

## Results

### Included studies

**Fig. 1:**
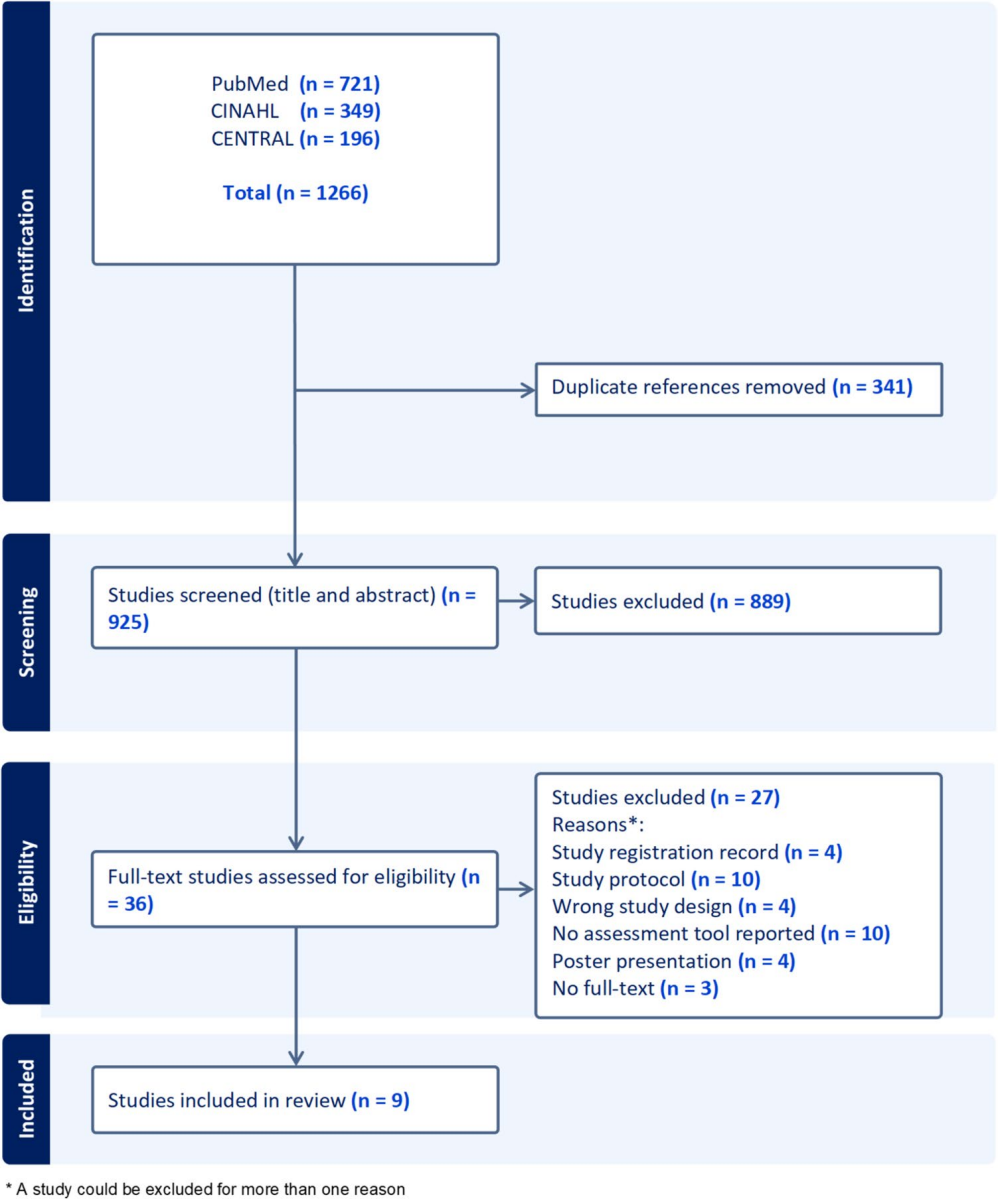
PRISMA flow diagram for studies screened as part of category 1

**Fig. 2:**
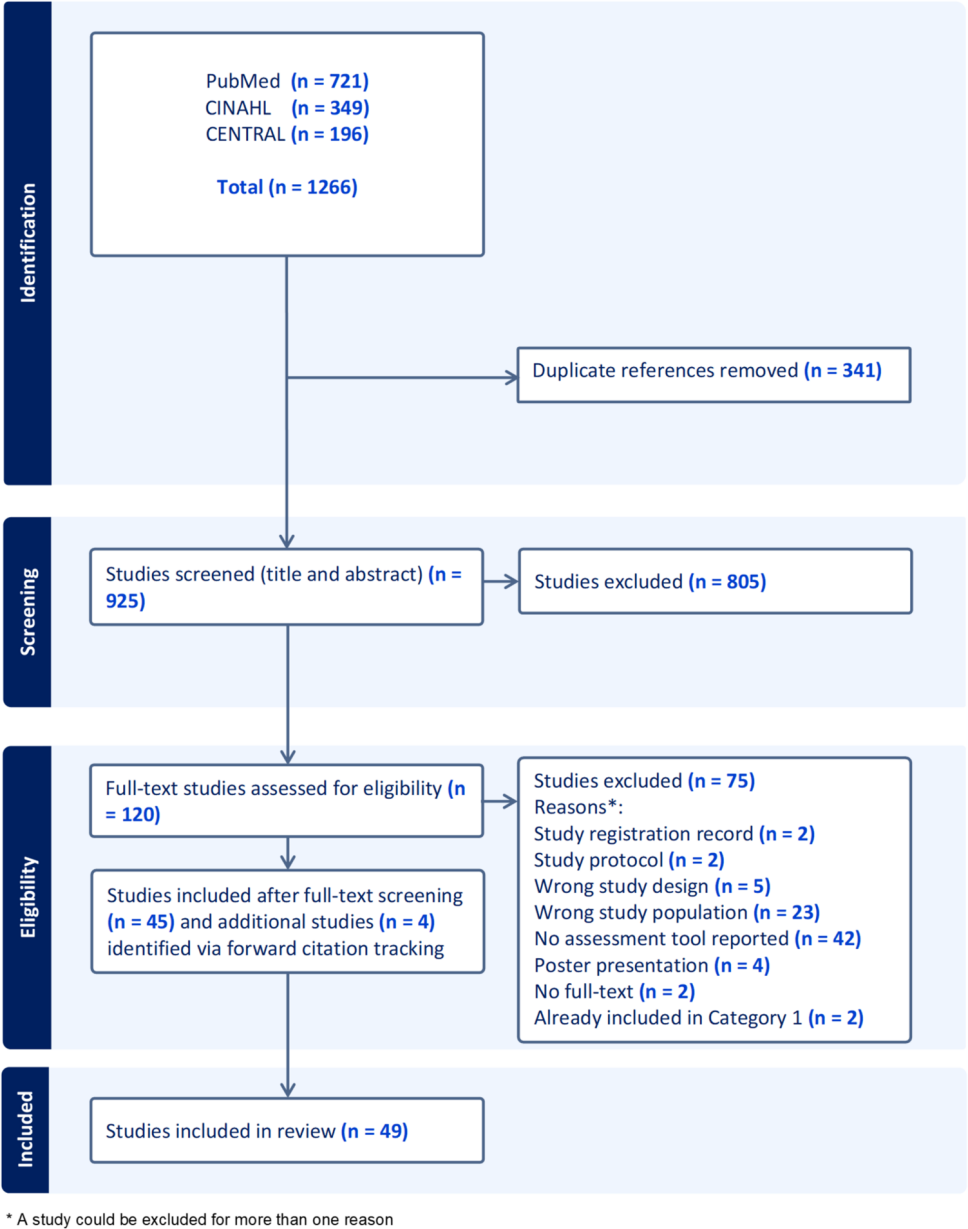
PRISMA flow diagram for studies screened as part of category 2

Fifty-eight articles were included, of which 9 belong to category 1 (*assessment tools as an intervention to support decision-making on avoidability of care transitions compared to usual care: RCTs and controlled trials*) [30–38] and 49 to category 2 (*assessment tools as an instrument to determine risk for or incidence of avoidable care transitions: all study design with some exceptions*) [39–87]. Figures 1 and 2 display the selection process of the studies.

Supplementary file 2 (*Characteristics of the included studies*) presents an overview of the characteristics of the included studies. Studies belonging to category 2 represented various study designs, such as routine data analyses, cohort studies, surveys, interviews, cross-sectional studies, and pre-post interventions. Most of the studies exclusively focussed on a particular assessment tool, while others reported on an intervention or a strategy where an assessment tool was part of it, such as INTERACT intervention [34, 37], Aged Care Emergency Service (ACE) service model [32, 33], and Better Health in Residents of Care Homes with Nursing (BHiRCH-NH) intervention [35].

Populations from several care settings were studied, including hospitals, nursing homes, emergency departments, and intensive care units. The majority of studies considered the general population, while some others focused on specific groups, for example, patients with community-acquired pneumonia (CAP) [40, 55], residents at the end-of-life [66], patients with polymedication [63], patients with acute heart failure [79], and patients with chronic obstructive pulmonary disease (COPD) [78]. Investigated acute-care destinations also varied considerably among studies, for instance, (re)hospitalisations [41, 43–45, 86], transfers from long-term care facilities or community to hospital or emergency department (ED) [56, 58, 60, 62, 73], transitions to intensive care units (ICU) [80], and transitions to or discharges from ED [70, 78, 79].

Rates of avoidable care transitions also varied substantially from 1.6% to 77% [47, 48, 69, 73].

The results of the risk-of-bias assessment of the included studies in category 1 are presented in the Supplementary file 4 (*RoB table*). Nine articles reported on six studies; therefore, critical appraisal has been done for six primary articles representing each study. Three articles [32, 33, 36] were assessed as showing a high risk of selection bias through lack of proper randomization and two [32, 34] articles indicated a high risk in the domain of missing outcome data. Overall, two articles [35, 38] were judged as having some concerns for bias, while the other four publications were rated as having a high risk for bias.

### Identified assessment tools

A total of 48 assessment tools dealing with avoidable care transitions were identified from the included studies. Results of the critical analysis are displayed in Supplementary file 3 (*Characteristics of the tools*). Those assessment tools without their own name were given the name of the first author of the corresponding study.

Some of the identified assessment tools were reported to perform poorly. For example, Johnston, Longman [60] concluded that the Preventability Assessment Tool (PAT) is not a valid tool for assessing preventability of unplanned hospital admissions.

As can be seen from Supplementary file 3 (*Characteristics of the tools*), the assessment tools differ widely with respect to concepts they cover. For example, Appropriateness Evaluation Protocol (AEP) and care pathway from the BHiRCH-NH intervention include a list of conditions or diseases (e.g., dehydration, congestive heart failure) [35, 46]; the tools by Bermejo Higuera, Gozalo, and Ong consist of pre-defined criteria in the form of statements [47, 56, 66]; the Walter indicator, rectal bleeding admission guide and algorithm, and the CURB-65 score include laboratory or clinical characteristics [59, 61, 70]; the Comprehensive Geriatric Assessment (CGA) includes physical/functional/social/economic and mental dimensions [85, 87]; patient´s care preferences, clinical care resources, and quality of acute care, among other things, are addressed by Structured Implicit Record review (SIR) [69, 73]. Overall, 48 tools cover patient factors, whereas 15 tools cover clinical factors, three social factors, and 15 system level factors. Ideally, a tool should include all four factors, which would make a tool fully comprehensive. The more factors a tool has, the more comprehensive it becomes in addressing avoidability of care transitions. However, none of the identified tools included all four factors. Table 1 provides an overview of identified assessment tools, with further information on factors covered by each tool.

**Table 1:** An overview of identified assessment tools

#	Tool name	Concepts grouped into patient, clinical, social, and system factors	Target population	Care transition
1	INTERACT tools (with focus on care paths only)	Patient factors	Nursing home residents	Nursing home → Hospital, Nursing home → Emergency department
2	QI review tool (from project INTERACT II)	Patient factors, Clinical factors	Nursing home residents	Nursing home → Hospital
3	INTERACT II tools (with focus on care paths only)	Patient factors	Nursing home residents	Nursing home → Hospital
4	Root cause analysis (INTERACT QI Acute care transfers (ACT) tool)	Patient factors, Clinical factors	Nursing home residents	Nursing home → Hospital
5	ACE model (with focus on evidence-based algorithms only)	Patient factors	Nursing home residents	Nursing home → Hospital, Nursing home → Emergency department
6	A complex intervention to reduce avoidable hospital admissions in nursing homes (with focus on a care pathway only)	Patient factors	Nursing home residents	→ Hospitalizations
7	Novel Decision Guide "Go to the Hospital or Stay Here?"	Patient factors, Social factors System factors	Nursing home residents, families, caregivers, and friends	Nursing home → Hospital
8	ACI-TIPI acute cardiac ischemia time-insensitive predictive instrument	Patient factors	ED patients with chest pain	→ Hospital and CCU (coronary care unit) admissions
9	Appropriateness Evaluation Protocol (AEP) (with focus on criteria of appropriateness of admission only)	Patient factors, Clinical factors, System factors	Adult patients with acute conditions (reliable for any type of diagnosis). Not suitable for paediatric, obstetric, or psychiatric patients	→ Hospitalizations
10	Appropriateness Evaluation Protocol French version (AEPf)	Patient factors, Clinical factors, System factors	Nursing home residents, hospitalized patients via emergency department, emergency department patients, and patients discharged from acute geriatric unit	→ Hospitalizations, → Rehospitalizations, Nursing home → Acute geriatric unit
11	Appropriateness Evaluation Protocol Geriatric adaptation (AEPg)	Patient factors, Clinical factors, System factors	Nursing home residents	Nursing home → Acute geriatric unit
12	Adapted AEP	Likely Patient factors, Clinical factors, System factors	Older adults	→ Hospitalizations
13	AEP Italian version	Likely Patient factors, Clinical factors, System factors	Medical patients	→ Hospitalizations
14	AEP Spanish version	Likely Patient factors, Clinical factors, System factors	Medical patients	→ Emergency department, Emergency → Hospital, Consultation → Hospital, Home → Hospital
15	Modified Italian AEP	Patient factors, Clinical factors System factors	Community-acquired pneumonia patients	→ Hospitalizations
16	CURB-65 score (in hospital setting)	Patient factors	Community-acquired pneumonia patients admitted to hospital	→ Hospitalizations
	CURB-65 score (for Community setting)	Patient factors	Community-acquired pneumonia patients in community	→ Hospitalizations
17	Risk Nomogram	Patient factors	Community patients discharged from the emergency department	Community → Emergency department readmissions within 28 days of emergency department discharge
18	HOSPITAL score	Patient factors, Clinical factors	Discharged acute and non-acute patients (medical and surgical wards), patients with polymedications	→ 30-day rehospitalizations
19	Simplified HOSPITAL score	Patient factors	Medical patients	→ 30-day rehospitalizations
20	LACE index	Patient factors	Medical and surgical patients discharged to the community	→ 30-day rehospitalizations
21	Revised LACE index	Patient factors	Older adults	→ 30-day rehospitalizations
22	New Zealand version of Patients at Risk of Hospital Readmission (PARR) predictive risk tool	Patient factors	Medical patients	→ 30-day rehospitalizations
23	PAR-Risk Score	Patient factors	Medical patients	Home → 30-day rehospitalizations
24	EOL care pathway	Patient factors, Clinical factors, Social factors	Nursing home residents	Nursing home → Hospital
25	The Identification of Seniors at Risk (ISAR) scale	Patient factors	Emergency department patients	Emergency department → Home or usual Nursing home, Emergency department → Acute hospital, Emergency department → Long term nursing care
26	The Silver Code	Patient factors	Emergency department patients	Emergency department → Home or usual Nursing home, Emergency department → Acute hospital, Emergency department → Long term nursing care, → Hospitalizations, → Emergency department readmissions
27	The Walter indicator	Patient factors	Hospital patients	Emergency department → Home or usual Nursing home, Emergency department → Acute hospital, Emergency department → Long term nursing care
28	Preventability Assessment Tool (PAT)	Patient factors, Clinical factors System factors	Community-dwelling patients with unplanned hospitalizations, with a primary discharge diagnosis of COPD, CHF, angina pectoris or diabetes complications	→ Hospitalization
29	Quality assessment instrument	Patient factors, Clinical factors, System factors	Medical patients	→ Hospitalization
30	SIR (structured implicit record review)	Patient factors, Clinical factors, System factors	Nursing home residents	Nursing home → Hospital, Nursing home-Emergency department
31	Rectal bleeding admission guide and algorithm	Patient factors	Acute LGIB (Acute lower gastrointestinal bleeding) surgical patients	Community → Hospital, Community → Surgical unit, Community → Emergency department
32	Potentially Avoidable Readmission (PAR) algorithm	Patient factors	Inpatients hospitalized for heart failure, acute myocardial infarction, pneumonia, or chronic obstructive pulmonary disease	→ 30-day Rehospitalizations
33	RAFT (Reducing Avoidable Facility Transfers) model	Likely Patient factors, Clinical factors, System factors	Nursing home residents	Nursing home → Hospital, Nursing home → Emergency department
34	Ottawa Heart Failure Risk Scale (OHFRS)	Patient factors	Patients with shortness of breath due to acute heart failure	→ Emergency department admissions, → Emergency department discharges
35	The Ottawa COPD (chronic obstructive pulmonary disease) Risk Scale (OCRS)	Patient factors	Patients with shortness of breath or respiratory distress caused by COPD	→ Emergency department admissions, → Emergency department discharges
36	Comprehensive Geriatric Assessment	Patient factors	Older adults, nursing home residents	→ Hospitalizations, Nursing home → Hospital, Nursing home → Emergency department
37	Standardised chart review method (with ORIGINAL trigger tool)	Patient factors	Hospital patients with multimorbidity (from 3 chronic medical conditions) and polypharmacy (from 5 chronic medications)	→ Drug-related hospital admissions (DRAs)
38	Standardised chart review method (with REVISED trigger tool)	Patient factors	Hospital patients with multimorbidity (from 3 chronic medical conditions) and polypharmacy (from 5 chronic medications)	→ Drug-related hospital admissions (DRAs)
39	Tool on appropriate referrals by Bermejo Higuera et al	Patient factors, System factors	Nursing home residents	Nursing home → Emergency department
40	Tool by Codde et al	Patient factors, System factors	Nursing home residents	Nursing home → Emergency department
41	A prediction rule to identify low-risk patients with community-acquired pneumonia (Pneumonia Severity Index, PSI)	Patient factors	Patients with community-acquired pneumonia (CAP)	→ Hospitalizations
42	Tool by Gozalo et al	Patient factors, System factors	Nursing home residents with cognitive and functional impairment	Nursing home → Hospital
43	Tool by Ong et al	Patient factors	Nursing home residents	Nursing home → Hospital
44	Modified Early Warning Score (MEWS)	Patient factors	Emergency (non)surgical patients	Emergency department → Intensive Care Unit (ICU), Emergency department → High Dependency Unit (HDU)
45	The 80 + score	Patient factors, Likely Social factors	Patients hospitalized to medical and surgical departments	→ Rehospitalizations (Emergency department admissions or readmissions)
46	The TRST	Patient factors	Patients hospitalized to medical and surgical departments, emergency department patients	→ Emergency department readmissions within 30 and 120 days after emergency department discharge, → Hospitalizations within 30 and 120 days after emergency department discharge, → Nursing home admissions within 30 and 120 days after emergency department discharge
47	ERA index	Patient factors	Hospitalized patients	→ 30-day Rehospitalizations, Hospitalizations, → ED
48	Risk prediction model for PARAs	Patient factors	Hospitalized patients who were discharged back to their place of residence	→ Rehospitalizations

The assessment tools differ whether they provide a specific outcome or have a specific judgement process. For example, the Ottawa Heart Failure Risk Scale (OHFRS) [79] provides a specific outcome, where a score is calculated based on ten criteria and the resulting score is transformed into percentage risk of serious adverse events for ED patients with acute heart failure. On the other hand, the Preventability Assessment Tool (PAT) is less specific in judgement process, which delegates a decision to a reviewer on how preventable an admission was, based on pre-defined factors such as patient, self-care, primary care, coordination of care, access to (non)clinical care, and hospital admission characteristics factors [60].

Some assessment tools also differ in what they specifically measure in the first instance, based on which a final judgement is made. For example, the CURB-65 score measures mortality risk and severity in community-acquired pneumonia, based on which recommendations are made regarding the avoidability of care transitions [40, 61]. Focus of measurement of other tools also include, but are limited to: expected probability of death (LACE index) [45, 74]; adverse health outcomes (ISAR scale) [59]; 1-year mortality (Silver code, Walter indicator) [59, 88, 89].

Assessment tools are intended for use by various people. For example, by care professionals (e.g., INTERACT´s care paths, ACE´s model evidence-based algorithms, AEP) [31–33, 46, 50]; by study researchers (LACE index, PAR-risk score, tool by Ong et al., tool by Gozalo et al.) [56, 57, 66, 74], though one tool was specifically designed for use by nursing home (NH) residents, their families, caregivers, and friends [38].

Since there is no gold standard assessment tool, there may be several tools that can be suitable in a specific situation. Hence, a decision about which assessment tool to use in a certain situation becomes a matter of choice. Box 1 presents a fictional vignette case that illustrates the application of different assessment tools and their outcomes in actual clinical practice.

Results stemming from application of the tools identified from studies included as part of category 1 are summarised as follows. The Aged Care Emergency Service (ACE) model [32, 33] seems to be promising, as it demonstrated its potential to successfully reduce hospital and ED visits of older adults with complex healthcare needs living in residential aged care facilities. The complex intervention entitled “Better Health in Residents of Care Homes with Nursing (BHiRCH-NH)” seems to be safe, since proper adverse event data collection did not reveal the intervention caused harm [30, 35]. However, despite successful recruitment and retention of participants, the study showed limited engagement of participants with the intervention tools [30, 35]. It was observed that increased use of core INTERACT tools reduced potentially avoidable hospitalizations in intervention and control skilled nursing facilities, while preserving the safety of nursing facility residents [31, 34, 37]. A study by Selker, Beshansky [36] showed that ACI-TIPI instrument has potential for substantial reductions in admissions to the Coronary Care Units (CCU), telemetry units, and hospitals, particularly in settings with high rates of overuse, without causing a negative impact on care. The evaluation of a novel decision guide “Go to the Hospital or Stay Here?” in a randomized-controlled trial observed that there were no decrease in transitions to hospital and no increase in decisional preparation, when compared with the control group [38]. On the other hand, the intervention group participants rated the guide as being very helpful and showing an increase in knowledge and decline in decisional conflicts [38]. Further information is available in Supplementary file 2 (*Characteristics of the included studies*), column “outcome”.

Overall, as can be seen from Supplementary file 8 (*Summary of tools with filter options*), half of assessment tools with reported C-statistic had values greater than 0.7, indicating good discriminatory power.

An online database was launched (www.decision4transition.com) that systematically summarises our findings and allows to instantly filter assessment tools based on their properties. The database has six major filter categories, with further filter options within each category. The database also provides a new consensus-based definition for “avoidable care transitions” [19] for overall guiding principles on avoidability of care transitions.

Box 1. A fictional vignette case For a 70-year-old male living in the community setting with community-acquired pneumonia, presence of confusion, respiratory rate of 30/min, and systolic blood pressure of 85 mm Hg, two eligible assessment tools may be used to support informed decision-making on the appropriateness of a possible hospitalisation. Namely CURB-65 for community setting and a Prediction Rule to identify low-risk patients with community-acquired pneumonia. When CURB-65 is used, the patient scores 4 out of 4 points, implying high mortality risk and urgent need for hospital admission. When the Prediction Rule assessment tool is applied using the information available, the patient scores 130 points, implying the upper boundary of risk (class IV out of five possible), and the need or appropriateness for inpatient care. However, if more data on coexisting illnesses, abnormal physical examination, or laboratory findings were available, the Prediction Rule tool would be more precise, and, thus, a more appropriate tool to be used for this case.

## Discussion

We identified 58 studies and reviewed 48 assessment tools including their sub-types that deal with avoidable care transitions.

Rates of avoidable care transitions ranged considerably among the studies, ranging from 2 to 77%. The designs, populations, and acute-care destinations varied widely.

The identified tools differed in various ways: components covered (*e.g., clinical/laboratory dimensions vs. statements*), focus (*e.g., focus on avoidable nature of care transitions vs. focus on appropriateness of care transitions*), usage by specific group of persons (*e.g., tools applied by care professionals vs. tools applied by study authors vs. tool designed for use by patients and caregivers*), the data sources used (*e.g., administrative databases vs. patient´s medical charts vs. interviews*), judgement process (*e.g., whether tools have a specific judgement process or not*), and focus of measurement (*e.g., what tools measure in the first instance, based on which a final judgement is made; such as mortality risk or adverse health outcomes*).

It has been argued that avoidability is not limited to a single factor, instead, it should include a set of various factors where each plays a distinct role in determining avoidable care transitions, such as patient, social, clinical, and system level factors [17, 26–28, 90]. With regard to this, all of the tools identified in this review are not comprehensive with respect to the dimensions covered, as they addressed only one or a few perspectives. Comparable findings are also reflected in a systematic review by Renom-Guiteras, Uhrenfeldt [14] and Kansagara, Englander [23].

Tools that focus on specific patients, conditions, or settings may have the capacity to predict avoidability for specific situations, limiting their application to these situations only. For example, as shown in Supplementary file 8 (*Summary of tools with filter options*), some assessment tools are intended for patients with specific conditions, such as pneumonia or heart failure, but their application in older adults with multimorbidity may be limited. Similarly, other tools are used for surgical patients and are focused on specific settings, such as surgical wards or emergency departments, which limit their use for general hospital patients or nursing home residents. Comparable findings are reflected in an earlier review [23]. However, in contrast to Kansagara, Englander [23], who reported that most risk prediction models have poor performance, we found that half of assessment tools with reported C-statistic had values greater than 0.70, indicating rather good discriminatory power.

In light of the aforementioned limitations, it is evident that some assessment tools are less useful in addressing avoidable care transitions. In addition, not all the tools are easily available for their use.

Although assessment tools can be useful in clinical practice, it is worth to bear in mind that they are meant to support decision-making and supplement the care professional´s judgement, instead of replacing it [17, 36, 40, 42, 78, 79]. Therefore, judgements stemming even from tools with good performance should be interpreted with care, and an ultimate decision should be made by a care professional.

This review benefits from a relatively rich pool of identified assessment tools with further critical analysis, which was not limited to a particular care setting or acute-care destination. We focused on including studies that originated from western countries, which may seem to be a limitation as we might possibly have missed some other eligible studies, as well as assessment tools. However, this is an EU-funded project that primarily focuses on European countries, and we have already expanded our search to other western countries that could be comparable to a certain extent in terms of population pyramid, level of development of their healthcare systems, and the way their health systems function. The evaluation of the included articles and tools was performed by a team of different reviewers, which may have added some subjective evaluative judgements. However, as described at the methods section, the procedures followed the recommendations by the PRISMA statement [91], which should have minimised this risk.

We do not advocate for a generalizable assessment tool that works well in most places or countries even for a specific situation (i.e., specific acute-care destination or specific condition), as countries even within unions like the EU still differ in their local context, such as reimbursement policies and financial incentives. Research literature highlights the importance of embracing multiple dimensions rather than focusing just on a few when addressing the avoidability of care transitions. In light of this, it is reasonable to conclude that an assessment tool, which includes multiple dimensions and is tailored to a local context, has greater credibility. We would, therefore, advocate for comprehensive assessment tools tailored to local contexts.

Further to launching an online database (www.decision4transition.com), we suggest replenishing it with further assessment tools identified by other reviews or added on individual basis. We believe that clinical and research communities might benefit from such an initiative.

## Conclusion

Our systematic review presents a comprehensive overview of a large number of tools addressing avoidable care transitions. The evidence generated through synthesis and appraisal is now ready to be used as a source for informed decision-making for clinical and research communities when it comes to choosing the right tool.

We noticed considerable heterogeneity among studies as well as assessment tools. Most tools were limited to a single or few perspectives that are used in the judgement process. Some assessment tools did not provide a specific judgement, but rather delegated such judgement to a reviewer by navigating over a series of items. Further research is justified in order 1) to develop multi-dimensional comprehensive assessment tools tailored to local contexts and 2) to periodically replenish the online database (www.decision4transition.com) with further assessment tools.

## Supplementary Information

Below is the link to the electronic supplementary material.

Supplementary file1 (DOCX 26 KB)

Supplementary file2 (DOCX 99 KB)

Supplementary file3 (DOCX 280 KB)

Supplementary file4 (DOCX 180 KB)

Supplementary file5 (DOCX 52 KB)

Supplementary file6 (DOCX 32 KB)

Supplementary file7 (DOCX 12 KB)

Supplementary file8 (XLSX 38 KB)

## Data Availability

Data supporting the results reported in the article are available as follows: 1) study protocol is publicly available in PROSPERO (registration number: CRD42022312516) 2) analysis data, data on included studies, data on excluded studies, final search string, risk-of-bias assessment results, PRISMA check-list, and data on assessment tools are available as online supplementary files.
