# Supplementary material for: Assessment tools addressing avoidable care transitions in older adults: a systematic literature review
Source: Eur Geriatr Med. 2024 Nov 29;15(6):1587–601. doi: 10.1007/s41999-024-01106-7 (PMC11632047; doi:10.1007/s41999-024-01106-7)
Supplement: Supplementary file 1 — Supplementary file1 (DOCX 26 KB) [file 41999_2024_1106_MOESM1_ESM.docx]

**Supplementary file 1: Analysis of tools template**

Aim: To come up with a list of quality criteria to analyse each assessment tool. Results of this analysis can serve as a source for informed decision making for clinical and research communities when it comes to choosing the right assessment tool.

Proposed criteria:

Abbreviations: Y - yes, N - no, NI – no information, NA – not applicable.

# Concepts/components covered

Provide information on concepts/components the assessment tool covers. For example, the tool can cover clinical and/or laboratory characteristics, but can also include other various concepts or components.

# What the tool measures/does

Provide information on what the assessment tool measures/does. For example, the tool can measure mortality, specific risks, etc.

# Objectivity

- 1. Process *(possible answers: Y, N, comments/short justification).*

Are instructions of the assessment tool clear? Is it clear how to use the assessment tool? Is the assessment tool self-explaining (i.e. whether the tool is structured intuitively, or the tool has explanations/instructions within the tool itself, etc.)?

- 1. Evaluation *(possible answers: Y, N, comments/short justification).*

In calculating outcome/tool result/score, is there **any** subjective judgement involved? If at least one element of a tool involves subjective judgement, then the answer is yes.

For example, the tool may have pre-defined criteria, but assessment may include element of subjective judgement (for example, when measuring level of pain; higher level of pain corresponding to higher scores). On the other hand, the tool may have pre-defined criteria, but assessment does not include any element of subjective judgement (for example, when measuring blood pressure or heart beat rate; where specific values of measurement correspond to a specific score).

- 1. Interpretation *(possible answers: Y, N, comments/short justification).*

Is there any clear guidance on how to arrive at a final assessment tool´s judgement? Is it clear how to interpret evaluation results?

A tool may have rules/thresholds of how to convert a score/evaluation result into a risk category or tool´s final judgement. For example, scores 0-2 mean a patient is at low risk of deterioration and should not be hospitalized/hospitalization could be avoided, scores 5-7 mean a patient is at high risk for deterioration and should be hospitalized. Also provide rules/thresholds on converting evaluation into tool´s judgement.

# Reliability

- 1. Inter-rater reliability *(possible answers: Y, NI. If Y, provide results as reported).*

Extent of agreement among independent observers who administered an assessment tool. Was this reported?

- 1. Intra-rater reliability *(possible answers: Y, NI. If Y, provide results as reported).*

Extent of agreement among repeated administrations of an assessment tool performed by a single observer. Was this reported?

# Validity

- 1. Convergent validity *(possible answers: Y, NI. If Y, provide brief info how it was assessed and results as reported).*

Was convergent validity reported?

“In convergent validity, we examine the degree to which the operationalization is similar to (converges on) other operationalizations that it theoretically should be similar to” (1). In simple words, in this analysis we interpret this concept of convergent validity as a sort of comparison between an assessment tool´s output/result AND a “golden standard”/observed result. We will therefore consider the following assessment types that fit the concept of convergent validity.

**Discrimination** refers to how well the assessment tool can separate one group from another (for example, avoidable vs. unavoidable care transitions).

It is commonly measured using ROC curves, and area under the ROC curve is a useful parameter summarizing ROC curve. Area under the ROC curve is equivalent to Concordance statistic (C statistic). The C statistic can also be interpreted as the rank correlation between predicted probabilities of the outcome occurring and the observed response. It can also be measured by sensitivity/specificity, PPV/NPV, Brier score (combine calibration and discrimination). (2)

**Calibration** refers to a measure of how well the predicted probabilities agree with the observed probabilities (is a property related to goodness of fit of a model). It can be measured for example by Hosmer-Lemeshow goodness of fit test, Brier score (combine calibration and discrimination). (2)

**Gold Standard.** We refer to this assessment type as a comparison of an index assessment tool to a “gold standard” (i.e. for example another assessment tool or expert consensus).

# Costs

- 1. Time to completion *(possible answers: Y, NI. If Y, provide results as reported or brief description/comment).*

Were any information on approximate time to complete the assessment tool in min or other information that may pertain to completion time reported?

- 1. Specific input data required *(possible answers: Y, N, NI, short description if Y or N).*

Are some data required as input for the assessment tool relatively time-consuming/resource-consuming to collect?

The assessment tool may require data relatively quick and cheap to collect, for example readily available data from patient´s medical record or certain quick measurements (i.e. previous hospitalizations, known comorbidities, blood pressure, heart rate). The assessment tool may also require specific time-consuming data (i.e. laboratory analyses of blood sample).

- 1. Specific training required *(possible answers: Y, N, NI, short description if Y).*

Does the assessment tool require specific training to use it?

# Who completed the tool?

Provide information on who completed the assessment tool. For example, it could be study authors, nurses, physicians, etc.

1. When/where/how the tool was/can be completed?

Provide information on when/where/how the assessment tool was completed. For example, when can refer to at admission/at discharge, where can refer to hospital/nursing home, how can refer to retrospectively/prospectively.

1. Language

Provide information on the language used in the assessment tool.

1. Tool can be seen/accessed in

Provide information on where the assessment tool can be seen/accessed.

| **Tool name (reported in…)** | |
| --- | --- |
|  | |
| **Concept/components covered** | |
|  | |
| **What the tool measures/does** | |
|  | |
| **Objectivity** | |
| **Process** |  |
| **Evaluation** |  |
| **Interpretation** |  |
| **Reliability** | |
| **Inter-rater** |  |
| **Intra-rater** |  |
| **Validity** | |
| **Convergent** |  |
| **Costs** | |
| **Time to completion** |  |
| **Specific input data required** |  |
| **Specific training required** |  |
| **Who completed the tool?** | |
|  | |
| **When/where/how tool was/can be completed? (for example: at discharge/at admission, in hospital/in nursing home, retrospective/prospective)** | |
|  | |
| **Language** | **Tool can be seen/accessed in:** |
|  |  |

1. Conjointly. Types of Measurement Validity [<https://conjointly.com/kb/measurement-validity-types/>. Accessed 06.07.2023

2. Logan B. ROC Curves and the C statistic. Datum Biostatistics NEWSLETTER Key Function of the CTSI & MCW Cancer Center Biostatistics Unit. 2013;19(4).
