# Supplementary material for: Assessment tools addressing avoidable care transitions in older adults: a systematic literature review
Source: Eur Geriatr Med. 2024 Nov 29;15(6):1587–601. doi: 10.1007/s41999-024-01106-7 (PMC11632047; doi:10.1007/s41999-024-01106-7)
Supplement: Supplementary file 2 — Supplementary file2 (DOCX 99 KB) [file 41999_2024_1106_MOESM2_ESM.docx]

**Supplementary file 2: Characteristics of the included studies**

**List of abbreviations**

RACF: Residential Aged Care Facility

ED: Emergency Department

ACE: The Aged Care Emergency program

RN: Registered Nurse

BHiRCH-NH: Better Health in Residents in Care Homes with Nursing

INTERACT: **I**nterventions to **R**educe **A**cute **C**are **T**ransfers

NH: Nursing Home

NF: Nursing Facility

SNF: Skilled Nursing Facility

AEP: Appropriateness Evaluation Protocol

HOSPITAL score: low Haemoglobin at discharge, discharge from an Oncology service, low Sodium on discharge, Procedure during hospital stay, urgent/emergent admission, number of hospital admission and Length of stay.

LACE index: Length of stay (days), Acute (emergent) admission, Charlson Comorbidity Index and number of ED visits within six months.

AUC- Area Under Curve

SAE- Serious Adverse Events

HF- Heart Failure

COPD- Chronic Obstructive Pulmonary Disease

ERA- Elders Risk Assessment

EOL-End Of Life

PAR-Potentially Avoidable Readmissions

GP- General Practitioner

ISAR- Identification of Seniors At Risk

PAT- Preventability Assessment Tool

PPH- Potentially Preventable Hospitalizations

CAP- Community Acquired Pneumonia

PSI- Pneumonia Severity Index

SIR- Structured Implicit Review

LGIB- Acute Lower Gastrointestinal Bleeding

TRST- Triage Risk Screening Tool

RAFT- Reducing Avoidable Facility Transfers

OHFRS- Ottawa Heart Failure Risk Scale

OCRS- Ottawa COPD Risk Scale

MEWS- Modified Early Warning Score

HDU- High Dependency Unit

ICU- Intensive Care Unit

CGA- Comprehensive Geriatric Assessment

TREAT- Triage and Rapid Elderly Assessment Team

LOS- Length Of Stay

ADE- Adverse Drug Event

DRA- Drug Related Admission

| **Number** | **Authors publication year** | **Country** | **Method /Design** | **Sample/Study population and number** | **Care transition** | **Assessment tool reported** | **Outcome** |
| --- | --- | --- | --- | --- | --- | --- | --- |
|  | **Category 1** | | | | | | |
| 1 | [Hullick et al. (2016)](#_ENREF_23) | Australia | Controlled pre-post design | 12 RACFs (4 RACFs for the intervention group). For each of the four intervention RACFs, two control RACFs were selected. | RACF - hospital/ED | The ACE service model has 7 key elements.  1. An ED advanced practice nurse with aged care skills  2. More than 20 evidence based algorithms  3. An education program for RACFs clinical staff  4. An ED RN led telephone consultation service for RACF staff  5. Establishment of the purpose of the ED transfer  based on the older person’s goals of care  6. proactive case management  7. A collaborative respectful relationship among organizations to achieve optimal patient outcomes. | ***1) ED presentation.***  The intervention RACFs shows higher monthly presentation values, consistent with their selection as the initial sites that might benefit the majority from such an intervention. When analysing both the impact of time and the matched controls, the non-significant parameter estimate for the Group × Time interaction suggests that patients from intervention RACFs and control RACFs had a similar change in the odds of ED presentation in any given month pre- to post-intervention.  ***2) ED length of stay.***  Control RACFs ED length of stay reduced from 496.7 min to 481.7 min while the intervention RACFs ED length of stay reduced further from 496.3 min to 435.7 min.  ***3) Hospital admission following ED presentation.***  The odds of hospital admission tended to increase (by ~35 %) from pre- to post-intervention across all RACFs, with this increase being significant (p= 0.01).  ***4) Hospital length of stay.***  The hospital length of stay tended to decrease post- intervention in both treatment groups, but to a greater extent in intervention RACFs with their length of stay reducing from 9.4 days to 6.3 days after the intervention, compared to the control RACFs that reduced from 10.0 days to 8.0 days.  ***5) 28-day hospital readmissions.***  Patients from intervention RACFs and control RACFs had a similarly negligible change in the odds of 28 day hospital admission pre- to post-intervention (OR = 1.18, p= 0.49). 28-day hospital readmission decreased in both groups, but to a lesser extent in intervention RACFs. |
| 2 | [Hullick et al. (2021)](#_ENREF_24) | Australia | A stepped wedge nonrandomized cluster trial | Nine hospital EDs and 81 RACFs that primarily transferred residents to the 9 EDs were engaged in the ACE program. | RACF - hospital/ED | The Aged Care Emergency (ACE) program has 7 key elements.  1. An ED advanced practice nurse with aged care skills  2. More than 20 evidence based algorithms  3. an education program for RACFs clinical staff  4. An ED RN led telephone consultation service for RACF staff  5. Establishment of the purpose of the ED transfer  based on the older person’s goals of care  6. proactive case management  7. A collaborative respectful relationship among organizations to achieve optimal patient outcomes | ***1) Hospital admissions.***  The average number of hospital admissions per month per 1,000 RACF bed-days was similar in the control and intervention conditions (1.03 vs 1.01). After adjusting for clustering and confounding variables, the rate for hospital admissions was .79 times the control period (i.e., a 21% reduction in the rate of hospital admission).  ***2) ED visits.***  In the crude analysis, earlier clusters had higher overall rates of transfer and admissions, with an average of 1.55 (1.26) ED visits per month per 1,000 RACF bed-days in the control condition compared with an average of 1.48 (1.16) in the post intervention condition. After adjusting for clustering and confounding variables, the ED presentation rate in the intervention period was .80 times that of the control period (i.e., a 20% reduction in the rate of ED visit). |
| 3 | [Sampson et al. (2020)](#_ENREF_41) | UK | Pilot cluster randomised controlled trial | 12 nursing homes (7 in West Yorkshire and 5 in Greater London). 5 NHs in intervention and 7 NHs in control groups. | Avoidable hospital admissions | BHiRCH-NH intervention consisted of 3 items adapted from the INTERACT programme: Stop and Watch early warning tool (S&W), Care pathway, The situation, background, assessment recommendation (SBAR). | This was a pilot-trial and analyses were mainly descriptive. Despite excellent recruitment and retention, the limited engagement with the intervention tools and support for their implementation in the pilot trial has led the authors to conclude that a definitive trial of this intervention is not warranted. |
| 4 | [Downs et al. (2021)](#_ENREF_16)  *This is a report on a research programme that includes the publication on pilot cluster RCT by Sampson et al. (2020), which appears to be the focus of this report.* |  |  |  |  |  |  |
| 5 | [Kane et al. (2017)](#_ENREF_27)  *This is the primary paper with RCT. This paper by Kane et al. 2017, and other two by Tappen et al. 2018 and Huckfeldt et al. 2018 are considered as 1 study with 3 publications.* | USA | Cluster randomized trial | A sample size of 9050 and 8380 residents in intervention NHs in the pre-intervention and intervention periods, respectively, and 14 428 and 13 472 residents in control NHs in the pre intervention and intervention periods, respectively. | NH - hospital/ED | INTERACT program is based on 3 core tenets: (1) recognition and management of acute conditions before they become severe enough to require hospitalization; (2) providing communication, documentation, and decision support tools that allow for effective management in the NH without hospital admission when safe and feasible; and (3) emphasizing advance care planning, hospice, and palliative care to encourage goals of care discussions and reduce hospitalizations in people with end-stage illness among whom the risks and discomforts of hospital care often outweigh the benefits. | ***1) Rate of hospitalizations per 1000 resident-days****.*  Both intervention and control NHs exhibited between 3 and 4 hospitalizations per 1000 resident-days in most months of the sample period. For a facility with a census of 100, this rate translates to 3 or 4 residents being admitted to the hospital every 10 days.  ***2) Potentially avoidable hospitalizations.***  The intervention NHs exhibited a slightly higher rate of potentially avoidable hospitalizations in the pre-intervention period and converged with control NHs during the intervention period.  **3)** ***ED visits rates without admission.*** Trends for ED visits without admission were also very similar between the intervention and control groups.  **4)** ***Hospitalization rates***. There was no significant reduction in hospitalizations within 30 days of NH admission or 31 or more days after NH admission. |
| 6 | [Tappen et al. (2018)](#_ENREF_50) | USA | Secondary analysis of a randomized controlled implementation trial | 264 NFs randomized into intervention and comparison groups Intervention NFs (n=88) Control NFs (n=176) | NF-hospital | The INTERACT Quality Improvement Program: involves a set of tools and resources designed to address the primary reasons for potentially avoidable hospital admissions of NF residents. | **1)** ***Resident-quarter level safety measures using the MDS for the following measures: unintentional weight loss (exclusive of physician-prescribed weight-loss), malnutrition, hip fracture, pneumonia, wound infection, septicemia, urinary tract infection, and falls resulting in injury (minor or major).***  Across all measures, there were no statistically significant differences in the percentage of resident-quarters exhibiting MDS-derived safety measures between the intervention and control groups in either the pre-intervention or intervention years.  **2)** ***Whether the following items increased, decreased or remained the same over the last month: unintentional weight loss, dehydration, incidence of falls, new pressure ulcers, severe pain, and unexpected deaths.***  There were no statistically significant increases in safety indicators over time [fall rates (P=.321), new pressure ulcers (P=.274), severe pain (P=.687), weight loss (P=.946), or dehydration (P=.661)].  There were also no statistically significant changes in safety measures by level of engagement across the 12 months of INTERACT implementation for weight loss [F (2, 141.48) = 0.52, P=.471], dehydration [F(2, 127.33)=2.32, P=.130], fall rates [F(2, 109.21)=0.28, P=.559], or pressure ulcer [F(2, 109.71)=0.21, P=.652]. There was insufficient variability in unexpected deaths for analysis. However, there were statistically significant differences by engagement group for severe pain [F (2, 153.31)=2.90, P=.005] with the highly engaged(engagement group 3) reporting significantly lower incidence of severe pain when compared to the low engagement (group 1) [b=0.11, t(201.45)=1.99, P=.012] and moderate engagement (group 2) [b=0.12, t(204.67)=2.40, P=.002]. |
| 7 | [Huckfeldt et al. (2018)](#_ENREF_22) | USA | Secondary analysis from a randomized controlled trial | Skilled nursing facilities (N = 264) | SNF- hospital/ED | The Interventions to Reduce Acute Care Transfers (INTERACT) program includes a set of tools that address factors leading to avoidable hospital admissions and ED visits of SNF residents. INTERACT has 7 tools in total: “Stop and Watch”; Situation, Background, Assessment, Recommendation (SBAR) Communication Form and Progress Note; Hospitalization Tracking tool; root-cause analysis Quality Improvement Review tool; Hospital Transfer Form; decision support tools (Care Paths, Change in Condition File Cards); and Advance Care Planning tools. | ***1) Hospitalizations and potentially avoidable hospitalizations.***  The increased-use group had relative reductions of 11.2% in all-cause hospitalizations and 18.9% in PAHs (potentially avoidable hospitalizations) (both p<.001), whereas the low-use group had nonsignificant relative reductions of 1.6% in all-cause hospitalizations and 4.8% in PAHs. |
| 8 | [Selker et al. (1998)](#_ENREF_43) | USA | Controlled clinical trial | ED patients with chest pain. 10689 patients: 4738 intervention group, 5951 control group. | Hospital and CCU (coronary care unit) admissions | Acute cardiac ischemia time-insensitive predictive instrument (ACI-TIPI) (software based). | ***Emergency department triage to a coronary care unit (CCU), telemetry unit, ward, or home:***  **1)** For patients without cardiac ischemia, in hospitals with high-capacity CCUs and relatively low-capacity cardiac telemetry units, use of ACI-TIPI was associated with a reduction in CCU admissions from 15% to 12%, a change of -16%, and an increase in emergency department discharges to home from 49% to 52%, a change of 6%.  **2)** Across all hospitals, for patients evaluated by unsupervised residents, use of ACI-TIPI was associated with a reduction in CCU admissions from 14% to 10%, a change of -32%; a reduction in telemetry unit admissions from 39% to 31%, a change of -20%, and an increase in discharges to home from 45% to 56%, a change of 25%.  **3)** Among patients with stable angina, in hospitals with high-capacity CCUs, use of ACI-TIPI was associated with a reduction in CCU admissions from 26% to 13%, a change of -50%, and an increase in discharges to home from 20% to 22%, a change of 10%.  **4)** At hospitals with high-capacity telemetry units, use of ACI-TIPI was associated with a reduction in telemetry unit admissions from 68% to 59%, a change of -14%, and an increase in emergency department discharges to home from 10% to 21%, a change of 100%.  **5)** Among patients with acute myocardial infarction or unstable angina, use of ACI-TIPI did not change appropriate admission (96%) to the CCU or telemetry unit at hospitals with high-capacity CCUs or telemetry units. |
| 9 | [Tappen et al. (2020)](#_ENREF_51) | USA | Mixed method RCT with qualitative data embedded in the quantitative data | Nursing home residents and family members from 15 long-term care facilities. 192 participants (128 residents and 64 family members). | NH-hospital | Novel Decision Guide "Go to the Hospital or Stay Here?" for Nursing Home Residents and Families. | ***1) Effectiveness of the use of the guide in increasing the perception of preparation for decision making and reducing decisional conflict (change in the intervention group from pretest to posttest).***    Decisional conflict was significantly lower at posttest with an average of 9,98 compared to the pretest with an average of 13,11.  ***2) Differences on the posttest scores in decisional conflict between control and intervention groups.***  The intervention group had statistically significant lower decisional conflict (8,95) compared to the control group.  ***3) Effectiveness of the decision aid in improving residents´ and family members´ knowledge over time compared to the control group.***  Of individuals who completed the pretest and posttest, there were statistically significant improvements overall for both groups, but the intervention group showed a greater improvement in knowledge from pretest to posttest compared to the control group.  ***4) Differences in rehospitalization rates between groups.***  There were few rehospitalizations in the sample, 5 for the control group and 8 for intervention group. The difference in numbers of transfers from the NH to an acute care facility was not statistically significant across groups. |

| **#** | **Authors publication year** | **Country** | **Method/Design** | **Sample/Study population and number** | **Care transition** | **Assessment tool reported** | **Outcome** |
| --- | --- | --- | --- | --- | --- | --- | --- |
|  | **Category 2** | | | | | | |
| 1 | [Abdoulhadi et al. (2015)](#_ENREF_1) | France | Retrospective descriptive study | 40 Patients from NHs, referred directly by their attending physician or the NH's coordinating physician to a geriatric short-stay medical service. | NH - acute geriatric unit | 1. They used AEPf - a French version of the AEP 2. They also proposed AEPg - geriatric adaptation of AEP | ***Appropriateness of admissions.***  **1.** AEPf according to AEPf 21 admissions (52,5%) are relevant. Expert jury justified 12 admissions (30%).  **2.** AEPg according to AEPg 31 admissions (77,5%) are relevant. Expert jury justified 2 admissions (5%). |
| 2 | [Aliberti et al. (2011)](#_ENREF_2) | Italy | An observational, retrospective study | N=580 patients. Patients >= 18 years of age and satisfying the criteria for CAP were included in this study. | Hospitalizations | CURB-65 score | ***1) The CURB-65 score was calculated for every patient who referred to the ER during the study period. Appropriateness of hospitalizations.***  Out of 580 patients included in the study, 218 patients were classified with a CURB-65 score of 0 or 1 on admission to the ER, and among them 127 (58%) were hospitalized (Group 1), while 91 were sent home (Group 2). Among the 127 patients belonging to Group 1, reasons that justified hospitalization were found in 104 (83%) patients. No clinical justification for hospitalization was identified in 23 patients (17%).  A total of 362 patients were classified with a CURB-65 score of 2-to-5 on admission to the ER, and among them 360 (99%) were hospitalized, while 2 were sent home. Among patients with CURB-65 score of 2-to-5 who were hospitalized, 54 (15%) patients died. |
| 3 | [Almeida et al. (2006)](#_ENREF_3) | Portugal | Routine data analysis | 690 admissions/patients, 975 hospitalization days | Hospitalizations | Adapted AEP | ***1) Appropriateness of admissions.***  170 (24.6%) admissions are inappropriate 520 (75,4) admissions are appropriate.  ***2) Appropriateness of hospitalization days.***  365 (37.4%) inappropriate hospitalization days 610 (62,6%) appropriate hospitalization days. |
| 4 | [Arendts et al. (2015)](#_ENREF_4) | Australia | Prospective cohort study | 1143 patients. Patients aged 65 years or over who had been medically assessed in the ED and designated for discharge back to the community. | Unplanned ED revisit within 28 days from discharge | Risk Nomogram | **1)** ***Any visit to an ED within 28 days of discharge, excluding planned reviews.***  Overall revisit rate 28.4%. In 1143 patients, the odds of revisit increases progressively with increasing strata of predicted risk, culminating in an OR of 9.7 (95 % CI 4.7–19.9) in the highest risk group.  The 28-day revisit rates across strata range from 16 % through 65 %, with the difference between strata being statistically highly significant (p<0.001). |
| 5 | [Attena et al. (2001)](#_ENREF_5) | Italy | Concurrent method (survey) | 533 admissions | Hospitalizations | AEP Italian version | **1)** ***Appropriateness of admissions.***  84 (15.8%) admissions were considered inappropriate.  ***2)*** ***Appropriateness of hospitalization days.***  170 (35.5%) index days of stay were considered inappropriate. |
| 6 | [Aubert et al. (2017)](#_ENREF_6) | USA, Canada, Switzerland, Israel | Retrospective study | 117,065 discharges. Consecutive medical patients discharged from each participating hospital. | 30 day potentially avoidable readmissions | 1) HOSPITAL score 2) Simplified HOSPITAL score | **1)** ***30-day potentially avoidable readmissions.***  The simplified HOSPITAL score classified 70.4% (n=82,383) discharges as unlikely, and 29.6% (n=34,682) as likely to be followed by a 30-day potentially avoidable readmission. The percentage of discharges followed by a potentially avoidable readmission was 6.4% in the low-risk category and 17.3% in the high-risk category.  **2)** ***Any 30-day readmissions.***  Overall, 29.6% of the patients were classified as high-risk, and 27.2% of them had any 30-day readmission. |
| 7 | [Baig et al. (2018)](#_ENREF_7) | New Zealand | Admissions data analysis | 180,118 admissions. Adult admissions from three hospitals. | Hospital readmissions | 1. LACE Index for Readmission - Length of stay (days), Acute (emergent) admission, Charlson Comorbidity Index and number of ED visits within six months.    2. New Zealand version of Patients At Risk of Hospital Readmission (PARR) using admissions data from the New Zealand hospitals. | ***AUC score in predicting 30 day readmissions.***  **1)** The LACE index achieved an AUC score of 0.658 in predicting 30- day readmissions.  **2)** The PARR algorithm achieved an AUC score of 0.628 in predicting 30-day readmissions |
| 8 | [Baré et al. (1995)](#_ENREF_8) | Spain | Cross sectional study (a retrospective analysis of patients´ medical records using AEP) | 639 medical records of adult patients admitted to a hospital were reviewed | Hospitalizations | AEP | **1)** ***Inappropriate hospital admissions.***  The rate of inappropriate hospital admissions was 9.1% (58/639).  **2)** ***Inappropriate hospitalization days.***  Overall 29.2% of hospitalization days (1963/6731) were inappropriate.  **3)** ***Reasons for (in)appropriate admissions.***  Inappropriate admissions were primarily attributable to hospitalizations for diagnostic and/or therapeutic services that could have been rendered on an ambulatory basis (70.7% of cases).  In the group of 581 appropriate admissions, about 47% of the reasons were surgery or use of facilities available only in the hospital, followed by intravenous medications and/or fluid replacement in 18.2% of cases, acute or progressive cardiorespiratory failure in 14.5% and persistent fever > 38º for more than five days in 8.1%.  **4)** ***Reasons for unnecessary days of care.***  Inappropriate admissions to hospital, and optimizable health care planning and a conservative physician's attitude (postponed discharge) were the most frequent reasons for unnecessary days of care. |
| 9 | [Bermejo Higuera Jc (2010)](#_ENREF_9) | Spain | Descriptive retrospective cross-sectional study | Older people who were referred from a nursing home (intermediate care unit) to the Hospital University. 45 residents (62 referrals in total) | NH- ED | Tool on appropriate referrals by Bermejo Higuera et al | ***1) Appropriateness of referrals.***  98.4% of referrals met one or more of the appropriateness criteria. 67.7% met criterion 1, 20.97% met criterion 2, 77.4% met criterion 3. (referral is deemed appropriate if at least 1 of 3 criteria addressed by a tool is met)  ***2) Causes of referrals.***  The most common causes of referral were: suspected post-fall fracture (20 cases, 32.3%) exacerbation of respiratory disease (7 cases, 11.3%); treatment of anaemia syndrome (transfusion) (5 cases, 8.1%); poor general condition (malaise, asthenia, 5 cases 8.1%); digestive and or bowel rhythm disturbances (abdominal pain, repeated vomiting 4 cases, 6.5%); blood glucose disturbances (3 cases, 4.8%) and acute functional impairment (3 cases, 4.8%). |
| 10 | [Burke et al. (2017)](#_ENREF_10) | USA | Retrospective cohort study | Total of 9181 patients. PPR group (1252, 13.6%), No PPR group (7929, 86.4%). Medical inpatients discharged from 6 hospitals. | Potentially preventable 30-day readmissions | HOSPITAL score | ***1) Potentially avoidable readmissions.***  9181 patients were discharged after treatment for one of the HRRP-targeted conditions [pneumonia (n = 3335, 4.2%), HF (n = 3189, 4.0%), COPD (n = 1890, 2.4%), acute myocardial infarction (n = 767, 1.0%)]. The potentially avoidable readmission rate across these diagnoses was 13.6% overall. |
| 11 | [Codde et al. (2010)](#_ENREF_11) | Australia | Retrospective medical chart review descriptive study analysing data from a single tertiary hospital ED patient database | 603 discharges. Patients discharged from Emergency Department to their Residential Aged Care Facilities without admission to an observation ward in Emergency Department or admission to Hospital. | RACF- ED | Tool by Codde et al. List of Exclusion criteria and potentially avoidable reasons for emergency department (ED) presentation | ***1) Avoidable ED presentations.***  Of the 603 discharged cases, 235 were reviewed (39%). In total, 161 of these were coded as potentially avoidable. Assuming a representative sample, this equates to 69% of discharged patients, and 31% of total transfers, as potentially avoidable. |
| 12 | [Davido et al. (1991)](#_ENREF_12) | France | Analysis of data taken from patient records and/or patients themselves. | 371 patients. Patients admitted to a hospital through the medical emergency department. | Hospitalizations | AEPf (French version) | ***1) Inappropriate admissions.***  25% prevalence of inappropriate admissions. |
| 13 | [De Giorgi et al. (2016)](#_ENREF_13) | Italy | A retrospective, observational, cross-sectional study | 613 readmitted patients | 30 day potentially avoidable rehospitalizations | 1. HOSPITAL score  2. Elders Risk Assessment (ERA) index | ***1) Death (in-hospital mortality, and death at the end of follow-up).***  Death during readmission was recorded in 110 patients (17.9%), and death at the end of follow-up in 366 (59.7%).  ***2) Avoidable and non-avoidable 30-day readmissions***  Re-hospitalization could be classified as avoidable in 286 cases (46.7%). |
| 14 | [Donzé et al. (2013)](#_ENREF_14) | USA | Retrospective cohort study | 7123 unique patients accounted for all 9212 index discharges. Patient discharges from all medical services of the Brigham and Women’s Hospital. | 30 day potentially avoidable hospital readmissions | HOSPITAL score | ***1) Estimated probability and observed proportion of potentially avoidable readmissions.***  The risk of potentially avoidable readmission was stratified into 3 categories: low, intermediate, and high. Low-risk patients with 0 to 4 points (49.3% of patients) had a 5.2% estimated risk of potentially avoidable readmission and an observed proportion of 5.4% in the derivation set; high-risk patients with 7 or more points (24.4% of patients) had an 18.3% estimated probability of potentially avoidable readmission and an observed probability of 18.7%. |
| 15 | [Donzé et al. (2016)](#_ENREF_15) | USA, Canada, Israel, Switzerland | Multicentre multinational retrospective cohort study | 9 hospitals in 4 countries 7 are university hospitals and 2 are community hospitals. 117 065 patients. Patients discharged alive from the medical services of 9 hospitals in 4 different countries. | 30 day potentially avoidable hospital readmissions | HOSPITAL score | ***1) Estimated probability and observed proportion of potentially avoidable readmissions.***  Estimated risk of potentially avoidable readmission calculated with the HOSPITAL score matched the observed proportion of potentially avoidable readmissions in each risk group: 5.8% for the low-risk group; 11.9%, intermediate; and 22.8%, high risk. |
| 16 | [Duflos et al. (2017)](#_ENREF_17) | France | Prospective study | 500 referrals related to 423 patients. Prospectively included consecutive patients who visited the ED for a medical examination. | Hospitalizations | AEPf (French version) | ***1) Appropriateness of admissions.***  Among the 288 admissions, 45 (15.6%) were potentially avoidable with high likelihood, 47 (16.3%) were potentially avoidable with moderate likelihood, and 196 (68.1%) were unavoidable. |
| 17 | [Fine et al. (1997)](#_ENREF_18) | USA | Analysis of data from a hospital database | Patients with pneumonia.  1. MEDISGROUPS DERIVATION COHORT (N=14,199) patients. 2. MEDISGROUPS VALIDATION COHORT (N = 38,039) patients. 3. PNEUMONIA PORT VALIDATION COHORT TOTAL (N=2,287) patients. | Hospitalizations | A prediction rule to identify low-risk patients with community-acquired pneumonia | ***1) Mortality.***  No significant differences in mortality in each of the five risk classes were found among the three study cohorts. Mortality was low for risk classes I, II, and III, ranging from 0.1 to 0.4 percent for class I, from 0.6 to 0.7 percent for class II, and from 0.9 to 2.8 percent for class III.  ***2) Hospitalizations.***  Among outpatients, the rate of subsequent hospitalization within 30 days ranged from 5.1 percent for class I patients to 20.0 percent for class IV. None of the 62 class I, II, or III outpatients who were subsequently hospitalized died, and only 1 was admitted to an intensive care unit. Of the eight outpatients in classes IV or V who were subsequently hospitalized, three died and one was admitted to an intensive care unit. Among inpatients, admissions to intensive care units ranged from 4.3 percent for class I to 17.3 percent for class V. For all 1236 inpatients who were discharged alive, the proportion who stayed in the hospital three days or fewer was 26.1 percent for class I and 3.7 percent for class V. |
| 18 | [Gozalo et al. (2011)](#_ENREF_19) | USA | MDS data and Demicare claims data analysis | 474,829 nursing home residents. | Nursing home - Hospital | Tool by Gozalo et al. on Three types of transitions that were classified as being potentially burdensome. | ***1) Burdensome transitions.***  A total of 90,228 nursing home residents (19.0%) had at least one burdensome transition in the last 90 days of life. The distribution of the type of burdensome transition was as follows: 55,039 subjects (11.6%) had a health care transition in the last 3 days of life, 12,827 (2.7%) had a lack of continuity in nursing home provider after a hospitalization in the last 90 days of life, and 38,573 (8.1%) had multiple hospitalizations in the last 90 of life. |
| 19 | [Higi et al. (2021)](#_ENREF_20) | Switzerland | Cohort study | 5985 internal medicine patients. | 30-day potentially avoidable hospital readmission from patient´s homes. | PAR-Risk Score | ***1) 30-day potentially avoidable hospital readmission (PAR).***  Of the eligible patients, 340 patients (5.7%) were identified as having experienced a PAR by the SQLape software, whereas it was 562 (7.7%) in the derivation patient cohort. |
| 20 | [Horey et al. (2012)](#_ENREF_21) | Australia | Data sources included interviews with RACF staff and GPs, RACF manager surveys, and the prospective audits of deaths and EOL care pathway use. | 14 RACFs with a total of 1033 resident places. | RACF- Hospital | EOL care pathway.  Liverpool Care Pathway for the Dying Patient, adapted and evaluated for use in Australian RACFs. | ***1) Acceptability.***  Pathways were used 63 times (36% of all deaths and 43% of deaths when sudden deaths not on pathways were excluded). There were three levels of uptake of EOL care pathways across the 14 RACFs. A high-uptake group (four RACFs) used pathways for 68% of all deaths (93% of deaths when sudden deaths not on pathways were excluded); a moderate-uptake group (six RACFs) used pathways for 34% of all deaths (41% when sudden deaths not on pathways were excluded); and a low- or no-uptake group (four RACFs) used pathways for 10% of all deaths (11% when sudden deaths not on pathways were excluded).  ***2) Feasibility.***  The proportion of deaths in hospital and RACFs remained constant in pre- and post- implementation manager surveys, but significantly fewer residents were transferred to hospital and subsequently returned to the RACF at the end of the project.  RACF managers reported that before the introduction of pathways, their reviews of resident records revealed that little was written about the care provided. The pathways encouraged documentation, and the audits demonstrated that care for residents on pathways was consistent with best practice at EOL, regardless of an RACF’s level of pathway uptake.  Almost all people on a pathway had appropriate medicines ordered as needed. Non- essential medicines were discontinued for 76% of those on pathways, and inappropriate interventions and observations were discontinued for 60% of those on pathways. |
| 21 | [Inzitari et al. (2015)](#_ENREF_25) | Spain | Cohort study | 265 patients. Patients consecutively transferred to the SCU of Parc Sanitari Pere Virgili from the ED of Vall d’Hebron University Hospital in Barcelona. | Different discharge destination were considered. Discharge to the usual living situation (home or usual nursing home) versus a different discharge destination (death, return to the acute hospital, or transfer to long-term nursing care). | (1) The Identification of Seniors at Risk (ISAR) scale. (2) the Silver Code (3) the Walter indicator | ***1) Discharge to the usual living situation (home or usual nursing home) versus a different discharge destination (death, return to the acute hospital, or transfer to long-term nursing care).***  Of 265 patients, 80.8% were discharged to the previous living situation, whereas 11.7% were transferred to long-term care, 3.4% returned to the ED, and 4.2% died. |
| 22 | [Johnston et al. (2020)](#_ENREF_26) | Australia | Routine data analysis, prospective study.  Patient questionnaire, PAT (preventability assessment tool), GP (family physician interview), extraction of hospital clinical data. | 275 patients. Community dwelling patients with unplanned admissions to three hospitals in NSW, Australia, with a primary discharge diagnosis of COPD, CHF, angina pectoris or diabetes complications. | PPH (potentially preventable hospitalizations) for patients living in the community. | Preventability Assessment Tool (PAT) | ***1) Assessments made by using PAT and assessment of the expert panel.***  There was low agreement between the assessments of the hospital doctors and nurses regarding which admissions were deemed preventable (Κ= 0.21; 95% CI = 0.09–0.34). The agreement between hospital nurses and hospital doctors for admissions being preventable was only 18% although agreement for non-preventable admissions (including admissions assessed as not preventable and those unclassifiable) was higher at 46%. Overall disagreement between the hospital nurses and hospital doctors was 36%. There was very low agreement between the Expert Panels and the hospital nurse regarding the assessment of the preventability of individual admissions (K= 0.17; 95% CI = 0.05–0.28). Of the 119 admissions assessed as preventable by Expert Panel, only 53 (45%) were assessed as preventable by the hospital nurses. Similarly, there was very low agreement between the Expert Panel and the hospital doctor regarding the assessment of the preventability of individual admissions (K= 0.13; 95% CI = 0.01–0.25). Of the 119 admissions assessed as preventable by Expert Panel, only 51 (45%) were assessed as preventable by the hospital doctors. |
| 23 | [Karmakar and Wilsher (2010)](#_ENREF_28) | New Zealand | Retrospective cohort study | 174 patients. Hospital patients with a discharge diagnosis of pneumonia. | Hospitalizations | The CURB 65 score | ***1) Using the score patients were grouped into 3 categories: Mild CAP (CURB 65 score 0–1) Moderate CAP (CURB 65 score 2) Severe CAP (CURB 65 score 3 or more).***  Ninety-one patients appeared to have mild CAP with a score of 0–1. Twelve of these patients were discharged from the emergency department or admitting unit without formal admission, but the rest were admitted with an average length of stay of 5.5 days. No significant reason to justify hospital admission was identified for 23 of those 52 patients. One died in this group. Fifty-three (30.5%) patients appeared to have moderate CAP with CURB 65 score of 2. All were admitted with a mean hospital stay of 8.2 days (1–25 days) and two required ICU admission. There was one in-hospital death. Thirty patients appeared to have severe CAP (CURB 65 >= 3) and most had multiple comorbidities and poor level of function precluding admission to ICU (Table 4). One patient with no such comorbidities was referred to the ICU. The average length of hospital stay was 9 days. There were four in-hospital deaths. |
| 24 | [Lamb et al. (2011)](#_ENREF_29) | USA | Observational study; qualitative and quantitative analysis | 26 NHs. Site coordinators and staff who participated in project orientation and conference calls and completed QI tools. | NH- Hospital | QI review tool (project INTERACT II) | ***1) Reasons for transfer.***  The most common reasons for transfers that were rated avoidable or possibly avoidable were in the categories of missed opportunities for preventing the transfer before or after the onset of symptoms (31.9%); resident or family insistence on transfer (13.9%); communication gaps between nursing staff, families, PCPs, specialists, and out-side facilities (13.0%); advance directives and end-of-life care not in place or not followed (11.1%); and gaps in staff knowledge or skill (9.7%). |
| 25 | [Lázaro Cebas et al. (2022)](#_ENREF_30) | Spain | A single-centre study with an intervention group and a retrospective control group. | A total of 589 of hospitalized and polymedicated patients were included in the present study: 286 patients in the intervention group and 303 in the control group intervention. | Readmissions | HOSPITAL score | ***1) 30-day readmissions intervention VS. control group.***  The 30-day readmission rate was 20.13% (n=61) in the control group and 16.43% (n=47) in the intervention group.  ***2) 30-day readmissions in 3 subgroups classified according to the HOSPITAL score.***  In the subgroup of patients with a low risk of potentially avoidable readmission, the 30-day readmission rate was similar between the control and intervention groups (11.89% vs 12.43%). In the subgroups of patients with intermediate and high risk of potentially avoidable readmission, reductions in readmission rates were observed in the intervention group. |
| 26 | [Migliorati et al. (2006)](#_ENREF_31) | Italy | Retrospective review of medical records | 148 patients, discharged with the diagnosis of pneumonia or a pneumonia-related disease. | Unnecessary hospital admissions | 1. Pneumonia Severity Index (PSI)  2. Modified Italian Appropriateness Evaluation Protocol (AEP) | ***1) Survival rate at 30 days.***  The overall survival rate at 30 days was 87.8%. The survival rate was, respectively, 86% and 92% in the groups of patients with appropriate and inappropriate admission according to the modified AEP.  ***2) Appropriateness of admissions and hospital stay.***  Overall, in accordance to the modified AEP, 52 (35%) hospital admissions were not appropriate; of these, 21 (54%) occurred in patients with low-risk class according to the PSI.  According to the modified AEP, the appropriateness of the hospital stay occurred in 45% and 63% of days, respectively, for the groups of patients with low- and high-risk PSI score. |
| 27 | [Oddone et al. (1996)](#_ENREF_32) | USA | Surveys | Phase 1, 156 patients admitted to the general medicine service at the Durham VAMC. Phase 2, 514 patients accounting for 811 readmissions within 6 months of a general medicine service discharge at nine VAMCs. | Hospitalizations | Quality assessment instrument | ***1) Preventability of readmissions.***  In phase I, residents and attending physicians rated 33% and 34% of admissions as preventable, respectively. In phase 2, 277 (34%) of 811 readmissions were deemed preventable. |
| 28 | [Ong et al. (2011)](#_ENREF_33) | UK | Retrospective case analysis | 3772 acute hospital admissions from care homes. | Care home to hospital | Tool by Ong et al., on time to death as an indication of the inappropriateness of admissions | ***1) Mortality.***  Of the 340 admissions, 93 died during their index admission (27.3%), 15 care home residents died within 24 h of admission, accounting for a significant proportion of the 38 (40.8%) who died within 3 days of hospital admission. Of the 93 deaths 16.1% (15) were within 4–7 days, and 43% (40) occurred at 8–28 days. The most common causes of death taken from death certificates included pneumonia (31.5%), stroke (21.0%) and heart failure (13.5%).  ***2) Reasons for hospitalizations.***  The most cited reasons for admission were the lack of advance care plans, access to General Practitioners (GPs) out of hours, as well as general access to palliative care and specialist nurses, and poor communication between patient, relatives, GPs, hospitals and care home staff |
| 29 | [Ouslander et al. (2014)](#_ENREF_34) | USA | An overview of the INTERACT program for medical directors and primary care clinicians in long term care | - | NH-hospital | INTERACT tools | - |
| 30 | [Ouslander et al. (2011)](#_ENREF_35) | USA | Surveys | 25 NHs | Hospitalizations | INTERACT II tools | ***1) Hospitalization rates.***  The 25 NHs that completed the 6-month INTERACT II intervention had a 17% reduction in hospitalization rates, representing a mean absolute reduction of 0.69 (1.47) hospitalizations per 1,000 resident days (P= 0.02 relative to comparison NHs). Engaged NHs had the highest reduction (24%, P=0.01 relative to comparison NHs), representing a mean absolute reduction of 0.90 (1.28) hospitalizations per 1,000 resident days. NHs that were not engaged had only a 6% reduction. The 11 comparison facilities had a 3% reduction in hospitalization rates (from 2.69 to 2.61 hospitalizations per 1,000 resident days).  ***2) Costs of the INTERACT II intervention.***  When combining costs borne by the study team and costs borne by the facility, the estimated average total cost of the 6-month INTERACT II intervention was approximately $7,700 per facility. |
| 31 | [Ouslander et al. (2009)](#_ENREF_36) | USA | Prospective quality improvement initiative | 3 NHs | NH- hospital | 1) INTERACT intervention which contains tools 2) SIR structured implicit record review | ***1. Hospitalizations, number and per 1000 resident days.***  Compared with baseline, the facilities had a 58%, 44%, and 36% reduction in hospitalizations per 1000 resident days; the overall reduction in the 3 facilities combined was 50%. The average hospitalization rate during the intervention for the 3 pilot facilities (1.54/1000 resident days) was slightly lower than the average rate for all 377 Georgia NHs in the baseline phase (1.62/1000 resident days).  ***2. Potentially avoidable hospitalizations.***  The baseline rate of potentially avoidable hospitalizations was 77% of the 30 hospitalizations reviewed in the 3 pilot facilities (compared with 68% for all 200 hospitalizations rated during baseline). Thus, the intervention was associated with a 28% absolute reduction in hospitalizations rated as potentially avoidable by the Expert Panel (77% to 49%). This represents a relative reduction of 36% (28%/77%).  ***3. Reasons for avoidable hospitalizations, and factors that could have enhanced NHs´ ability to prevent hospitalization.***  Factors frequently considered somewhat or very important for rating the hospitalization as potentially avoidable included the availability of on-site physician care, the availability of registered nurses and nurse practitioners or physician assistants, the overall quality of NH care related to assessing and managing changes in condition, and the need for better advance care planning. Re- sources frequently rated as potentially helpful in preventing avoidable hospitalizations included greater on-site availability of physician or nurse practitioner or physician assistants, more registered nurses providing care, availability of lab results within 3 hours, and the capability of the NH to administer intravenous fluids.  ***4. Diagnoses associated with potentially avoidable hospitalizations.***  When compared with the distribution of diagnoses for the 105 potentially avoidable hospitalizations for which data were available among the 200 hospitalizations reviewed for baseline data, the proportion of hospitalizations related to de- hydration/metabolic disturbances and gastrointestinal conditions were substantially higher (16% versus 7%, and 19% versus 7%, respectively). |
| 32 | [Patel et al. (2014)](#_ENREF_37) | UK | Retrospective for prealgorithm (i.e. developing a tool) prospective for postalgorithm (evaluation) | 57 patients presenting to the single institution with acute LGIB. This included all community and ED referrals. | Community-Hospital, Community-Surgical Unit, Community-ED | Rectal bleeding admission guide and algorithm | ***1) Avoidable admissions.***  Thirty-seven percent (21/57) of patients met all three of the criteria of the scoring system, indicating they could be treated without admission. Ninety-five per cent of these patients (20/21) were discharged home without hospital stay meaning that 35% (20/57) of potential admissions were avoided. One patient was admitted although he met the criteria for discharge as he was elderly and presentation was late at night. One patient was subsequently readmitted at the time of outpatient endoscopy with a diagnosis of severe colitis of potential admissions were avoided. Sixty-five per cent (36/57) did not meet the scoring criteria for outpatient treatment and were admitted to hospital.  ***2) Patient satisfaction.***  There were 10 responses to the patient satisfaction questionnaire: 80% rated the service as excellent and 10% as good; 70% rated the speed of service good or excellent; 90% felt they were given satisfactory information; and 80% knew who to contact if they had concerns. |
| 33 | [Pérès et al. (2002)](#_ENREF_38) | France | Prospective, routine data analysis, phone calls | 322 patients consecutively discharged from an acute geriatric unit. | Rehospitalizations | AEPf (French version) | ***1) Incidence of rehospitalization.***  In total, 50 rehospitalizations at one month were recorded, representing an overall incidence of 16.2%, among the 309 subjects followed.  ***2) Avoidable rehospitalizations according to the AEPf criteria.***  Of the 21 readmissions that took place in the same department, 5 (23.8%) were deemed avoidable according to the AEPf criteria. |
| 34 | [Popejoy et al. (2019)](#_ENREF_39) | USA | Cross-sectional descriptive study | 16 nursing homes (NHs). 5168 residents. | Nursing home- Hospital | INTERACT QI Acute Care Transfers (ACTs) tool, v.3.0. | ***1) Avoidable and unavoidable hospital transfers.***  There were 1516 ACTs submitted in 2014, 1336 in 2015, and 1144 in 2016. Over one-half of transfers (n = 2112 [54%]) were identified as avoidable using the team-based approach described earlier. A total of 1835 (46%) transfers were identified as unavoidable.  ***2) QI opportunities related to avoidable and unavoidable transfers.***  QI opportunities related to avoidable transfers were earlier detection of new signs/symptoms; discussions of resident/family preference; advance directive/hospice care; better communication about condition; and condition could have been managed in the NH. Three factors related to unavoidable transfers were bleeding, nausea/vomiting, and resident/family preference for hospitalization. |
| 35 | [Saliba et al. (2000)](#_ENREF_40) | USA | A structured implicit review (SIR) of medical records. Retrospective. | 458 residents transferred from the SNF to the ED or hospital. | SNF - ED/hospital | Structured implicit review (SIR) | ***1) Percentage of appropriate transfers.***  Assuming no directive limits care: two reviewers agreed that 36% of ED transfers and 40% of hospital admissions are inappropriate. They also agreed that 48% of ED and 49% of hospital admissions are appropriate. Considering advance directives: two reviewers agreed that 44% of ED transfers and 45% of hospital admissions are inappropriate. They also agreed that 41% of ED and 44% of hospital admissions are appropriate. |
| 36 | [Schwab et al. (2018)](#_ENREF_42) | France | Patient data from a monocentric, retrospective, matched, case-control study using the clinical data warehouseof a French university hospital. | 438 patients, who experienced an unscheduled readmission within 30 days after the index discharge. The control group consisted of elderly patients who had not had any unscheduled readmissions during the 30 days after the index discharge. | 1. Unplanned readmission (AED visit or readmission) or death during the 12-month follow-up period 2. Unplanned readmission to hospital or death within 30 days of discharge 3. 30 day potentially avoidable readmission 4. AED visit, hospital admission or nursing home admission at 30 and 120 days. | 1. The 80+ score 2. The LACE index 3. The HOSPITAL score 4. The TRS | ***1) Score results.***  Using a t-test, the means of the scores were compared between cases and controls (table 4). The 80+ score, the LACE index and the HOSPITAL score had p-values of 0.87, 0.24 and 0.60, respectively, meaning that there was no significant difference between cases and controls. In contrast, for the TRST, the mean score of the cases was significantly different from the mean score of the controls. |
| 37 | [Shams et al. (2015)](#_ENREF_44) | USA | Retrospective cohort study. | 5,600 eligible admissions. Inpatient administrative records gathered from four medical facilities were analysed. 7200 records that correspond to 2985 distinct adult patients. | 30 day avoidable readmissions | Potentially Avoidable Readmission (PAR) algorithm | ***1) 30-day avoidable readmission.***  PAR rate is found to be 11.77 %. |
| 38 | [Soria-Aledo et al. (2012)](#_ENREF_45) | Spain | A retrospective pre- and post-intervention study | 1450 histories (725 belonging to the control group and 725 to the intervention group). | Transition from emergencies, consultation, home or other centres to the hospital | AEP (Appropriateness Evaluation Protocol) Spanish version | ***1) Inappropriate admissions.***  Comparing control and intervention groups, inappropriateness of admission in the control group was 7.4% (54 patients), whereas in the intervention group it was significantly reduced to 3.2% (23 patients).  ***2) Inappropriate stays.***  Comparing control and intervention groups, the percentage of inappropriate stays was 24.6% (334 patients) in the control group and 10.4% (137 patients) in the intervention group.  ***3) Cost of inappropriateness.***  The cost of the days considered inappropriate in the study sample, taking into account the mean cost per patient, clinical service and day, was 147,044 euros in the control group and 66,462 euros in the intervention group. |
| 39 | [Stadler et al. (2019)](#_ENREF_46) | USA | Prospective cohort, pre/post study | 216 residents. 3 SNFs. | SNF- hospital/ED | RAFT (Reducing Avoidable Facility Transfers) model | ***1) ED transfers and hospitalizations for SNF residents overall, and for post-acute care (PAC) and long-term care (LTC) subgroups.***  Mean monthly ED transfers decreased from 24.8 (6.5) at baseline to 15.9 (3.0) post intervention, representing a 35.8% reduction. Mean monthly LTC ED transfers reduced from 11.1 (3.9) at baseline to 4.2 (2.3) post intervention, representing a 61.9% reduction. Mean monthly ED transfers of PAC patients did not change significantly post intervention. Mean monthly hospitalizations decreased by 30.5% from 15.8 (6.2) to 10.9 (3.7) post intervention. LTC hospitalizations decreased from a monthly average of 6.5 (2.9) at baseline to 2.4 (1.5) post intervention, a 62.4% reduction. PAC average monthly hospitalizations decreased slightly from 9.3 (4.8) at baseline to 8.5 (3.0) post intervention, representing an 8.1% reduction.  ***2) Advanced care planning status, hospital charges, and standard Minimum Data Set (MDS) quality metrics.***  Key characteristics that could potentially influence ED and hospital utilization were measured. These included reported MDS quality measures, staffing ratio and case mix. No significant changes in any of these factors during the intervention period when compared to previous years were observed. |
| 40 | [Stiell et al. (2017)](#_ENREF_48) | Canada | Prospective observational cohort study | 1100 patients, with shortness of breath due to acute heart failure, either admitted to the hospital or discharged from the ED. | ED admissions (but also discharge decisions) | Ottawa Heart Failure Risk Scale (OHFRS) | ***1) SAE (serious adverse events).***  The overall SAE rate was 15.5%, 19.4% for patients admitted, 10.2% for those discharged from the ED, and 17.4% for those with NT-proBNP values.  ***2) Physician accuracy in interpretation, acceptability to the physicians, and potential impact on disposition decisions.***  Risk category (i.e., low, medium, etc.) classification of the physicians to the criterion interpretation was compared. Overall agreement of 59.2% for the exact category and 94.7% for the exact category +- 1 was found. Issues identified were not completing the walk test, not having the NT-proBNP values available clinically, and too many criteria. In 11.9% of cases, physicians indicated that they would be “uncomfortable” or “very uncomfortable” in using OHFRS to make disposition decisions for that patient. Commonly expressed concerns were unavailability of NT-proBNP values and forgetting to arrange for a walk test. |
| 41 | [Stiell et al. (2018)](#_ENREF_47) | Canada | Prospective cohort study | 1415 patients, who presented to the emergency department with acute shortness of breath or respiratory distress caused by exacerbation of COPD and who might be considered well enough to be discharged by the attending physician. | ED admissions and discharges | The Ottawa COPD (chronic obstructive pulmonary disease) Risk Scale (OCRS) | ***1) Short-term serious outcomes.***  Among the 1415 participants who were enrolled (Table 2), there were 135 (9.5%) short-term serious outcomes, with higher rates in those admitted compared with those discharged from the emergency department (11.0% v. 8.3%, p < 0.01).  ***2) Physicians answering the following question, “How comfortable would you be using this scale to assist making a disposition decision for this patient?”***  On the 5-point scale of comfort in using OCRS, the physicians indicated that they would be uncomfortable or very uncomfortable in only 13.4% of cases. |
| 42 | [Subbe et al. (2001)](#_ENREF_49) | UK | Prospective cohort study | 673 medical emergency admissions. | Admission to HDU or ICU | Modified Early Warning Score (MEWS) | ***1) HDU and ICU admission, attendance of the cardiac arrest team at a cardiorespiratory emergency and death at 60 days.***  During follow-up, 7 patients were admitted to ICU, 23 to HDU, 4 were resuscitated by the cardiopulmonary arrest and 56 died. |
| 43 | [Teh and Janus (2018)](#_ENREF_52) | Australia | Prospective study | 781 patients. | 30 day hospital readmissions | Revised LACE index | ***1) Readmission rates for those who had revised LACE index scores of 8 and above, and those with scores below 8, and also between those who participated vs. those who chose not to participate in RAPT intervention.***  Median revised LACE index score for all admission episodes was 7 [IQR 4, 8], with 358 (41.0%) admission episodes classified as high risk of early readmission. Revised LACE index scores were equivalent for readmission vs. non-readmission episodes (7 [IQR 5, 8] vs. 7 [IQR 4, 8]), although there was a trend toward scores of 8 and above being more frequent among readmission vs. non- readmission episodes. Of the 358 admission episodes identified as high risk, 133 (37.2%) received RAPT intervention, and 53 (14.8%) formally declined. Among the 133 admission episodes that received RAPT intervention, there were 19 (14.3%) subsequent early readmissions and 114 (85.7%) were not readmitted. Early readmission rates were equivalent for those who received vs. those who did not receive RAPT intervention (14.3 vs. 14.7%). The readmission rate was 10.5% for those who received specialist clinic appointments, 17.8% for HARP referrals, and 20.0% for IRS referrals. Among those who declined RAPT intervention, the readmission rate was 22.6% for those who failed to attend or can- celled their clinic appointment and 16.7% for those who refused clinic during their inpatient stay. |
| 44 | [Uhlmann et al. (2017)](#_ENREF_53) | Switzerland | Retrospective study | 6729 hospital stays. | Potentially avoidable readmission (PARA) | 1. LACE index  2. HOSPITAL score  3. Risk prediction model for PARAs | ***1) PARA.***  777 stays were followed by a PARA; 5952 patients were not readmitted. |
| 45 | [Unroe et al. (2015)](#_ENREF_54) | USA | Prospective study | 4,035 long-stay nursing-home residents | Avoidable hospitalizations | Root cause analysis (INTERACT QI Acute care transfers (ACT) tool) | ***1) Avoidable hospitalizations.***  Of the 910 transfers that the RNs evaluated, 28% were judged to be avoidable, 57% were unavoidable, and 15% had no response coded, because information was missing or the RN could not reach a conclusion. |
| 46 | [Velasco Díaz et al. (2005)](#_ENREF_55) | Spain | Retrospective and descriptive study | Medical records of 622 patients admitted to medical services from the ED. | Unnecessary emergency admissions | Appropriateness evaluation protocol Spanish version (AEP) | ***1) Appropriateness of admissions.***  Of the 622 admissions reviewed, 63 (10.1%) were considered inappropriate.  ***2) Appropriateness of stays***.  Unnecessary admissions generated 78.2% of unnecessary stays, and the appropriate admissions generated 24.8% of unnecessary stays. |
| 47 | [Wright et al. (2014)](#_ENREF_56) | UK | Pre- and post-retrospective cohort study | Comparison of 5,416 emergency geriatric admissions in the 12 months preceding TREAT with the 5,370 emergency geriatric admissions in the 12 months following | Unplanned hospital admissions | Triage and Rapid Elderly Assessment Team (TREAT). (Includes Comprehensive Geriatric Assessment) | ***1) Same-day discharge rate as a percentage of admissions (an inverse measure of admission rate).***  After the introduction of TREAT, the percentage of admissions resulting in same-day discharges increased from 12.26 to 16.23% for TREAT-matching Admissions, but for the residual population fell from 15.01 to 9.77%.  ***2) LOS (Length of Stay).***  After the introduction of TREAT, the median LOS for TREAT-matching admissions reduced by 2 days, and mean LOS by 18.16%. For the residual admissions, the median was unchanged, and mean LOS reduced by 1.08%. For all Emergency Geriatric Admissions population, median LOS reduced by 1 day, and the mean LOS by 11.65%. |
| 48 | [Zerah et al. (2022)](#_ENREF_57) | Switzerland, The Netherlands, Belgium, Ireland | Retrospective study using data from the OPERAM (OPtimising thERapy to prevent Avoidable hospital admissions in Multimorbid older people) trial. | 832 patients, 4 medical centres from 4 countries. | Drug related hospital admissions | 1) Standardised chart review  2) Standardised chart review with revised trigger tool | ***1) ADE (adverse drug event), DRA (drug related admission), preventable DRAs.***  673 hospitalizations (55%) had at least one identified ADE and 518 were adjudicated as DRAs (42%; Figure 1). Of the 518 DRAs identified, 219 (42%) could be considered as preventable (due in whole or in part to overuse [N=55, 11%], underuse [N=135, 26%] and/or misuse [N=45, 9%]). |
| 49 | [Zúñiga et al. (2022)](#_ENREF_58) | Switzerland | Multicenter nonrandomized stepped-wedge design within a hybrid type-2 effectiveness study | 11 NHs in German-speaking Switzerland. 942 residents. | NH - hospital/ED | INTERCARE nurse-led model (Includes Comprehensive Geriatric Assessment) | ***1) Unplanned hospitalizations.***  Raw rates for unplanned transfers per 10000 resident days were 0.41 for the three baseline months and subsequently 0.84 (intervention start=T1), 0.85 (3 months after T1), 0.64 (6 months after T1), 0.79 (9 months after T1), and 0.42 (12 months after T1) unplanned transfers/10000 resident days per quarterly period after baseline. During the 3-month baseline and 18-month intervention study periods, 367 hospital transfers occurred, of which 303 transfers (82.6%) were unplanned (primary outcome) and 64 transfers (17.4%) planned. At the resident level, 225 residents (23.9%) were transferred to a hospital at least once for an unplanned reason. |

Abdoulhadi, D., Chevalet, P., Moret, L., Fix, M. H., Gégu, M., Jaulin, P., Berrut, G., & de Decker, L. (2015). [Appropriateness of direct admissions to acute care geriatric unit for nursing home patients: an adaptation of the AEPf GRID]. *Geriatr Psychol Neuropsychiatr Vieil*, *13*(1), 15-21. <https://doi.org/10.1684/pnv.2015.0522>

Aliberti, S., Ramirez, J., Cosentini, R., Brambilla, A. M., Zanaboni, A. M., Rossetti, V., Tarsia, P., Peyrani, P., Piffer, F., & Blasi, F. (2011). Low CURB-65 is of limited value in deciding discharge of patients with community-acquired pneumonia. *Respir Med*, *105*(11), 1732-1738. <https://doi.org/10.1016/j.rmed.2011.07.006>

Almeida, A., Serrasqueiro, Z., & Rogerio, A. (2006). [Review of the utilization of a Portuguese public hospital]. *Acta Med Port*, *19*(5), 381-385.

Arendts, G., Etherton-Beer, C., Jones, R., Bullow, K., MacDonald, E., Dumas, S., Parker, D., Hutton, M., Burrows, S., Brown, S. G., & Almeida, O. P. (2015). Use of a risk nomogram to predict emergency department reattendance in older people after discharge: a validation study. *Intern Emerg Med*, *10*(4), 481-487. <https://doi.org/10.1007/s11739-015-1219-3>

Attena, F., Agozzino, E., Troisi, M. R., Granito, C., & Del Prete, U. (2001). Appropriateness of admission and hospitalization days in a specialist hospital. *Ann Ig*, *13*(2), 121-127.

Aubert, C. E., Schnipper, J. L., Williams, M. V., Robinson, E. J., Zimlichman, E., Vasilevskis, E. E., Kripalani, S., Metlay, J. P., Wallington, T., Fletcher, G. S., Auerbach, A. D., Aujesky, D., & J, D. D. (2017). Simplification of the HOSPITAL score for predicting 30-day readmissions. *BMJ Qual Saf*, *26*(10), 799-805. <https://doi.org/10.1136/bmjqs-2016-006239>

Baig, M., Zhang, E., Robinson, R., Ullah, E., & Whitakker, R. (2018). Evaluation of Patients at Risk of Hospital Readmission (PARR) and LACE Risk Score for New Zealand Context...Health Informatics Conference, Sydney Australia, 2018. *Studies in Health Technology & Informatics*, *252*, 21-26. <https://doi.org/10.3233/978-1-61499-890-7-21>

Baré, M. L., Prat, A., Lledo, L., Asenjo, M. A., & Salleras, L. (1995). Appropriateness of admissions and hospitalization days in an acute-care teaching hospital. *Rev Epidemiol Sante Publique*, *43*(4), 328-336.

Bermejo Higuera Jc, C. M. R. D.-A. H. E. M. A. C. V. D. M. (2010). Derivaciones al Servicio de Urgencias del hospital en una población de ancianos residentes. Estudio retrospectivo sobre sus causas y adecuación. [Hospital transfers from a population of elderly residents. A retrospective study about the causes and suitability]. *Gerokomos.*, *21*(3), 114-117.

Burke, R. E., Schnipper, J. L., Williams, M. V., Robinson, E. J., Vasilevskis, E. E., Kripalani, S., Metlay, J. P., Fletcher, G. S., Auerbach, A. D., & Donzé, J. D. (2017). The HOSPITAL Score Predicts Potentially Preventable 30-Day Readmissions in Conditions Targeted by the Hospital Readmissions Reduction Program. *Med Care*, *55*(3), 285-290. <https://doi.org/10.1097/mlr.0000000000000665>

Codde, J., Frankel, J., Arendts, G., & Babich, P. (2010). Quantification of the proportion of transfers from residential aged care facilities to the emergency department that could be avoided through improved primary care services. *Australas J Ageing*, *29*(4), 167-171. <https://doi.org/10.1111/j.1741-6612.2010.00496.x>

Davido, A., Nicoulet, I., Levy, A., & Lang, T. (1991). Appropriateness of admission in an emergency department: reliability of assessment and causes of failure. *Qual Assur Health Care*, *3*(4), 227-234. <https://doi.org/10.1093/intqhc/3.4.227>

De Giorgi, A., Boari, B., Tiseo, R., López-Soto, P. J., Signani, F., Gallerani, M., Manfredini, R., & Fabbian, F. (2016). Hospital readmissions to internal medicine departments: a higher risk for females? *Eur Rev Med Pharmacol Sci*, *20*(21), 4557-4564.

Donzé, J., Aujesky, D., Williams, D., & Schnipper, J. L. (2013). Potentially avoidable 30-day hospital readmissions in medical patients: derivation and validation of a prediction model. *JAMA Intern Med*, *173*(8), 632-638. <https://doi.org/10.1001/jamainternmed.2013.3023>

Donzé, J. D., Williams, M. V., Robinson, E. J., Zimlichman, E., Aujesky, D., Vasilevskis, E. E., Kripalani, S., Metlay, J. P., Wallington, T., Fletcher, G. S., Auerbach, A. D., & Schnipper, J. L. (2016). International Validity of the HOSPITAL Score to Predict 30-Day Potentially Avoidable Hospital Readmissions. *JAMA Intern Med*, *176*(4), 496-502. <https://doi.org/10.1001/jamainternmed.2015.8462>

Downs, M., Blighe, A., Carpenter, R., Feast, A., Froggatt, K., Gordon, S., Hunter, R., Jones, L., Lago, N., McCormack, B., Marston, L., Nurock, S., Panca, M., Permain, H., Powell, C., Rait, G., Robinson, L., Woodward-Carlton, B., Wood, J., Young, J., et al. (2021). A complex intervention to reduce avoidable hospital admissions in nursing homes: a research programme including the BHiRCH-NH pilot cluster RCT. *Programme Grants for Applied Research*. <https://doi.org/10.3310/pgfar09020>

Duflos, C., Antoun, S., Loirat, P., DiPalma, M., & Minvielle, E. (2017). Identification of appropriate and potentially avoidable emergency department referrals in a tertiary cancer care center. *Support Care Cancer*, *25*(8), 2377-2385. <https://doi.org/10.1007/s00520-017-3611-1>

Fine, M. J., Auble, T. E., Yealy, D. M., Hanusa, B. H., Weissfeld, L. A., Singer, D. E., Coley, C. M., Marrie, T. J., & Kapoor, W. N. (1997). A prediction rule to identify low-risk patients with community-acquired pneumonia. *N Engl J Med*, *336*(4), 243-250. <https://doi.org/10.1056/nejm199701233360402>

Gozalo, P., Teno, J. M., Mitchell, S. L., Skinner, J., Bynum, J., Tyler, D., & Mor, V. (2011). End-of-Life Transitions among Nursing Home Residents with Cognitive Issues. *New England Journal of Medicine*, *365*(13), 1212-1221. <https://doi.org/10.1056/NEJMsa1100347>

Higi, L., Lisibach, A., Beeler, P. E., Lutters, M., Blanc, A. L., Burden, A. M., & Stämpfli, D. (2021). External validation of the PAR-Risk Score to assess potentially avoidable hospital readmission risk in internal medicine patients. *PLoS One*, *16*(11), e0259864. <https://doi.org/10.1371/journal.pone.0259864>

Horey, D. E., Street, A. F., & Sands, A. F. (2012). Acceptability and feasibility of end-of-life care pathways in Australian residential aged care facilities. *Med J Aust*, *197*(2), 106-109. <https://doi.org/10.5694/mja11.11518>

Huckfeldt, P. J., Kane, R. L., Yang, Z., Engstrom, G., Tappen, R., Rojido, C., Newman, D., Reyes, B., & Ouslander, J. G. (2018). Degree of Implementation of the Interventions to Reduce Acute Care Transfers (INTERACT) Quality Improvement Program Associated with Number of Hospitalizations. *J Am Geriatr Soc*, *66*(9), 1830-1837. <https://doi.org/10.1111/jgs.15476>

Hullick, C., Conway, J., Higgins, I., Hewitt, J., Dilworth, S., Holliday, E., & Attia, J. (2016). Emergency department transfers and hospital admissions from residential aged care facilities: a controlled pre-post design study. *BMC Geriatr*, *16*, 102. <https://doi.org/10.1186/s12877-016-0279-1>

Hullick, C. J., Hall, A. E., Conway, J. F., Hewitt, J. M., Darcy, L. F., Barker, R. T., Oldmeadow, C., & Attia, J. R. (2021). Reducing Hospital Transfers from Aged Care Facilities: A Large-Scale Stepped Wedge Evaluation. *J Am Geriatr Soc*, *69*(1), 201-209. <https://doi.org/10.1111/jgs.16890>

Inzitari, M., Gual, N., Roig, T., Colprim, D., Pérez-Bocanegra, C., San-José, A., & Jimenez, X. (2015). Geriatric Screening Tools to Select Older Adults Susceptible for Direct Transfer From the Emergency Department to Subacute Intermediate-Care Hospitalization. *J Am Med Dir Assoc*, *16*(10), 837-841. <https://doi.org/10.1016/j.jamda.2015.04.009>

Johnston, J. J., Longman, J. M., Ewald, D. P., Rolfe, M. I., Diez Alvarez, S., Gilliland, A. H. B., Chung, S. C., Das, S. K., King, J. M., & Passey, M. E. (2020). Validity of a tool designed to assess the preventability of potentially preventable hospitalizations for chronic conditions. *Fam Pract*, *37*(3), 390-394. <https://doi.org/10.1093/fampra/cmz086>

Kane, R. L., Huckfeldt, P., Tappen, R., Engstrom, G., Rojido, C., Newman, D., Zhiyou, Y., Ouslander, J. G., & Yang, Z. (2017). Effects of an Intervention to Reduce Hospitalizations From Nursing Homes: A Randomized Implementation Trial of the INTERACT Program. *JAMA Internal Medicine*, *177*(9), 1257-1264. <https://doi.org/10.1001/jamainternmed.2017.2657>

Karmakar, G., & Wilsher, M. (2010). Use of the 'CURB 65' score in hospital practice. *Intern Med J*, *40*(12), 828-832. <https://doi.org/10.1111/j.1445-5994.2009.02062.x>

Lamb, G., Tappen, R., Diaz, S., Herndon, L., & Ouslander, J. G. (2011). Avoidability of hospital transfers of nursing home residents: perspectives of frontline staff. *J Am Geriatr Soc*, *59*(9), 1665-1672. <https://doi.org/10.1111/j.1532-5415.2011.03556.x>

Lázaro Cebas, A., Caro Teller, J. M., García Muñoz, C., González Gómez, C., Ferrari Piquero, J. M., Lumbreras Bermejo, C., Romero Garrido, J. A., & Benedí González, J. (2022). Intervention by a clinical pharmacist carried out at discharge of elderly patients admitted to the internal medicine department: influence on readmissions and costs. *BMC Health Serv Res*, *22*(1), 167. <https://doi.org/10.1186/s12913-022-07582-6>

Migliorati, P. L., Boccoli, E., Bracci, L. S., Sestini, P., & Melani, A. S. (2006). A survey on hospitalised community-acquired pneumonia in Italy. *Monaldi Arch Chest Dis*, *65*(2), 82-88. <https://doi.org/10.4081/monaldi.2006.569>

Oddone, E. Z., Weinberger, M., Horner, M., Mengel, C., Goldstein, F., Ginier, P., Smith, D., Huey, J., Farber, N. J., Asch, D. A., Loo, L., Mack, E., Hurder, A. G., Henderson, W., & Feussner, J. R. (1996). Classifying general medicine readmissions. Are they preventable? Veterans Affairs Cooperative Studies in Health Services Group on Primary Care and Hospital Readmissions. *J Gen Intern Med*, *11*(10), 597-607. <https://doi.org/10.1007/bf02599027>

Ong, A. C., Sabanathan, K., Potter, J. F., & Myint, P. K. (2011). High mortality of older patients admitted to hospital from care homes and insight into potential interventions to reduce hospital admissions from care homes: the Norfolk experience. *Arch Gerontol Geriatr*, *53*(3), 316-319. <https://doi.org/10.1016/j.archger.2010.12.004>

Ouslander, J. G., Bonner, A., Herndon, L., & Shutes, J. (2014). The Interventions to Reduce Acute Care Transfers (INTERACT) quality improvement program: an overview for medical directors and primary care clinicians in long term care. *J Am Med Dir Assoc*, *15*(3), 162-170. <https://doi.org/10.1016/j.jamda.2013.12.005>

Ouslander, J. G., Lamb, G., Tappen, R., Herndon, L., Diaz, S., Roos, B. A., Grabowski, D. C., & Bonner, A. (2011). Interventions to reduce hospitalizations from nursing homes: evaluation of the INTERACT II collaborative quality improvement project. *J Am Geriatr Soc*, *59*(4), 745-753. <https://doi.org/10.1111/j.1532-5415.2011.03333.x>

Ouslander, J. G., Perloe, M., Givens, J. H., Kluge, L., Rutland, T., & Lamb, G. (2009). Reducing potentially avoidable hospitalizations of nursing home residents: results of a pilot quality improvement project. *J Am Med Dir Assoc*, *10*(9), 644-652. <https://doi.org/10.1016/j.jamda.2009.07.001>

Patel, R., Clancy, R., Crowther, E., Vannahme, M., & Pullyblank, A. (2014). A rectal bleeding algorithm can successfully reduce emergency admissions. *Colorectal Dis*, *16*(5), 377-381. <https://doi.org/10.1111/codi.12524>

Pérès, K., Rainfray, M., Perrié, N., Emeriau, J. P., Chêne, G., & Barberger-Gateau, P. (2002). [Incidence, risk factors and adequation of early readmission among the elderly]. *Rev Epidemiol Sante Publique*, *50*(2), 109-119.

Popejoy, L. L., Vogelsmeier, A. A., Alexander, G. L., Galambos, C. M., Crecelius, C. A., Ge, B., Flesner, M., Canada, K., & Rantz, M. (2019). Analyzing Hospital Transfers Using INTERACT Acute Care Transfer Tools: Lessons from MOQI. *J Am Geriatr Soc*, *67*(9), 1953-1959. <https://doi.org/10.1111/jgs.15996>

Saliba, D., Kington, R., Buchanan, J., Bell, R., Wang, M., Lee, M., Herbst, M., Lee, D., Sur, D., & Rubenstein, L. (2000). Appropriateness of the decision to transfer nursing facility residents to the hospital. *J Am Geriatr Soc*, *48*(2), 154-163. <https://doi.org/10.1111/j.1532-5415.2000.tb03906.x>

Sampson, E. L., Feast, A., Blighe, A., Froggatt, K., Hunter, R., Marston, L., McCormack, B., Nurock, S., Panca, M., Powell, C., Rait, G., Robinson, L., Woodward-Carlton, B., Young, J., & Downs, M. (2020). Pilot cluster randomised trial of an evidence-based intervention to reduce avoidable hospital admissions in nursing home residents (Better Health in Residents of Care Homes with Nursing-BHiRCH-NH Study). *BMJ Open*, *10*(12), e040732. <https://doi.org/10.1136/bmjopen-2020-040732>

Schwab, C., Le Moigne, A., Fernandez, C., Durieux, P., Sabatier, B., & Korb-Savoldelli, V. (2018). External validation of the 80+ score and comparison with three clinical scores identifying patients at least 75 years old at risk of unplanned readmission within 30 days after discharge. *Swiss Med Wkly*, *148*, w14624. <https://doi.org/10.4414/smw.2018.14624>

Selker, H. P., Beshansky, J. R., Griffith, J. L., Aufderheide, T. P., Ballin, D. S., Bernard, S. A., Crespo, S. G., Feldman, J. A., Fish, S. S., Gibler, W. B., Kiez, D. A., McNutt, R. A., Moulton, A. W., Ornato, J. P., Podrid, P. J., Pope, J. H., Salem, D. N., Sayre, M. R., Woolard, R. H., & Selker, H. P. (1998). Use of the acute cardiac ischemia time-insensitive predictive instrument (ACI-TIPI) to assist with triage of patients with chest pain or other symptoms suggestive of acute cardiac ischemia. A multicenter, controlled clinical trial. *Annals of Internal Medicine*, *129*(11), 845-855. <https://doi.org/10.7326/0003-4819-129-11_part_1-199812010-00002>

Shams, I., Ajorlou, S., & Yang, K. (2015). A predictive analytics approach to reducing 30-day avoidable readmissions among patients with heart failure, acute myocardial infarction, pneumonia, or COPD. *Health Care Manag Sci*, *18*(1), 19-34. <https://doi.org/10.1007/s10729-014-9278-y>

Soria-Aledo, V., Carrillo-Alcaraz, A., Flores-Pastor, B., Moreno-Egea, A., Carrasco-Prats, M., & Aguayo-Albasini, J. L. (2012). Reduction in inappropriate hospital use based on analysis of the causes. *BMC Health Serv Res*, *12*, 361. <https://doi.org/10.1186/1472-6963-12-361>

Stadler, D. S., Oliver, B. J., Raymond, J. G., Routzhan, G. F., Flaherty, E. A., Stahl, J. E., Batsis, J. A., & Bartels, S. J. (2019). Reducing Avoidable Facility Transfers (RAFT): Outcomes of a Team Model to Minimize Unwarranted Emergency Care at Skilled Nursing Facilities. *J Am Med Dir Assoc*, *20*(8), 929-934. <https://doi.org/10.1016/j.jamda.2019.03.010>

Stiell, I. G., Perry, J. J., Clement, C. M., Brison, R. J., Rowe, B. H., Aaron, S. D., McRae, A. D., Borgundvaag, B., Calder, L. A., Forster, A. J., Brinkhurst, J., & Wells, G. A. (2018). Clinical validation of a risk scale for serious outcomes among patients with chronic obstructive pulmonary disease managed in the emergency department. *Cmaj*, *190*(48), E1406-e1413. <https://doi.org/10.1503/cmaj.180232>

Stiell, I. G., Perry, J. J., Clement, C. M., Brison, R. J., Rowe, B. H., Aaron, S. D., McRae, A. D., Borgundvaag, B., Calder, L. A., Forster, A. J., Wells, G. A., & Runyon, M. S. (2017). Prospective and Explicit Clinical Validation of the Ottawa Heart Failure Risk Scale, With and Without Use of Quantitative NT-pro BNP. *Academic Emergency Medicine*, *24*(3), 316-327. <https://doi.org/10.1111/acem.13141>

Subbe, C. P., Kruger, M., Rutherford, P., & Gemmel, L. (2001). Validation of a modified Early Warning Score in medical admissions. *QJM: An International Journal of Medicine*, *94*(10), 521-526. <https://doi.org/10.1093/qjmed/94.10.521>

Tappen, R. M., Newman, D., Huckfeldt, P., Yang, Z., Engstrom, G., Wolf, D. G., Shutes, J., Rojido, C., & Ouslander, J. G. (2018). Evaluation of Nursing Facility Resident Safety During Implementation of the INTERACT Quality Improvement Program. *J Am Med Dir Assoc*, *19*(10), 907-913.e901. <https://doi.org/10.1016/j.jamda.2018.06.017>

Tappen, R. M., Worch, S. M., Newman, D. O., & Hain, D. (2020). Evaluation of a Novel Decision Guide "Go to the Hospital or Stay Here?" for Nursing Home Residents and Families: A Randomized Trial. *Res Gerontol Nurs*, *13*(6), 309-319. <https://doi.org/10.3928/19404921-20201002-01>

Teh, R., & Janus, E. (2018). Identifying and targeting patients with predicted 30-day hospital readmissions using the revised LACE index score and early postdischarge intervention. *Int J Evid Based Healthc*, *16*(3), 174-181. <https://doi.org/10.1097/xeb.0000000000000142>

Uhlmann, M., Lécureux, E., Griesser, A. C., Duong, H. D., & Lamy, O. (2017). Prediction of potentially avoidable readmission risk in a division of general internal medicine. *Swiss Med Wkly*, *147*, w14470. <https://doi.org/10.4414/smw.2017.14470>

Unroe, K. T., Nazir, A., Holtz, L. R., Maurer, H., Miller, E., Hickman, S. E., La Mantia, M. A., Bennett, M., Arling, G., & Sachs, G. A. (2015). The Optimizing Patient Transfers, Impacting Medical Quality, andImproving Symptoms:Transforming Institutional Care approach: preliminary data from the implementation of a Centers for Medicare and Medicaid Services nursing facility demonstration project. *J Am Geriatr Soc*, *63*(1), 165-169. <https://doi.org/10.1111/jgs.13141>

Velasco Díaz, L., García Ríos, S., Oterino de la Fuente, D., Suárez García, F., Diego Roza, S., & Fernández Alonso, R. (2005). [Impact on hospital days of care due to unnecessary emergency admissions]. *Rev Esp Salud Publica*, *79*(5), 541-549. <https://doi.org/10.1590/s1135-57272005000500004>

Wright, P. N., Tan, G., Iliffe, S., & Lee, D. (2014). The impact of a new emergency admission avoidance system for older people on length of stay and same-day discharges. *Age Ageing*, *43*(1), 116-121. <https://doi.org/10.1093/ageing/aft086>

Zerah, L., Henrard, S., Thevelin, S., Feller, M., Meyer-Masseti, C., Knol, W., Wilting, I., O'Mahony, D., Crowley, E., Dalleur, O., & Spinewine, A. (2022). Performance of a trigger tool for detecting drug-related hospital admissions in older people: analysis from the OPERAM trial. *Age & Ageing*, *51*(1), 1-13. <https://doi.org/10.1093/ageing/afab196>

Zúñiga, F., Guerbaai, R.-A., de Geest, S., Popejoy, L. L., Bartakova, J., Denhaerynck, K., Trutschel, D., Basinska, K., Nicca, D., Kressig, R. W., Zeller, A., Wellens, N. I. H., de Pietro, C., Desmedt, M., Serdaly, C., & Simon, M. (2022). Positive effect of the INTERCARE nurse-led model on reducing nursing home transfers: A nonrandomized stepped-wedge design. *Journal of the American Geriatrics Society*, *70*(5), 1546-1557. <https://doi.org/https://doi.org/10.1111/jgs.17677>
