# Supplementary material for: Assessment tools addressing avoidable care transitions in older adults: a systematic literature review
Source: Eur Geriatr Med. 2024 Nov 29;15(6):1587–601. doi: 10.1007/s41999-024-01106-7 (PMC11632047; doi:10.1007/s41999-024-01106-7)
Supplement: Supplementary file 3 — Supplementary file3 (DOCX 280 KB) [file 41999_2024_1106_MOESM3_ESM.docx]

**Supplementary file 3: Characteristics of the tools**

**Summary**

The supplementary file is structured into 36 sections with a total of 48 tables. Each section represents a family of tools and tables represent a particular assessment tool. For example, section 1 represents a family of INTERACT tools, with 4 tables representing its variations.

**List of abbreviations**

Y: Yes

N: No

NI: No Information

1. **INTERACT tools and their variations**

**Table 1. INTERACT tools (with focus on care paths only)**

| **Tool name (reported in (**[**1-5**](#_ENREF_1)**))** | | |
| --- | --- | --- |
| **INTERACT** tools with focus on decision support tools. | | |
| **Concept/components covered** | | |
| **Decision support tools** **(include care paths for 10 conditions)** for Home health care (INTERACT version 1.0 tools), Assisted living (INTERACT version 2.0 tools), Skilled nursing (INTERACT version 4.5 tools) :  Acute mental status change, Change in behaviour: evaluation of medical causes of new or worsening behavioural symptoms, Dehydration, Fever, Gastrointestinal symptoms, Shortness of breath, Symptoms of congestive heart failure, Symptoms of lower respiratory infection, Symptoms of urinary tract infection, Fall. ([6](#_ENREF_6)). | | |
| **What the tool measures/does** | | |
| Determine which residents could be safely managed in the nursing home. ([1-5](#_ENREF_1)) ([6](#_ENREF_6)). | | |
| **Objectivity** | | |
| **Process** | Y. Self explaining ([6](#_ENREF_6)). | |
| **Evaluation** | Y. For example signs of increased confusion, new or worsened memory loss, unrelieved pain, difficult or laboured breathing that is out of proportion to the patient’s level of physical activity, depending on a care path ([6](#_ENREF_6)). | |
| **Interpretation** | Y. Care paths are in the form of flow charts. Depending on situation/input information there is clear guidance on how to act next ([6](#_ENREF_6)). | |
| **Reliability** | | |
| **Inter-rater** | NI | |
| **Intra-rater** | NI | |
| **Validity** | | |
| **Convergent** | NI | |
| **Costs** | | |
| **Time to completion** | NI | |
| **Specific input data required** | Y. Depending on a care path, may require some specific data. For example X-ray, blood work, ECG, stool specimen for occult blood ([6](#_ENREF_6)). | |
| **Specific training required** | Y. INTERACT as a whole may require specific training and appropriate implementation in NHs. Interact website also includes an implementation guide, implementation check-list to assist NHs in getting started and monitor implementation process. Web site also offers a “Contact Us” feature for questions to be answered by the INTERACT team ([4](#_ENREF_4)).  For website, refer to: ([6](#_ENREF_6)).  Each participating NH appointed a team responsible for attending the learning sessions (relates to the whole intervention). ([5](#_ENREF_5)) | |
| **Who completed the tool?** | | |
| Care paths for home health care: all home health licensed staff ([6](#_ENREF_6)).  Care paths for assisted living: all assisted living licensed nursing staff and primary care clinicians ([6](#_ENREF_6)).  Care paths for skilled nursing: all skilled nursing facility/nursing facility licensed nursing staff and primary care clinicians ([6](#_ENREF_6)). | | |
| **When/where/how tool was/can be completed? (for example: at discharge/at admission, in hospital/in nursing home, retrospective/prospective)** | | |
| In NH, when a resident is evaluated by a nurse, after change in resident´s status was noted ([6](#_ENREF_6)). Likely prospective use. | | |
| **Language** | | **Tool can be seen/accessed in:** |
| English ([6](#_ENREF_6)) | | Website with INTERACT tools: ([6](#_ENREF_6)) |

**Table 2. QI review tool (from project INTERACT II)**

| **Tool name (reported in (**[**7**](#_ENREF_7)**))** | |
| --- | --- |
| **Quality improvement (QI) review tool (from project INTERACT II)** | |
| **Concept/components covered** | |
| Consists of four sections: resident information; hospital transfer information, including symptoms or change in condition that precipitated the transfer; actions taken by staff before the transfer; and analysis of factors that may have influenced the transfer decision and rating of the transfer as avoidable, possibly avoidable, or not avoidable. ([7](#_ENREF_7)) | |
| **What the tool measures/does** | |
| Assists NH staff in understanding the reasons for the transfer and identifies opportunities to improve identification and management of changes in resident status and reduce acute care transfers. ([7](#_ENREF_7)) | |
| **Objectivity** | |
| **Process** | NI |
| **Evaluation** | NI |
| **Interpretation** | NI |
| **Reliability** | |
| **Inter-rater** | Interrater reliability was calculated for a subset of 50 forms. Percentage raw agreement of all three raters was 78%. Agreement between each set of two raters ranged from 78% to 88%. ([7](#_ENREF_7)) |
| **Intra-rater** | NI |
| **Validity** | |
| **Convergent** | NI |
| **Costs** | |
| **Time to completion** | NI |
| **Specific input data required** | NI |
| **Specific training required** | Y. Initial collaborative calls focused on training and logistics associated with implementing INTERACT II tools ([7](#_ENREF_7)). |
| **Who completed the tool?** | |
| Registered nurses, licensed practical nurse, nurse manager, nurse educator, social workers ([7](#_ENREF_7)). | |
| **When/where/how tool was/can be completed? (for example: at discharge/at admission, in hospital/in nursing home, retrospective/prospective)** | |
| In NH, QI was applied retrospectively on every hospital transfer ([7](#_ENREF_7)). | |
| **Language** | **Tool can be seen/accessed in:** |
| Likely to be in English | Tool not found. |

**Table 3. INTERACT II tools (with focus on care paths only)**

| **Tool name (reported in (**[**8**](#_ENREF_8)**))** | |
| --- | --- |
| **INTERACT II** tools with focus on care paths (subset of tools from INTERACT II). | |
| **Concept/components covered** | |
| Care paths for Mental status change, Fever, Symptoms of lower respiratory infection, Symptoms of congestive heart failure, Symptoms of urinary tract infection, Dehydration. ([8](#_ENREF_8)) | |
| **What the tool measures/does** | |
| Determine which residents could be safely managed in the nursing home. ([8](#_ENREF_8)) | |
| **Objectivity** | |
| **Process** | NI |
| **Evaluation** | NI |
| **Interpretation** | NI |
| **Reliability** | |
| **Inter-rater** | NI |
| **Intra-rater** | NI |
| **Validity** | |
| **Convergent** | NI |
| **Costs** | |
| **Time to completion** | NI |
| **Specific input data required** | NI |
| **Specific training required** | Y. NH staff was educated prior to intervention ([8](#_ENREF_8)). |
| **Who completed the tool?** | |
| NH staff ([8](#_ENREF_8)). | |
| **When/where/how tool was/can be completed? (for example: at discharge/at admission, in hospital/in nursing home, retrospective/prospective)** | |
| In NH ([8](#_ENREF_8)). Likely prospective use. | |
| **Language** | **Tool can be seen/accessed in:** |
| Likely to be in English | Tool not found. |

**Table 4. Root cause analysis (INTERACT QI Acute care transfers (ACT) tool)**

| **Tool name (reported in (**[**9**](#_ENREF_9)**,** [**10**](#_ENREF_10)**))** | |
| --- | --- |
| **Root cause analysis (INTERACT QI Acute care transfers (ACT) tool)** | |
| **Concept/components covered** | |
| Broad ACT categories include (1) Resident characteristics and risk factors for  hospitalization; (2) acute change in condition and other non-clinical factors that contributed to the transfer; (3) action(s) taken to evaluate and manage the change in condition before transfer; (4) description of the hospital transfer; and (5) opportunities for improvement. ([9](#_ENREF_9)) | |
| **What the tool measures/does** | |
| Describes common clinical and non-clinical factors that help clinical staff understand reasons for the transfer and for process improvement  considerations to avoid future transfers. ([9](#_ENREF_9)) | |
| **Objectivity** | |
| **Process** | NI  However, described the ACT QI procedures:  “(1) monthly review of all ACTs by a team composed of the APRN, project coordinator, and APRN supervisor; (2) identification of resident and NH factors contributing to the transfer; and (3) agreement by the team on the question “Was the transfer potentially preventable (avoidable)?” using the QI technique “Five Why’s.” This technique is an iterative interrogative approach to exploring cause and effect and was used by the team to assure root causes of transfers were considered when establishing agreement on which transfer were deemed avoidable vs unavoidable.” ([9](#_ENREF_9)) |
| **Evaluation** | NI |
| **Interpretation** | Y. “Agreement by the team on the question “Was the transfer potentially preventable (avoidable)?” using the QI technique “Five Why’s.” ([9](#_ENREF_9)) |
| **Reliability** | |
| **Inter-rater** | NI |
| **Intra-rater** | NI |
| **Validity** | |
| **Convergent** | NI |
| **Costs** | |
| **Time to completion** | Slightly longer than 30 min. ([10](#_ENREF_10)) |
| **Specific input data required** | NI |
| **Specific training required** | RNs (registered nurses) completed facility staff trainings on several INTERACT tools, including root cause analysis of hospital transfers. ([10](#_ENREF_10)) |
| **Who completed the tool?** | |
| Advanced practice registered nurses. ([9](#_ENREF_9)) | |
| **When/where/how tool was/can be completed? (for example: at discharge/at admission, in hospital/in nursing home, retrospective/prospective)** | |
| In NH, retrospectively applied with regard to hospital transfers. ([9](#_ENREF_9))  After discharge from hospital back to nursing home / nursing home / likely retrospective. ([10](#_ENREF_10)) | |
| **Language** | **Tool can be seen/accessed in:** |
| Likely to be in English | Tool not found. |

1. **ACE service model / The Aged Care Emergency (ACE) program**

**Table 5. ACE model (with focus on evidence-based algorithms only)**

| **Tool name (reported in (**[**11**](#_ENREF_11)**,** [**12**](#_ENREF_12)**))** | |
| --- | --- |
| **ACE service model** / The Aged Care Emergency (ACE) program with focus on evidence-based algorithms (for clinical use). | |
| **Concept/components covered** | |
| Some of the algorithms for the following conditions also include further related sub-sections: Allergic Reactions/Anaphylaxis, Assaults, Cardiology, Cellulitis, Dental and Oral Health, Diabetes, Falls, Gastroenterology, Neurology, Nosebleeds (Epistaxis), Pain, Palliative Care and Last Days of Life Care, Polypharmacy and high-risk medications in RACFs, Respiratory, Subcutaneous Fluid Administration, Urology, Wound Care. ([13](#_ENREF_13), [14](#_ENREF_14)) | |
| **What the tool measures/does** | |
| Determine which residents could be safely managed in the nursing home. ([11-14](#_ENREF_11)) | |
| **Objectivity** | |
| **Process** | Y. Self-explaining. |
| **Evaluation** | Y. Depending on algorithm. For example measuring pain level ([13](#_ENREF_13)), although it can be measured with other specifically designed tools, pain measurement by nature may still be subjective to some extent. |
| **Interpretation** | Y. Algorithms frequently in the form of flow charts (some are in text form or table form). Depending on input information/situation, there is a guidance on how to act next ([13](#_ENREF_13)). |
| **Reliability** | |
| **Inter-rater** | NI |
| **Intra-rater** | NI |
| **Validity** | |
| **Convergent** | NI |
| **Costs** | |
| **Time to completion** | NI |
| **Specific input data required** | Y. Depending on algorithm, may require specific data. For example test for blood glucose level (BGL), testing of urine for culture and sensitivity, imaging (CT head or MRI) ([13](#_ENREF_13)). |
| **Specific training required** | Y. May require some training. Education resources are also provided on the ACE website here: ([15](#_ENREF_15)). |
| **Who completed the tool?** | |
| The manual of algorithms guides and supports RACF staff to manage acutely unwell residents in situ and is used as a reference source by RNs, AINs, PCAs, GPs and ED staff ([12](#_ENREF_12)). | |
| **When/where/how tool was/can be completed? (for example: at discharge/at admission, in hospital/in nursing home, retrospective/prospective)** | |
| In the RACF. Algorithms were used to manage acutely unwell residents in situ ([12](#_ENREF_12)). Likely prospective use. | |
| **Language** | **Tool can be seen/accessed in:** |
| English ([13](#_ENREF_13)). | Algorithms can be accesses on a website (they are presented separately). Username is “aged” and password is “care”: ([13](#_ENREF_13)), or in the form of generic ACE manual, which contains all algorithms and other ACE model components: ([14](#_ENREF_14)). |

1. **A complex intervention (BHiRCH-NH) to reduce avoidable hospital admissions in nursing homes**

**Table 6. A complex intervention (BHiRCH-NH) to reduce avoidable hospital admissions in nursing homes (with focus on care pathway only)**

| **Tool name (reported in (**[**16**](#_ENREF_16)**,** [**17**](#_ENREF_17)**))** | |
| --- | --- |
| **A complex intervention (BHiRCH-NH) to reduce avoidable hospital admissions in nursing homes** with focus on care a pathway. | |
| **Concept/components covered** | |
| Care pathway is a clinical guidance and decision-support system for 4 ACSCs: dehydration, deterioration of congestive heart failure, lower respiratory tract infection, urinary tract infection. ([16](#_ENREF_16), [18](#_ENREF_18)) | |
| **What the tool measures/does** | |
| Determine which residents could be safely managed in the nursing home. ([16](#_ENREF_16), [18](#_ENREF_18)) | |
| **Objectivity** | |
| **Process** | Y. Self-explaining ([18](#_ENREF_18)). |
| **Evaluation** | Y. Some elements of a care pathway may seem subjective by nature. For example when checking for lower UTI symptoms (i.e. discomfort on passing urine, lower abdominal discomfort/pain) ([18](#_ENREF_18)). |
| **Interpretation** | N. Following the use of the care pathway, the nurse will make a clinical decision about the next course of action which will include one or more of the following actions: further general monitoring using S&W tool or direct monitoring for specific symptoms of the resident´s condition, initiate treatment in a care home, in case of potential diagnosis/immediate concern about resident´s condition communicate with primary care using SBAR tool ([18](#_ENREF_18)). |
| **Reliability** | |
| **Inter-rater** | NI |
| **Intra-rater** | NI |
| **Validity** | |
| **Convergent** | NI |
| **Costs** | |
| **Time to completion** | NI |
| **Specific input data required** | Y. May require specific data, for example testing for UTI, lower respiratory tract infection ([18](#_ENREF_18)). |
| **Specific training required** | Y. Some training may be required. It was reported that a workshop, introductory meetings and telephone coaching were provided. However, this training was on the whole intervention (care pathway is part of this intervention) ([16](#_ENREF_16)). |
| **Who completed the tool?** | |
| By a nurse ([18](#_ENREF_18)). | |
| **When/where/how tool was/can be completed? (for example: at discharge/at admission, in hospital/in nursing home, retrospective/prospective)** | |
| In NH. Applied when a change in resident´s condition was noted ([18](#_ENREF_18)). Likely prospective use. | |
| **Language** | **Tool can be seen/accessed in:** |
| English ([18](#_ENREF_18)) | Care pathway can be accessed in Appendix 12: ([18](#_ENREF_18)) |

1. **Novel Decision Guide "Go to the Hospital or Stay Here?"**

**Table 7. Novel Decision Guide "Go to the Hospital or Stay Here?"**

| **Tool name (reported in (**[**19**](#_ENREF_19)**))** | |
| --- | --- |
| **Novel Decision Guide "Go to the Hospital or Stay Here?"** | |
| **Concept/components covered** | |
| Change in condition, what to expect in different situations, how to get involved in a decision, pros and cons of being treated in a hospital or in the NH, FAQs, decision tree, what residents family and caregivers say and more. ([20](#_ENREF_20)) | |
| **What the tool measures/does** | |
| To guide residents, families, friends, caregivers on a decision-making whether to go to a hospital or stay in a nursing home. ([19](#_ENREF_19), [20](#_ENREF_20)) | |
| **Objectivity** | |
| **Process** | Y. Self-explaining ([20](#_ENREF_20)) |
| **Evaluation** | Y. Decision guide provides information in the form of text, frequently asked questions, and also provides a decision tree. A decision tree has some parts that may seem subjective in nature, for example assessing how sick a person is with 3 options: mild, moderate, very sick. ([20](#_ENREF_20)) |
| **Interpretation** | Y. When talking about a decision tree (which is part of the decision guide), it has a clear guidance on how to arrive at a certain conclusion depending on situation/input data. |
| **Reliability** | |
| **Inter-rater** | NI |
| **Intra-rater** | NI |
| **Validity** | |
| **Convergent** | NI |
| **Costs** | |
| **Time to completion** | NI |
| **Specific input data required** | Y. When talking about a decision tree (part of the decision guide), it may require some tests, but specific tests are not mentioned ([20](#_ENREF_20)) (we assume because the guide is for use by patients/caregivers, and not be used by care professionals). |
| **Specific training required** | Y. Member of the research team reviewed the contents of the Guide with participants and answered any questions that arose. ([19](#_ENREF_19))  Educational material & resources for residents/families and for care professionals are available here: ([20](#_ENREF_20)) |
| **Who completed the tool?** | |
| For NH residents, families, friends, caregivers | |
| **When/where/how tool was/can be completed? (for example: at discharge/at admission, in hospital/in nursing home, retrospective/prospective)** | |
| In NH, when an acute change in resident´s condition occurs ([20](#_ENREF_20)). Prospective use? | |
| **Language** | **Tool can be seen/accessed in:** |
| English, Spanish, French, Filipino, Creole, Chinese ([20](#_ENREF_20)) | Decision guide can be accessed from (in booklet or in trifold form): ([20](#_ENREF_20)) |

1. **ACI-TIPI acute cardiac ischemia time-insensitive predictive instrument**

**Table 8. ACI-TIPI acute cardiac ischemia time-insensitive predictive instrument**

| **Tool name (reported in (**[**21**](#_ENREF_21)**))** | |
| --- | --- |
| **ACI-TIPI acute cardiac ischemia time-insensitive predictive instrument** | |
| **Concept/components covered** | |
| Software based tool. | |
| **What the tool measures/does** | |
| Calculates probability of acute ischemia. | |
| **Objectivity** | |
| **Process** | Y. Sort of “software” tool. |
| **Evaluation** | N. Automatically computed by the electrocardiograph. |
| **Interpretation** | Y. On presentation to the emergency department, each patient's ACI-TIPI probability of acute ischemia was automatically computed by the electrocardiograph. During intervention periods, the probability was automatically printed on the electro-cardiogram header, with an indication that it was "to supplement, not replace physician judgment," along with the standard electrocardiogram interpretive header text. |
| **Reliability** | |
| **Inter-rater** | NI |
| **Intra-rater** | NI |
| **Validity** | |
| **Convergent** | Both manufacturers' ACI-TIPI electrocardiographs had ROC areas of 0.78. |
| **Costs** | |
| **Time to completion** | Instant (computed by the electrocardiograph) |
| **Specific input data required** | N. To acquire the ACI-TIPI probability in clinical use, the user enters the patient's age and sex and indicates whether chest or left arm pain is the primary symptom; the electrocardiograph then directly measures the wave-forms and computes and prints the probability of acute ischemia on the electrocardiogram header for the physician's immediate use. |
| **Specific training required** | NI |
| **Who completed the tool?** | |
| Physician. | |
| **When/where/how tool was/can be completed? (for example: at discharge/at admission, in hospital/in nursing home, retrospective/prospective)** | |
| In ED. On presentation of a patient to the emergency department. ([21](#_ENREF_21)) Likely prospective use. | |
| **Language** | **Tool can be seen/accessed in:** |
| English | Tool described in the study. ([21](#_ENREF_21)) |

1. **Appropriateness Evaluation Protocol (AEP) and its sub-types**

**Table 9. AEP (with focus on criteria of appropriateness of admission only)**

| **Tool name (reported in (**[**22**](#_ENREF_22)**))** | |
| --- | --- |
| **AEP** with focus on criteria of appropriateness of admission. | |
| **Concept/components covered** | |
| 18 criteria of appropriateness of hospital admission, of which 11 are related to severity of illness and patient condition, and 7 related to health care requirements or intensity of services. | |
| **What the tool measures/does** | |
| Appropriateness of hospital admissions. | |
| **Objectivity** | |
| **Process** | Y. Self-explaining. |
| **Evaluation** | Y. Has some parts, which may be subjective. For example, acute or progressive sensory, motor, circulatory or respiratory embarrassment sufficient to incapacitate the patient (does not include back pain). |
| **Interpretation** | Y. Admission is considered to be appropriate when any of the 18 criteria of the appropriateness of hospital admission is fulfilled. When none of the criteria are fulfilled, hospital admission is considered inappropriate. |
| **Reliability** | |
| **Inter-rater** | Independent application by 2 physicians on random sample of 85 registers of hospital discharges from which corresponding medical records were obtained. Overall agreement rate, the specific degree of agreement and the kappa index were calculated.  Overall agreement rate: 89%. Specific concordance: 40%. Kappa index: 0.5 |
| **Intra-rater** | NI |
| **Validity** | |
| **Convergent** | NI |
| **Costs** | |
| **Time to completion** | NI |
| **Specific input data required** | Y. For example, data on electrolyte (Na, K) or blood gas (CO2, arterial pH), electrocardiographic data. |
| **Specific training required** | NI |
| **Who completed the tool?** | |
| Physicians | |
| **When/where/how tool was/can be completed? (for example: at discharge/at admission, in hospital/in nursing home, retrospective/prospective)** | |
| In the study authors did a retrospective application using patient´s medical records. However, application of AEP during patient´s hospitalization could also be possible, but study authors note it would be more appropriate for retrospective use. | |
| **Language** | **Tool can be seen/accessed in:** |
| English | Annexe 1: ([22](#_ENREF_22)) |

**Table 10. AEPf (French version)**

| **Tool name (reported in (**[**23-26**](#_ENREF_23)**))** | |
| --- | --- |
| **AEPf (French version)** | |
| **Concept/components covered** | |
| 16 criteria in total. 10 criteria on clinical severity, 6 criteria on delivery of care. ([24](#_ENREF_24)) | |
| **What the tool measures/does** | |
| Appropriateness of hospital admissions. ([24](#_ENREF_24)) | |
| **Objectivity** | |
| **Process** | Y. Self-explaining. ([24](#_ENREF_24)) |
| **Evaluation** | Y. Has some parts, which may be subjective. For example, sudden impairment of essential functions (moving, eating, breathing, urinating, etc.) except for a chronic manifestation with no new facts. ([24](#_ENREF_24)) |
| **Interpretation** | Y. If one of 16 criteria is present, the admission is considered appropriate. ([23](#_ENREF_23)) |
| **Reliability** | |
| **Inter-rater** | The degree of agreement between two observers were assessed by the concordance and the Kappa coefficient. The reproducibility of the instrument was high (Kappa: 0.81). ([24](#_ENREF_24)) |
| **Intra-rater** | NI |
| **Validity** | |
| **Convergent** | NI |
| **Costs** | |
| **Time to completion** | Data were gathered from the patients themselves most of the time. It was easy to get the information from the record when necessary, most of the time in less than 5 min.([24](#_ENREF_24)) |
| **Specific input data required** | Y. For example, data on electrolyte (Na, K), ECG. ([24](#_ENREF_24)) |
| **Specific training required** | NI |
| **Who completed the tool?** | |
| Can be used by non-experts. AEPf was used by a physician and nurse ([24](#_ENREF_24)). Also was used by gerontologists and epidemiologists ([26](#_ENREF_26)). | |
| **When/where/how tool was/can be completed? (for example: at discharge/at admission, in hospital/in nursing home, retrospective/prospective)** | |
| In ED, prospective use. ([24](#_ENREF_24)) Retrospective use. ([23](#_ENREF_23)) | |
| **Language** | **Tool can be seen/accessed in:** |
| French ([24](#_ENREF_24)) | Annexe: ([24](#_ENREF_24)) |

**Table 11. AEPg (geriatric adaptation of AEP)**

| **Tool name (reported in (**[**23**](#_ENREF_23)**))** | |
| --- | --- |
| **AEPg (geriatric adaptation of AEP)** | |
| **Concept/components covered** | |
| 17 criteria in total. 11 criteria on clinical severity, 6 criteria on delivery of care. | |
| **What the tool measures/does** | |
| Appropriateness of hospital admissions. | |
| **Objectivity** | |
| **Process** | Y. Self- explaining. |
| **Evaluation** | Y. Some parts may be subjective. For example, sudden impairment of essential functions (moving, eating, breathing, urinating, etc.) except for a chronic manifestation with no new facts. |
| **Interpretation** | Y. If one criteria is present, admission is considered appropriate. |
| **Reliability** | |
| **Inter-rater** | NI |
| **Intra-rater** | NI |
| **Validity** | |
| **Convergent** | NI |
| **Costs** | |
| **Time to completion** | NI |
| **Specific input data required** | Y. For example, data on electrolyte (Na, K), ECG |
| **Specific training required** | NI |
| **Who completed the tool?** | |
| Geriatricians and a geriatric psychiatrist | |
| **When/where/how tool was/can be completed? (for example: at discharge/at admission, in hospital/in nursing home, retrospective/prospective)** | |
| In NH. Likely retrospective use. | |
| **Language** | **Tool can be seen/accessed in:** |
| French | Table 2: ([23](#_ENREF_23)) |

**Table 12. Adapted AEP**

| **Tool name (reported in (**[**27**](#_ENREF_27)**))** | |
| --- | --- |
| **Adapted AEP** | |
| **Concept/components covered** | |
| NI | |
| **What the tool measures/does** | |
| Appropriateness of hospital admissions. | |
| **Objectivity** | |
| **Process** | NI |
| **Evaluation** | NI |
| **Interpretation** | NI |
| **Reliability** | |
| **Inter-rater** | NI |
| **Intra-rater** | NI |
| **Validity** | |
| **Convergent** | NI |
| **Costs** | |
| **Time to completion** | NI |
| **Specific input data required** | NI |
| **Specific training required** | Y. Reported that 2 reviewers were previously trained to use the protocol (i.e. adapted AEP). |
| **Who completed the tool?** | |
| NI  Reported just on reviewers. | |
| **When/where/how tool was/can be completed? (for example: at discharge/at admission, in hospital/in nursing home, retrospective/prospective)** | |
| Hospital. Likely retrospective use. | |
| **Language** | **Tool can be seen/accessed in:** |
| NI. Likely to be in Portuguese | Tool not found. |

**Table 13. AEP Italian version**

| **Tool name (reported in (**[**28**](#_ENREF_28)**))** | |
| --- | --- |
| **AEP Italian version** | |
| **Concept/components covered** | |
| NI | |
| **What the tool measures/does** | |
| Appropriateness of hospital admissions. | |
| **Objectivity** | |
| **Process** | NI |
| **Evaluation** | NI |
| **Interpretation** | NI |
| **Reliability** | |
| **Inter-rater** | NI |
| **Intra-rater** | NI |
| **Validity** | |
| **Convergent** | NI |
| **Costs** | |
| **Time to completion** | NI |
| **Specific input data required** | NI |
| **Specific training required** | Y. Reviewers were given thorough preparation for the study using the literature on the subject and doing practice runs involving vast sample of medical records. |
| **Who completed the tool?** | |
| Reviewers: Qualified doctors. | |
| **When/where/how tool was/can be completed? (for example: at discharge/at admission, in hospital/in nursing home, retrospective/prospective)** | |
|  | |
| Hospital, concurrent method was used as opposed to longitudinal retrospective. | |
| **Language** | **Tool can be seen/accessed in:** |
| Likely Italian | Tool not found. |

**Table 14. AEP Spanish version**

| **Tool name (reported in (**[**29**](#_ENREF_29)**,** [**30**](#_ENREF_30)**))** | |
| --- | --- |
| **AEP Spanish version** | |
| **Concept/components covered** | |
| NI | |
| **What the tool measures/does** | |
| Appropriateness of hospital admissions. ([29](#_ENREF_29), [30](#_ENREF_30)) | |
| **Objectivity** | |
| **Process** | NI |
| **Evaluation** | NI |
| **Interpretation** | Y. Inappropriate admission is considered when on the day of admission the patient does not meet any criteria for hospitalization according to the AEP.([29](#_ENREF_29)) |
| **Reliability** | |
| **Inter-rater** | The degree of concordance by the three assessors following a short training period is kappa value of 0.31 for appropriate admissions.([29](#_ENREF_29))  The reviewers, four physicians participating in the study, reached an inter-observer agreement of more than 85% with a reviewer expert in the use of the AEP. ([30](#_ENREF_30)) |
| **Intra-rater** | NI |
| **Validity** | |
| **Convergent** | NI |
| **Costs** | |
| **Time to completion** | NI |
| **Specific input data required** | NI |
| **Specific training required** | Y. By way of training the assessors were given a theoretical and practical course on the use of the AEP.([29](#_ENREF_29)) |
| **Who completed the tool?** | |
| Internal resident doctor and ward nurses ([29](#_ENREF_29)) and physicians ([30](#_ENREF_30)). | |
| **When/where/how tool was/can be completed? (for example: at discharge/at admission, in hospital/in nursing home, retrospective/prospective)** | |
| In hospital, retrospective use of AEP using medical records of patients admitted to the hospital. ([29](#_ENREF_29)) | |
| **Language** | **Tool can be seen/accessed in:** |
| Likely to be in Spanish ([29](#_ENREF_29)) | Tool not found. |

**Table 15. Modified Italian AEP**

| **Tool name (reported in (**[**31**](#_ENREF_31)**))** | |
| --- | --- |
| **Modified Italian AEP** | |
| **Concept/components covered** | |
| Criteria of appropriateness of hospital admission divided in two subsets pertaining to severity of illness and patient conditions (n=11) and health care requirements or clinical services (n=7). ([32](#_ENREF_32)) | |
| **What the tool measures/does** | |
| Appropriateness of hospital admissions. ([31](#_ENREF_31)) | |
| **Objectivity** | |
| **Process** | NI |
| **Evaluation** | NI |
| **Interpretation** | Y. The admission to hospital is considered as appropriate when at least an established criterion is met. ([31](#_ENREF_31)) |
| **Reliability** | |
| **Inter-rater** | Inter-rater agreement and the k statistic for the assessment of admission were always higher than 85% and 0.82. ([32](#_ENREF_32)) |
| **Intra-rater** | NI |
| **Validity** | |
| **Convergent** | NI |
| **Costs** | |
| **Time to completion** | NI |
| **Specific input data required** | NI |
| **Specific training required** | NI |
| **Who completed the tool?** | |
| Likely study authors ([31](#_ENREF_31)) | |
| **When/where/how tool was/can be completed? (for example: at discharge/at admission, in hospital/in nursing home, retrospective/prospective)** | |
| at admission / in hospital / retrospective ([31](#_ENREF_31)) | |
| **Language** | **Tool can be seen/accessed in:** |
| Italian ([32](#_ENREF_32)) | Tool not found. |

1. **CURB-65 score**

**Table 16. CURB-65 score**

| **Tool name (reported in (**[**33**](#_ENREF_33)**,** [**34**](#_ENREF_34)**))** | |
| --- | --- |
| **CURB-65 score (available for hospital setting and slight adaptation for community setting)** | |
| **Concept/components covered** | |
| 5 clinical and laboratory characteristics (confusion, blood urea nitrogen, respiratory rate, blood pressure, and age >= 65 years). For community setting, blood urea nitrogen is absent. ([33-35](#_ENREF_33)) | |
| **What the tool measures/does** | |
| Mortality risk, assessment of severity in community acquired pneumonia. ([33-35](#_ENREF_33)) | |
| **Objectivity** | |
| **Process** | Y. Self-explaining. ([35](#_ENREF_35)) |
| **Evaluation** | Y. One element may be subjective by nature. In particular, assessment of confusion (measured by a mental test, or new disorientation in person, place or time). ([35](#_ENREF_35)) |
| **Interpretation** | Y. Available for 2 settings (hospital and community).  For hospital setting has max. score of 5, for community setting max. score is 4. Score of 1 is given for every element present.  For hospital setting: scores 0-1, 2, 3-5 indicate low, intermediate and high risk for mortality respectively.  For community setting: scores 0, 1-2, 3-4 indicate low, intermediate and high risk for mortality respectively. ([35](#_ENREF_35)) |
| **Reliability** | |
| **Inter-rater** | NI |
| **Intra-rater** | NI |
| **Validity** | |
| **Convergent** | The positive predictive value of the CURB-65 as an indicator of inappropriate hospitalization was determined by calculating the proportion of patients with CURB-65 score 0 and 1 in whom we were unable to find any justification for hospitalization, out of the total number of inpatients with a score 0 and 1, that is, by dividing the number of outpatients or unjustified patients who had been admitted to the hospital by the total number of patients with score 0 and 1.  The calculated positive predictive value of the CURB-65 score as a sole indicator for inappropriate hospitalization was 52%. ([33](#_ENREF_33)).  The sensitivity and specificity of the CURB-65 score of 2 or more—in the derivation cohort was 92.8% and 49.2%, respectively (with PPV and NPV in %: 16.2 and 98.5). Corresponding values in the validation cohort were 100% and 46.4% (with PPV and NPV in %: 16.1 and 100). ([35](#_ENREF_35)) |
| **Costs** | |
| **Time to completion** | NI |
| **Specific input data required** | Y. For hospital setting, blood urea testing is needed. ([35](#_ENREF_35)) |
| **Specific training required** | NI However, easy to use. “The CURB-65 score has been recommended by the British Thoracic Society, and because of its ease of use” ([34](#_ENREF_34)) |
| **Who completed the tool?** | |
| One infectious diseases, one internist and three pulmonary specialists ([33](#_ENREF_33)) | |
| **When/where/how tool was/can be completed? (for example: at discharge/at admission, in hospital/in nursing home, retrospective/prospective)** | |
| Can be used in hospital or community setting. Likely prospective use. ([35](#_ENREF_35)) Emergency room, retrospective by using patient´s records. ([33](#_ENREF_33)) Retrospective use, using clinical records of patients admitted to ED. ([34](#_ENREF_34)) | |
| **Language** | **Tool can be seen/accessed in:** |
| English. ([35](#_ENREF_35)) | Figures 2 & 3: ([35](#_ENREF_35)) |

1. **Risk Nomogram**

**Table 17. Risk Nomogram**

| **Tool name (reported in (**[**36**](#_ENREF_36)**))** | |
| --- | --- |
| **Risk Nomogram** | |
| **Concept/components covered** | |
| Prior number attendances, age, gender, polypharmacy, SIS cognition score, malignancy, CCT intervention, depression Hx. | |
| **What the tool measures/does** | |
| Probability of having no unplanned revisit during the 28 days after discharge. | |
| **Objectivity** | |
| **Process** | Y. Self-explaining. |
| **Evaluation** | Y. For example, component on depression (includes patient self-reporting of significant depressive syndromes). |
| **Interpretation** | Y. The final score is used to calculate the probability of having no unplanned revisit during the 28 days after discharge (where 1 is the probability of no attendance). |
| **Reliability** | |
| **Inter-rater** | NI |
| **Intra-rater** | NI |
| **Validity** | |
| **Convergent** | The ROC for the nomogram is shown in Fig.3, with an area under the curve of 0.65. |
| **Costs** | |
| **Time to completion** | NI |
| **Specific input data required** | N. Mostly data from medical record verified with patient interview. |
| **Specific training required** | Y. Research nurses trained for the study recruited patients and used patient interview in conjunction with the medical record and hospital electronic patient tracking systems to determine whether each of these factors were present. |
| **Who completed the tool?** | |
| Research nurses. | |
| **When/where/how tool was/can be completed? (for example: at discharge/at admission, in hospital/in nursing home, retrospective/prospective)** | |
| Prospective use, patients assessed in the ED and designated for discharge back to the community. | |
| **Language** | **Tool can be seen/accessed in:** |
| English | Figure 1 and table 1: ([36](#_ENREF_36)) |

1. **HOSPITAL score and its sub-type**

**Table 18. HOSPITAL score**

| **Tool name (reported in (**[**37-44**](#_ENREF_37)**))** | |
| --- | --- |
| **HOSPITAL score** | |
| **Concept/components covered** | |
| The HOSPITAL score is a predictor model using seven clinical variables at discharge. Haemoglobin level at discharge, Discharge from an Oncology unit, Sodium level at discharge, Procedures during hospital stay, Index admission type, Number of hospital admissions during the previous year, Length of hospitalisation. ([41](#_ENREF_41)) | |
| **What the tool measures/does** | |
| Risk of 30-day potentially avoidable hospital readmissions. ([41](#_ENREF_41)) | |
| **Objectivity** | |
| **Process** | Y. Self-explaining. ([41](#_ENREF_41)) |
| **Evaluation** | N. Does not include subjective elements. Some elements relate to laboratory testing data, like haemoglobin, sodium level. Other relate to ICD-9 coded procedure, admission type, number of admissions, LOS, discharge from an oncology service. ([41](#_ENREF_41)) |
| **Interpretation** | Y. The scoring system ranges from 0 to 13 points with  higher scores connoting higher risk of readmission. These risks were further categorized into 3 groups: low risk (up to 4  points); intermediate risk (5–6 points); and high risk (7 or more points), roughly corresponding to 5%, 10%, and 20% risk of potentially preventable 30-day readmissions, respectively. ([40](#_ENREF_40)) |
| **Reliability** | |
| **Inter-rater** | NI |
| **Intra-rater** | NI |
| **Validity** | |
| **Convergent** | The cross-validated C statistic was 0.69 in the derivation set and 0.71 in the validation set. When the HOSPITAL score was applied to the complete cohort before exclusion of unavoidable readmissions (n = 10 731), the cross-validated C statistic was 0.67. ([41](#_ENREF_41))  Sensitivity: 21%  Specificity: 80%  ([37](#_ENREF_37))  Across all 4 diagnoses, the HOSPITAL score had very good accuracy (Brier score = 0.11) good discrimination [c-statistic = 0.68 (95%CI, 0.66–0.70)], and very good calibration (Hosmer-Lemeshow goodness-of-fit P= 0.77). The expected and observed readmission rates were very similar within each risk subgroup: low risk (9.1% expected, 9.6% observed), moderate risk (11.3% expected, 11.0% observed), and high risk (18.0% expected, 18.1% observed). Within diagnoses, accuracy and discrimination were similar (Brier score, 0.10–0.12; c-statistic, 0.67–0.71), although calibration was better for pneumonia and COPD (P= 0.76 and 0.81, respectively) than for acute MI or HF (P= 0.16 and 0.17, respectively—Table 3).  ([40](#_ENREF_40))  The HOSPITAL score had a C statistic of 0.72 (95%CI, 0.72-0.72). The Brier score was 0.08. In US hospitals, the C statistic was 0.72 (95% CI, 0.71-0.72); Canada, 0.78 (95% CI, 0.76-0.80); Israel, 0.68 (95% CI, 0.67-0.69); and Switzerland, 0.68 (95% CI, 0.66-0.71).  In terms of calibration, the estimated risk of potentially avoidable readmission calculated with the HOSPITAL score matched the observed proportion of potentially avoidable readmissions in each risk group: 5.8% for the low risk group; 11.9%,intermediate; and 22.8%, high risk (Table 3). This is also reflected by an excellent Pearson χ2 test with a P value of 0.89. When calibration is analysed for each individual point score, calibration remains excellent except at the extreme ends of the range (low [0 points] and high [≥11 points]).  ([42](#_ENREF_42)) |
| **Costs** | |
| **Time to completion** | NI |
| **Specific input data required** | Y. Laboratory testing (haemoglobin, sodium level) ([41](#_ENREF_41)) |
| **Specific training required** | NI However reported to be easy to use ([42](#_ENREF_42)) |
| **Who completed the tool?** | |
| Physicians. ([41](#_ENREF_41)) | |
| **When/where/how tool was/can be completed? (for example: at discharge/at admission, in hospital/in nursing home, retrospective/prospective)** | |
| Can be used in hospital, prospective use, before discharge. ([41](#_ENREF_41)) Retrospective using electronic health records. ([40](#_ENREF_40)) | |
| **Language** | **Tool can be seen/accessed in:** |
| English | Table 3: ([41](#_ENREF_41)) |

**Table 19. Simplified HOSPITAL score**

| **Tool name (reported in (**[**39**](#_ENREF_39)**))** | |
| --- | --- |
| **Simplified HOSPITAL score** | |
| **Concept/components covered** | |
| The HOSPITAL score is a predictor model using six clinical variables at discharge. Haemoglobin level at discharge, Cancer diagnosis or discharge from an Oncology unit, Sodium level at discharge, Index admission type, Number of hospital admissions during the previous year, Length of hospitalisation. | |
| **What the tool measures/does** | |
| Risk of 30-day potentially avoidable hospital readmissions. | |
| **Objectivity** | |
| **Process** | Y. Self-explaining. |
| **Evaluation** | N. Does not include subjective elements. |
| **Interpretation** | Y. Scoring system, max. score is 12.  Unlikely to be readmitted if 0-4 point(s), and likely to be readmitted if 5 points or more.  These categories were created for ease of interpretation, roughly corresponding to a risk of potentially avoidable readmission of more than 15% in the “likely” category. |
| **Reliability** | |
| **Inter-rater** | NI |
| **Intra-rater** | NI |
| **Validity** | |
| **Convergent** | 1. Brier score of 0.08.  2. C-statistic of 0.69 (95%CI 0.68-0.69). The negative predictive value of the simplified HOSPITAL score was 94%, and its specificity 73%.  3. The calibration was excellent with predicted rates matching exactly the observed rates, as shown in Table 3a. |
| **Costs** | |
| **Time to completion** | NI |
| **Specific input data required** | Y. Laboratory testing (haemoglobin, sodium level) |
| **Specific training required** | NI. Reported to be easier to calculate as opposed to original HOSPITAL score. |
| **Who completed the tool?** | |
| Study authors. | |
| **When/where/how tool was/can be completed? (for example: at discharge/at admission, in hospital/in nursing home, retrospective/prospective)** | |
| In hospital, retrospective use. Can be used before discharge. | |
| **Language** | **Tool can be seen/accessed in:** |
| English | Table 1: ([39](#_ENREF_39)) |

1. **LACE index and its sub-type**

**Table 20. LACE index**

| **Tool name (reported in (**[**37**](#_ENREF_37)**,** [**44**](#_ENREF_44)**,** [**45**](#_ENREF_45)**))** | |
| --- | --- |
| **LACE index** | |
| **Concept/components covered** | |
| Length of hospitalisation, Acuteness of the admission, Comorbidities of patients, AED admissions. ([37](#_ENREF_37), [45](#_ENREF_45), [46](#_ENREF_46)) | |
| **What the tool measures/does** | |
| Expected probability of death or hospital readmission within 30 days of discharge. ([37](#_ENREF_37), [45](#_ENREF_45), [46](#_ENREF_46)) | |
| **Objectivity** | |
| **Process** | Y. Self-explaining. ([46](#_ENREF_46)) |
| **Evaluation** | Y. Component “acuity of admission” may involve some subjective judgement. ([46](#_ENREF_46)) |
| **Interpretation** | Y. Scoring system, max. points is 19. Table 4 in the original study (([46](#_ENREF_46))) provides the expected probability of death or readmission within 30 days of discharge. Score of 0 and 19 correspond to 2% and 43.7% expected probability of death or readmission respectively. ([46](#_ENREF_46))  A patient with a score greater than 10 is considered at high risk for unplanned hospital readmission.([37](#_ENREF_37)) |
| **Reliability** | |
| **Inter-rater** | NI |
| **Intra-rater** | NI |
| **Validity** | |
| **Convergent** | The LACE index had moderate discrimination for early death or readmission. The C statistic (95% CI) in the derivation was 0.7114 (0.6736–0.7491). In the validation, it was 0.6935 (0.6548–07321), and in the entire cohort, it was 0.7025 (0.6755–0.7295). ([46](#_ENREF_46))  Sensitivity (cut-off > 10): 0.61  Specificity (cut-off > 10): 0.44  PPV (cut-off > 10): 0.52  NPV (cut-off > 10): 0.54  Discrimination: AUC (i.e. c-statistic) = 0.534  Calibration (Hosmer-Lemeshow χ2 test): χ2 = 23.58, degrees of freedom = 23, P-value = 0.43  ([37](#_ENREF_37))    The optimal cut-off for the LACE index is a score of 7 or more with sensitivity of 0.752 and specificity of 0.564. AUC (c-statistic) is 0.658.  ([45](#_ENREF_45)) |
| **Costs** | |
| **Time to completion** | NI |
| **Specific input data required** | Y. Calculation of another index to measure comorbidity (using Charlson comorbidity index).([46](#_ENREF_46)) |
| **Specific training required** | NI |
| **Who completed the tool?** | |
| Study authors ([37](#_ENREF_37)) | |
| **When/where/how tool was/can be completed? (for example: at discharge/at admission, in hospital/in nursing home, retrospective/prospective)** | |
| In hospital, retrospective using medical records. ([37](#_ENREF_37)) | |
| **Language** | **Tool can be seen/accessed in:** |
| English([46](#_ENREF_46)) | Tool in Table 3, conversation of a score to % expected probability of death or readmission is in Table 4: ([46](#_ENREF_46)) |

**Table 21. Revised LACE index**

| **Tool name (reported in (**[**47**](#_ENREF_47)**))** | |
| --- | --- |
| **Revised LACE index** (LOS component was omitted as compared with original LACE) | |
| **Concept/components covered** | |
| Acuteness of the admission, Comorbidities of patients, AED admissions. | |
| **What the tool measures/does** | |
| Risk of hospital readmission within 30 days of discharge. | |
| **Objectivity** | |
| **Process** | NI  However likely to be same as in original LACE. |
| **Evaluation** | NI  However likely to be same as in original LACE. |
| **Interpretation** | NI  Max. possible score not reported. However, revised LACE score 8 or above is considered as high risk of early readmission. |
| **Reliability** | |
| **Inter-rater** | NI |
| **Intra-rater** | NI |
| **Validity** | |
| **Convergent** | Sensitivity and specificity for the revised LACE index were 0.49 (95% CI 0.39–0.59) and 0.60 (95% CI 0.57–0.64), respectively, with NPV 0.90 (95% CI 0.87–0.92) and PPV 0.15 (95% CI 0.11–0.19). |
| **Costs** | |
| **Time to completion** | Y. Under 10 min. |
| **Specific input data required** | NI  However likely to be same as in original LACE. |
| **Specific training required** | Y. Only mentioned that researchers were trained in conducting assessment. |
| **Who completed the tool?** | |
| Researchers. | |
| **When/where/how tool was/can be completed? (for example: at discharge/at admission, in hospital/in nursing home, retrospective/prospective)** | |
| In hospital, prospective use, used at admission of a patient to hospital (as opposed to before discharge in the original LACE). | |
| **Language** | **Tool can be seen/accessed in:** |
| English | Tool not found. |

1. **New Zealand version of Patients At Risk of Hospital Readmission (PARR) predictive risk tool**

**Table 22. New Zealand version of Patients At Risk of Hospital Readmission (PARR) predictive risk tool**

| **Tool name (reported in (**[**45**](#_ENREF_45)**))** | |
| --- | --- |
| **New Zealand version of Patients At Risk of Hospital Readmission (PARR) predictive risk tool** | |
| **Concept/components covered** | |
| Use of admissions data from the New Zealand hospitals.  (gender, age, race (Maori, Pacific, Asian, others), cost weight of last admission, code for last submission, diagnoses for last admission and number of acute admissions in the previous 90 days, 180 days and 2 years). | |
| **What the tool measures/does** | |
| Prediction of 30-day hospital readmissions. | |
| **Objectivity** | |
| **Process** | NI |
| **Evaluation** | N. From description in the study, does not seem to include subjective judgement. |
| **Interpretation** | NI |
| **Reliability** | |
| **Inter-rater** | NI |
| **Intra-rater** | NI |
| **Validity** | |
| **Convergent** | The optimal cut-off for PARR index is a score of 0.34 or more with sensitivity of 0.542 and specificity of 0.714. AUC (i.e. c-statistic) = 0.628. |
| **Costs** | |
| **Time to completion** | NI |
| **Specific input data required** | N. From description in the study, does not seem to require specific data (such as laboratory results). |
| **Specific training required** | NI |
| **Who completed the tool?** | |
| NI | |
| **When/where/how tool was/can be completed? (for example: at discharge/at admission, in hospital/in nursing home, retrospective/prospective)** | |
| Retrospective using admissions data from hospitals. | |
| **Language** | **Tool can be seen/accessed in:** |
| Likely to be in English | Tool not found. |

1. **PAR-Risk Score**

**Table 23. PAR-Risk Score**

| **Tool name (reported in (**[**48**](#_ENREF_48)**))** | |
| --- | --- |
| **PAR-Risk Score** | |
| **Concept/components covered** | |
| The PAR Risk Score assigns points to the following 12 predictors: length of stay longer than four days, admission in previous six months, anaemia, hypertension, hyperkalaemia, opioid prescription during hospital stay, comorbidities such as heart failure, acute myocardial infarction, chronic ischemic heart disease, diabetes with organ damage, cancer, and metastatic carcinoma. | |
| **What the tool measures/does** | |
| Risk of 30-day potentially avoidable readmissions. | |
| **Objectivity** | |
| **Process** | Y. Self-explaining. |
| **Evaluation** | N. Does not include subjective judgement. Requires data on administrative characteristics, comorbidities, medications and lab results. |
| **Interpretation** | Y. Low, medium, and high risk based on the raw PAR-Risk Score values using the original threshold levels of <3, 3–10, and>10, respectively.  Adapted threshold levels for the three risk categories low, medium, and high risk were PAR-Risk Score values of <12, 12 to 25, and >25, respectively. |
| **Reliability** | |
| **Inter-rater** | NI |
| **Intra-rater** | NI |
| **Validity** | |
| **Convergent** | The overall PAR-Risk Scores showed C statistic of 0.605, 95% -CI 0.575–0.635.  The Brier score was 0.053.  The calibration plot indicated a lack of fit (Fig3), which was also supported by the goodness-of-fit test with a p-value of <0.01. A summary of the goodness-of-fit test statistics provided in the supplement (S3Table).  **Original threshold:**  Positive Predictive Value (%): low vs. medium 4.3; low vs. high 7.5  Negative Predictive Value (%): low vs. medium 97.0 ; low vs. high 97.0.  Sensitivity (%): low vs. medium 93.4; low vs. high 95.8  Specificity (%): low vs. medium 9.4; low vs. high 10.4  **Adapted threshold:**  Positive Predictive Value (%): low vs. medium 6.0 ; low vs. high 7.9  Negative Predictive Value (%): low vs. medium 96.6 ; low vs. high 96.6  Sensitivity (%): low vs. medium 61.7; low vs. high 67.9  Specificity (%): low vs. medium 52.9; low vs. high 53.4 |
| **Costs** | |
| **Time to completion** | NI |
| **Specific input data required** | Y. For example, lab results on hyperkalaemia. |
| **Specific training required** | NI |
| **Who completed the tool?** | |
| Study authors. | |
| **When/where/how tool was/can be completed? (for example: at discharge/at admission, in hospital/in nursing home, retrospective/prospective)** | |
| Retrospective using data from hospitalisations. | |
| **Language** | **Tool can be seen/accessed in:** |
| English | S1 table: ([48](#_ENREF_48)) |

1. **EOL care pathway**

**Table 24. EOL care pathway**

| **Tool name (reported in (**[**49**](#_ENREF_49)**))** | |
| --- | --- |
| **EOL care pathway** | |
| **Concept/components covered** | |
| The EOL care pathway used in the Good Death project had five main sections: (i) commencing a pathway; (ii) medical interventions and advance care planning; (iii) care staff interventions, including care management, daily comfort care chart and further care action sheet; (iv) multidisciplinary communication sheet; and (v) after-death care. ([49](#_ENREF_49), [50](#_ENREF_50)) | |
| **What the tool measures/does** | |
| Provides guidance on different aspects of terminal care. ([49](#_ENREF_49), [50](#_ENREF_50)) | |
| **Objectivity** | |
| **Process** | Y. Self-explaining. ([50](#_ENREF_50)) |
| **Evaluation** | Y. Some components may be subjective. For example, profound weakness, becoming semi-conscious with lapses into unconsciousness, changes in breathing patterns. ([50](#_ENREF_50)) |
| **Interpretation** | N. Does not seem to provide a certain final judgement. This is a guide to providing care for residents in RACFs during last days of their lives. The entire document (pathway) forms part of the resident´s medical record. ([50](#_ENREF_50)) |
| **Reliability** | |
| **Inter-rater** | NI |
| **Intra-rater** | NI |
| **Validity** | |
| **Convergent** | NI |
| **Costs** | |
| **Time to completion** | NI However, the pathway is a 12 page document, which may take some time to complete it. ([50](#_ENREF_50)) |
| **Specific input data required** | Y. Some data may be seen as specific, for example Advance care planning (if not yet completed). Also, further data may be needed via discussion with the resident or his/her representative. However, some data may already be documented in the resident´s chart and taken from there. ([50](#_ENREF_50)) |
| **Specific training required** | NI |
| **Who completed the tool?** | |
| GPs, nurses. ([50](#_ENREF_50)) | |
| **When/where/how tool was/can be completed? (for example: at discharge/at admission, in hospital/in nursing home, retrospective/prospective)** | |
| In RACF, prospective use. ([50](#_ENREF_50)) | |
| **Language** | **Tool can be seen/accessed in:** |
| English ([50](#_ENREF_50)) | Pathway can be found on this page: ([50](#_ENREF_50)).  Direct link to the PDF file: <https://metrosouth.health.qld.gov.au/sites/default/files/content/raceolcp_watermark.pdf> |

1. **The Identification of Seniors at Risk (ISAR) scale**

**Table 25. The Identification of Seniors at Risk (ISAR) scale**

| **Tool name (reported in (**[**51**](#_ENREF_51)**))** | |
| --- | --- |
| **The Identification of Seniors at Risk (ISAR) scale** | |
| **Concept/components covered** | |
| Takes into account function (premorbid and post-acute change), polypharmacy, cognitive and visual impairment, and recent hospitalizations. ([51](#_ENREF_51), [52](#_ENREF_52)) | |
| **What the tool measures/does** | |
| Predicts high acute care hospital utilization and adverse health outcomes during the 6 months after the ED visit/after home discharge. ([51](#_ENREF_51), [52](#_ENREF_52)) | |
| **Objectivity** | |
| **Process** | Y. Self-explaining. ([52](#_ENREF_52)) |
| **Evaluation** | Y. First, the tool can be filled by a patient too, which may already be subjective. Second, it includes questions which may also include subjective judgement, like “in general, do you see well?”, or “in general, do you have serious problems with your memory?”. ([52](#_ENREF_52)) |
| **Interpretation** | Y. 6 question tool, max. 6 points. ISAR score of 2 or higher, indicating an increased risk of adverse health outcomes. ([52](#_ENREF_52)) |
| **Reliability** | |
| **Inter-rater** | NI |
| **Intra-rater** | NI |
| **Validity** | |
| **Convergent** | Y. The AUC for the ISAR scale was 0.68 overall. ([52](#_ENREF_52)).  Using ROC (combined) curves, ISAR showed the best prediction among other variables, although predictive value was poor (AUC=0.62 (0.53-0.71) for ISAR>3 and AUC=0.65 (0.57-0.74) for continuous ISAR). ([51](#_ENREF_51)). |
| **Costs** | |
| **Time to completion** | Y. Can be quickly and easily administered. ([52](#_ENREF_52)) |
| **Specific input data required** | N. Questions can also be answered by a patient. ([52](#_ENREF_52)) |
| **Specific training required** | NI However, reported to be easy to use, can be used by patients. ([52](#_ENREF_52)) |
| **Who completed the tool?** | |
| Can be self-completed by many patients or informants. ([52](#_ENREF_52)) | |
| **When/where/how tool was/can be completed? (for example: at discharge/at admission, in hospital/in nursing home, retrospective/prospective)** | |
| In ED setting. Can be used at admission or discharge from the ED. Prospective use. ([52](#_ENREF_52)) | |
| **Language** | **Tool can be seen/accessed in:** |
| English ([52](#_ENREF_52)) | Figure 1: ([52](#_ENREF_52)) |

1. **The Silver Code**

**Table 26. The Silver Code**

| **Tool name (reported in (**[**51**](#_ENREF_51)**))** | |
| --- | --- |
| **The Silver Code** | |
| **Concept/components covered** | |
| Combines demographics, polypharmacy, comorbidities, and previous hospitalizations. ([51](#_ENREF_51), [53](#_ENREF_53)) | |
| **What the tool measures/does** | |
| Predicts 1-year mortality, hospital admission, ED readmissions. ([51](#_ENREF_51), [53](#_ENREF_53)) | |
| **Objectivity** | |
| **Process** | Y. Self-explaining. ([53](#_ENREF_53)) |
| **Evaluation** | N. With the SC, a score is assigned to age, sex, marital status, admission to a day hospital, admission to regular ward with corresponding discharge diagnosis, and polypharmacy, 3–6 months prior to the index ED visit. ([53](#_ENREF_53)) |
| **Interpretation** | Y. Max. 30 points. 4 classes of increasing risk score (0–3, 4–6, 7–10, and 11+). ([53](#_ENREF_53))  Score 0 - 30, best - worst ([51](#_ENREF_51)) |
| **Reliability** | |
| **Inter-rater** | NI |
| **Intra-rater** | NI |
| **Validity** | |
| **Convergent** | Y. Area under the receiver-operating characteristic curve in predicting hospital admission (SC: 0.63) and mortality (SC: 0.70). ([53](#_ENREF_53))  AUC (combined) for Silver Code: 0.56 (95% CI, 0.46-0.65)  AUC (combined) for Silver Code > 11: 0.53 (95% CI, 0.44-0.62)  ([51](#_ENREF_51)) |
| **Costs** | |
| **Time to completion** | NI |
| **Specific input data required** | N. It is based on administrative data ([53](#_ENREF_53)) |
| **Specific training required** | NI |
| **Who completed the tool?** | |
| Expert physicians or nurses ([51](#_ENREF_51)) | |
| **When/where/how tool was/can be completed? (for example: at discharge/at admission, in hospital/in nursing home, retrospective/prospective)** | |
| Used retrospectively using administrative data from the ED. However, can also be used prospectively if data is available. It is based on administrative data, which would be virtually available even before patients access the ED. ([53](#_ENREF_53)) | |
| **Language** | **Tool can be seen/accessed in:** |
| English ([53](#_ENREF_53)) | Table 1: ([53](#_ENREF_53)) |

1. **The Walter indicator**

**Table 27. The Walter indicator**

| **Tool name (reported in (**[**51**](#_ENREF_51)**))** | |
| --- | --- |
| **The Walter indicator** | |
| **Concept/components covered** | |
| Combines demographics, clinical aspects (heart failure, cancer with or without metastases), and laboratory testing (albumin, creatinine). ([51](#_ENREF_51), [54](#_ENREF_54)) | |
| **What the tool measures/does** | |
| Predicts 1-year mortality after hospital discharge. ([51](#_ENREF_51), [54](#_ENREF_54)) | |
| **Objectivity** | |
| **Process** | Y. Self-explaining. ([54](#_ENREF_54)) |
| **Evaluation** | N. Does not have elements requiring subjective judgement. Includes demographic characteristics, activities of daily living (ADL) dependency, comorbid conditions, length of hospital stay, and laboratory measurements. ([54](#_ENREF_54)) |
| **Interpretation** | Y. Scoring system. Max. score 20.  Lowest-risk group (0-1 point).  Group with 2-3 points  Group with 4-6 points  Highest risk group with more than 6 points.  In the validation cohort, 1-year mortality was 4% in the lowest-risk group, 19% in the group with 2 or 3 points, 34% in the group with 4 to 6 points, and 64% in the highest-risk group. ([54](#_ENREF_54)) |
| **Reliability** | |
| **Inter-rater** | NI |
| **Intra-rater** | NI |
| **Validity** | |
| **Convergent** | The area under the receiver operating characteristic curve for the point system was 0.75 in the derivation cohort and 0.79 in the validation cohort. ([54](#_ENREF_54))  AUC (combined) for Walter indicator: 0.64 (95% CI, 0.55-0.73)  AUC (combined) for Walter indicator > 6: 0.55 (95% CI, 0.45-0.65)  ([51](#_ENREF_51)) |
| **Costs** | |
| **Time to completion** | NI |
| **Specific input data required** | Y. Lab results (creatinine, albumin) ([54](#_ENREF_54)) |
| **Specific training required** | NI  However, the tool has a simple additive point system. ([54](#_ENREF_54)) |
| **Who completed the tool?** | |
| Study authors. Can also be used by clinicians. | |
| **When/where/how tool was/can be completed? (for example: at discharge/at admission, in hospital/in nursing home, retrospective/prospective)** | |
| In hospital, at discharge. Prospective use (validated prospectively). Tool uses data that should be available at discharge. ([54](#_ENREF_54)) | |
| **Language** | **Tool can be seen/accessed in:** |
| English ([54](#_ENREF_54)) | Table 3: ([54](#_ENREF_54)) |

1. **Preventability Assessment Tool (PAT)**

**Table 28. Preventability Assessment Tool (PAT)**

| **Tool name (reported in (**[**55**](#_ENREF_55)**))** | |
| --- | --- |
| **Preventability Assessment Tool (PAT)** | |
| **Concept/components covered** | |
| Includes several factors (with corresponding sub-sections) that may have been related to a patient admission.  Patient factors, self-care, primary care factors, coordination of care, access to care (clinical and non-clinical), hospital admission characteristics, other factors. | |
| **What the tool measures/does** | |
| Assessment of preventability of unplanned hospital admissions for chronic conditions. | |
| **Objectivity** | |
| **Process** | Y. Self-explaining. |
| **Evaluation** | Y. A section of the tool asks the extent to which some factors in the previous 3 months may have been related to a patient´s unplanned admission. Some factors may seem subjective when making judgement on them. For example, patient factors (includes, but not limited to: cognitive function, mental health problems). |
| **Interpretation** | N. The tool does not have a specific rule to judge how preventable an admission was. Instead, the tool asks how preventable an admission was, considering all that has happened to a patient in the last 3 months (within the context of the study definition of preventability; definition given in the tool). |
| **Reliability** | |
| **Inter-rater** | Agreement between the assessments of the hospital doctors and nurses regarding which admissions were deemed preventable were (Κ = 0.21; 95% CI = 0.09–0.34). The agreement between hospital nurses and hospital doctors for admissions being preventable was 18%, agreement for non-preventable admissions (including admissions assessed as not preventable and those unclassifiable) was higher at 46%. Overall disagreement between the hospital nurses and hospital doctors was 36%. |
| **Intra-rater** | NI |
| **Validity** | |
| **Convergent** | There was very low agreement between the Expert Panels and the hospital nurse regarding the assessment of the preventability of individual admissions (K = 0.17; 95% CI = 0.05–0.28) (see Table 3). Of the 119 admissions assessed as preventable by Expert Panel, only 53 (45%) were assessed as preventable by the hospital nurses. Similarly, there was very low agreement between the Expert Panel and the hospital doctor regarding the assessment of the preventability of individual admissions (K = 0.13; 95% CI = 0.01–0.25). Of the 119 admissions assessed as preventable by Expert Panel, only 51 (45%) were assessed as preventable by the hospital doctors. |
| **Costs** | |
| **Time to completion** | Y. About 5 min. to complete |
| **Specific input data required** | N. Does not require laboratory testing results. However, observer would require data on several components, like data on patient factors, self-care factors, primary care factors, etc. |
| **Specific training required** | Y. The research nurse provided brief explanations of how to use the PAT to assess admissions within the context of the study definition of preventability, and was available to answer questions. |
| **Who completed the tool?** | |
| Hospital clinicians (doctor or nurse caring for the patient). | |
| **When/where/how tool was/can be completed? (for example: at discharge/at admission, in hospital/in nursing home, retrospective/prospective)** | |
| In hospital, prospective use, during admission. | |
| **Language** | **Tool can be seen/accessed in:** |
| English | Supplement 1. ([55](#_ENREF_55))  Direct link to the Supplement 1: [Link](https://oup.silverchair-cdn.com/oup/backfile/Content_public/Journal/fampra/37/3/10.1093_fampra_cmz086/1/cmz086_suppl_supplementary_material_1.pdf?Expires=1691511899&Signature=C2yIKyq7j-hvKyDu5x~CZCS5YAnP~Hsr--LC-r0Uo91dBh6FMCvhXiGDV29Kisdcl49MetVRNP4UEUWgN5vGRSFv8ZVkIRyaFbgldaVcM8Sf-QU~YWTTxkZpOKgvjWdlAtxvB27UhBUkp~hP6sGuIIWSYm0ek5k6NYCJ80MYvnaPiM3Oh1KelJgOtqKcSsS6wS-1PGMq0VCIQfCIX5fESm5ZqHipmFGdvt6FQRUcH35U0LBQ~6zJa2K2Mb1jVhEePrtRRyw~lTj6Xq-anShzcu3BxdaW1QYvqu1u4Q4US8A3MitwFBsvqgCPc3NU2QpZzLAFk18wtCOMr6BeEJi2TA__&Key-Pair-Id=APKAIE5G5CRDK6RD3PGA) |

1. **Quality assessment instrument**

**Table 29. Quality assessment instrument**

| **Tool name (reported in (**[**56**](#_ENREF_56)**))** | |
| --- | --- |
| **Quality assessment instrument** | |
| **Concept/components covered** | |
| Patient, clinician, and system factors. | |
| **What the tool measures/does** | |
| Classifies the preventability of hospitalization in terms of patient, clinician, or system factors. | |
| **Objectivity** | |
| **Process** | Y. Self-explaining. |
| **Evaluation** | Y. Some elements may include subjective judgement. For example. Part 1 asks to select 1 primary reason out of 6 reasons for admission. Part 2 asks if an admission was preventable, and if yes, it asks to choose 1 of 13 reasons that could have prevented an admission. |
| **Interpretation** | N. Does not have a specific rule/guidance to judge if an admission was preventable. Tool provides a set of reasons and asks to choose one of them. In determining the reason for admission (one of six categories) and the reason for preventable admissions (one of 13 categories), a simple majority rule was applied. |
| **Reliability** | |
| **Inter-rater** | Agreement of preventability of readmissions was 74%, K= 0.43 (95% CI 0.36, 0.50). |
| **Intra-rater** | Agreement for the assessment of preventability of readmissions was 96%, K= 0.89 (95% CI 0.68, 1.0). |
| **Validity** | |
| **Convergent** | NI |
| **Costs** | |
| **Time to completion** | NI |
| **Specific input data required** | N. Does not require specific input data, such as laboratory testing results. However, assessor should have access to some data (can be available from medical records). For example, in the study authors retrieved data from medical records on: outpatient notes for the 1 month period before readmission, ED note or clinic note on the day of admission, all records of admission histories and physical examinations, admitting nursing evaluation, admitting orders, any laboratory or radiologic information that was available within 24 hours of admission. |
| **Specific training required** | Y. Panellists received 45 min. of instructions, during which the rating form (tool) was reviewed and examples of each type of preventable hospitalization were discussed. |
| **Who completed the tool?** | |
| A panel of 10 board certified internists, 8 had administrative responsibility for the ambulatory practice in hospitals, all practiced clinical medicine. | |
| **When/where/how tool was/can be completed? (for example: at discharge/at admission, in hospital/in nursing home, retrospective/prospective)** | |
| In hospital, retrospective using medical records. | |
| **Language** | **Tool can be seen/accessed in:** |
| English | Appendix: ([56](#_ENREF_56)) |

1. **SIR (structured implicit record review)**

**Table 30. SIR (structured implicit record review)**

| **Tool name (reported in (**[**5**](#_ENREF_5)**,** [**57**](#_ENREF_57)**))** | |
| --- | --- |
| **SIR (structured implicit record review)** | |
| **Concept/components covered** | |
| Series of items about the resident and circumstances surrounding the hospitalization. These relate to residents´ baseline health status, advance directives, potential benefits of acute transfer, and the care provided in the NH when the residents’ status changed.  In particular, the following factors were identified as affecting the transfer decision: (1) the resident’s baseline state, including demographic characteristics and care preferences; (2) characteristics of the acute illness, including the severity of the acute illness, the existence of known interventions for the illness, the urgency of the need for examination and the response to SNF based treatments; (3) clinical care resources necessary and typically available to manage the acute illness, including physician services, SNF based services and services typically available only outside of the SNF; and (4) the quality of acute care in the SNF. ([5](#_ENREF_5), [57](#_ENREF_57)) | |
| **What the tool measures/does** | |
| Assessment of inappropriateness of ED transfers and hospital admissions and factors related to inappropriateness. ([5](#_ENREF_5), [57](#_ENREF_57)) | |
| **Objectivity** | |
| **Process** | Y. Reviewers required to answer SIR questions, and after responding to SIR questions, the reviewer was asked: ‘‘Was this hospitalization avoidable?’’ Response categories included the following: definitely not avoidable, probably not avoidable, probably avoidable, and definitely avoidable. ([5](#_ENREF_5))  **However, complete tool not found.**  SIR has questions (addressed as SIR questions) which seem to guide reviewers in completing the SIR. A framework for SIR Form was shown in the study ([57](#_ENREF_57)), however the actual SIR questions could not be found. The framework for SIR Form shows items addressed in SIR Form grouped into overarching general topics. ([57](#_ENREF_57)). |
| **Evaluation** | Y. Some items addressed in SIR Form may be subjective. For example items on: pain, probability of death/pain. ([57](#_ENREF_57))  **However, complete tool not found.** |
| **Interpretation** | Y. Reviewers were instructed to rate a transfer or admission appropriate when no lower level of care would suffice to deliver safely the services the resident required. ([57](#_ENREF_57))  **However, complete tool not found.** |
| **Reliability** | |
| **Inter-rater** | Information about resident’s preferences or AD, if any, had little or no effect on agreement or interrater reliability. When rating the appropriateness of ED transfer, the two independent physician reviewers agreed with each other 84% of time (kappa .68) when excluding consideration of preferences, and 85% of the time (kappa .70) when considering preferences. Interrater reliability was even higher for hospital transfers. When rating the appropriateness of hospital admission, the reviewers agreed with each other 89% of the time (kappa .78) whether or not preferences were considered.  ([57](#_ENREF_57)) |
| **Intra-rater** | NI |
| **Validity** | |
| **Convergent** | NI |
| **Costs** | |
| **Time to completion** | NI |
| **Specific input data required** | Y. Data from SNF, ED and hospital records were used to complete SIR. For example, data from SNF record included information on nursing and physician notes, laboratory and radiology reports, and more. ([57](#_ENREF_57)) |
| **Specific training required** | Y. The reviewers received 2 days of training. Before the training, each reviewer received a 34-page instruction manual explaining the SIR questions. ([57](#_ENREF_57)) |
| **Who completed the tool?** | |
| Physicians. ([57](#_ENREF_57))  Experts in nursing home care and experienced practicing long-term care clinicians. ([5](#_ENREF_5)) | |
| **When/where/how tool was/can be completed? (for example: at discharge/at admission, in hospital/in nursing home, retrospective/prospective)** | |
| Physicians used SIR to review SNF and hospital records. Likely retrospective use. ([57](#_ENREF_57)). | |
| **Language** | **Tool can be seen/accessed in:** |
| English ([57](#_ENREF_57)) | See Table 1 for Framework for SIR Form: ([57](#_ENREF_57)) |

1. **Rectal bleeding admission guide and algorithm**

**Table 31. Rectal bleeding admission guide and algorithm**

| **Tool name (reported in (**[**58**](#_ENREF_58)**))** | |
| --- | --- |
| **Rectal bleeding admission guide and algorithm** | |
| **Concept/components covered** | |
| Haemoglobin (Hb) > 13 g/dl; Systolic blood pressure (SBP) > 115 mmHg; Patient not on anticoagulant/antiplatelet therapy. | |
| **What the tool measures/does** | |
| Identifies patients with acute LGIB (lower gastrointestinal bleeding) who can be safely managed in primary care. | |
| **Objectivity** | |
| **Process** | Y. Self-explaining. |
| **Evaluation** | N. Includes the following elements  1. Haemoglobin (Hb) > 13 g/dl;  2. Systolic blood pressure (SBP) > 115 mmHg;  3. Patient not on anticoagulant/antiplatelet therapy |
| **Interpretation** | Y. If ALL 3 criteria are true, patient will not usually require admission. |
| **Reliability** | |
| **Inter-rater** | NI |
| **Intra-rater** | NI |
| **Validity** | |
| **Convergent** | NI |
| **Costs** | |
| **Time to completion** | NI |
| **Specific input data required** | Y. Laboratory testing, haemoglobin. |
| **Specific training required** | Y. This new, simple risk assessment tool was adapted into a clinical algorithm and promoted with posters and education of staff in the surgical admissions ward and ED (Fig. 1).  This (i.e. criteria/instrument) was implemented with education of primary and secondary care staff, access to an emergency clinic and provision of patient information. |
| **Who completed the tool?** | |
| ED clinicians and general practitioners (GPs). | |
| **When/where/how tool was/can be completed? (for example: at discharge/at admission, in hospital/in nursing home, retrospective/prospective)** | |
| In surgical admissions ward and ED. On admission. Likely prospective use. Hospital setting, Acute setting. Useful in community referrals. | |
| **Language** | **Tool can be seen/accessed in:** |
| English | Figure 1. ([58](#_ENREF_58)) |

1. **Potentially Avoidable Readmission (PAR) algorithm**

**Table 32. Potentially Avoidable Readmission (PAR) algorithm**

| **Tool name (reported in (**[**59**](#_ENREF_59)**))** | |
| --- | --- |
| **Potentially Avoidable Readmission (PAR) algorithm** | |
| **Concept/components covered** | |
| Sort of mathematical methods. Authors proposed a new readmission metric to identify potentially avoidable readmissions, and a tree-based classification method to estimate the predicted probability of readmission that can directly incorporate patient’s history of readmission and risk factors changes over time. | |
| **What the tool measures/does** | |
| Identifies potentially avoidable readmissions, estimates the predicted probability of readmission. | |
| **Objectivity** | |
| **Process** | Sort of mathematical algorithm (phase-type survival forest = tree based method). |
| **Evaluation** | N.  Does not seem to involve subjective judgement. Method incorporates patient’s history of readmission and risk factors changes over time. |
| **Interpretation** | It seems that final judgement is produced by this method/algorithm in the form of predicted probability of readmission (i.e. potentially avoidable readmission). |
| **Reliability** | |
| **Inter-rater** | NI |
| **Intra-rater** | NI |
| **Validity** | |
| **Convergent** | In the **baseline model,** the c-statistics was 0.793.  In the **calibrated model**, the c-statistics was 0.836.  **Model validation**.  The calibrated model was used and its **internal validity** (also called reproducibility) was studied, based on the same population underlying the sample. The average c-statistics for the seven runs of training sets reached 0.839 and for the test sets, it was 0.821. Hence, there exists an “optimism” of 0.018 in the mean area under the ROCs for the training and testing splits, and as a result, the internally validated (or optimism corrected) c-statistics is estimated as 0.818.  To provide more robust evidence of validity, **external (in fact: spatial) validation** (also called generalizability) was conducted with a new sample of 478 patients admitted. The c-statistics in the external sample decreased to 0.809 (a decrease of 0.027) which is slightly more than results from internal validation (a decrease of 0.018).  However, **both internal and external validations** confirm the superiority of our proposal over the cur-rent approaches in terms of discrimination power and stability.  **Comparisons with other approaches.**  The comparison results are summarized in Table7 and Fig.4. **As shown, the proposal works better than other alternatives in all predictive criteria.** |
| **Costs** | |
| **Time to completion** | NI The risk prediction model works real-time. This may imply that result can be available immediately. |
| **Specific input data required** | N. The method directly incorporates patient’s history of readmission and risk factors changes over time.  The study is limited to administrative data (that are regularly available to all health plans) and it does not have laboratory test results and vital signs such as haemoglobin or serum level at discharge, which may affect the risk of unnecessary readmission. |
| **Specific training required** | NI |
| **Who completed the tool?** | |
| Likely study authors. | |
| **When/where/how tool was/can be completed? (for example: at discharge/at admission, in hospital/in nursing home, retrospective/prospective)** | |
| Likely in hospital. Likely prospective use. | |
| **Language** | **Tool can be seen/accessed in:** |
| English | The algorithm, phase-type survival forest, is described in text (pages 3-7) and in box “Algorithm 1” (page 7): ([59](#_ENREF_59)) |

1. **RAFT (Reducing Avoidable Facility Transfers) model**

**Table 33. RAFT (Reducing Avoidable Facility Transfers) model**

| **Tool name (reported in (**[**60**](#_ENREF_60)**))** | |
| --- | --- |
| **RAFT (Reducing Avoidable Facility Transfers) model** | |
| **Concept/components covered** | |
| RAFT consists of the following components:  1. Small team of providers who manage longitudinal care and  after-hours call. 2. Systematic elicitation of advance care plans including acute  care preferences 3. Increased engagement of the provider during an acute care  event 4. Case Review. | |
| **What the tool measures/does** | |
| Provides sort of plan for action, with 3 distinct phases: before, during and after acute event. | |
| **Objectivity** | |
| **Process** | Y. Visualisation of the RAFT intervention in Figure 1 is self-explaining. Further intervention details are also described in the study. |
| **Evaluation** | Y. For example, component on informed decision making: “providers are aware of Advance Directives during acute event and make recommendations in this context.” We think that making recommendations in the context of AD could involve subjective judgement. |
| **Interpretation** | The RAFT model is an intervention with 3 distinct sections: before, during and after acute event. Unlike other interventions, the focus was not on whether the transfer was clinically indicated, but rather what the team might have reasonably and safely done differently to change the outcome. |
| **Reliability** | |
| **Inter-rater** | NI |
| **Intra-rater** | NI |
| **Validity** | |
| **Convergent** | NI |
| **Costs** | |
| **Time to completion** | NI |
| **Specific input data required** | Y. As part of an intervention, completion of advance care plans and Provider Orders for Life Sustaining Treatment (POLST) form were required. |
| **Specific training required** | Y. As part of an intervention, a nurse-led education session was held with all nursing staff to explain the benefits of engaging the provider early. |
| **Who completed the tool?** | |
| Physicians, nurse practitioners, physician´s assistant. | |
| **When/where/how tool was/can be completed? (for example: at discharge/at admission, in hospital/in nursing home, retrospective/prospective)** | |
| In SNF, prospective use. | |
| **Language** | **Tool can be seen/accessed in:** |
| English | Visual representation of the RAFT intervention can be seen in Figure 1: ([60](#_ENREF_60)) |

1. **Ottawa Heart Failure Risk Scale (OHFRS)**

**Table 34. Ottawa Heart Failure Risk Scale (OHFRS)**

| **Tool name (reported in (**[**61**](#_ENREF_61)**))** | |
| --- | --- |
| **Ottawa Heart Failure Risk Scale (OHFRS)** | |
| **Concept/components covered** | |
| 10 criteria grouped into 3 sections: initial assessment, investigations, walk test after ED treatment. | |
| **What the tool measures/does** | |
| Identifies ED patients with acute heart failure at high risk for serious adverse events. | |
| **Objectivity** | |
| **Process** | Y. Self-explaining. |
| **Evaluation** | N. Tool is composed of simple bedside variables.  Clinical and laboratory results from the electronic patient records including standardized variables from the history, clinical examination, routine laboratory values, cardiac, chest x-ray, and initial and repeat ECG, and a 3 minute walk test. |
| **Interpretation** | Y. Scoring system, max. score is 15.  Provides a table on how to convert a total score into %-risk or risk category (low, medium, high, very high). Presented in Figure 1 of the study. |
| **Reliability** | |
| **Inter-rater** | NI |
| **Intra-rater** | NI |
| **Validity** | |
| **Convergent** | **Performance of OHFRS Without NT-proBNP**  The most useful threshold scores appear to be >1 (optimal sensitivity) and >2 (decreased admissions). Compared to actual practice, using an admission threshold of OHFRS score >1 would have increased sensitivity (71.8% vs.91.8%) but increased admissions (57.2% vs. 77.6%). Using a threshold >2 would have led to a similar sensitivity (71.8% vs. 71.2%) but reduced admission rates (57.2% vs. 48.3%).  **Performance of OHFRS With NT-proBNP**  Compared to actual practice, using an admission threshold of OHFRS score >1 would have significantly increased sensitivity (69.8% vs. 95.8%) while increasing admissions (60.8% vs. 88.0%). Using a threshold >2 would have led to better sensitivity (69.8% vs.79.8%) but with similar admission rates (60.8% vs.63.0%).  **Conclusions**  Compared to current practice, an OHFRS score threshold of >1 would significantly improve sensitivity but would require more admissions. Alternately, a threshold of >2 would offer similar sensitivity to current practice but reduce admissions. NT-proBNP values were available for about 60% of patients and their incorporation into the OHFRS scores led to better sensitivity. |
| **Costs** | |
| **Time to completion** | NI |
| **Specific input data required** | Y. For example, ECG, Urea, Serum, Troponin. |
| **Specific training required** | Y. When tool was completed by supervised residents in emergency medicine training programs. |
| **Who completed the tool?** | |
| ED physicians or supervised residents in emergency medicine training programs, who were trained by means of a 1-hour practical session. | |
| **When/where/how tool was/can be completed? (for example: at discharge/at admission, in hospital/in nursing home, retrospective/prospective)** | |
| In ED, prospective use. | |
| **Language** | **Tool can be seen/accessed in:** |
| English | Figure 1: ([61](#_ENREF_61)) |

1. **Ottawa COPD (chronic obstructive pulmonary disease) Risk Scale (OCRS)**

**Table 35. The Ottawa COPD (chronic obstructive pulmonary disease) Risk Scale (OCRS)**

| **Tool name (reported in (**[**62**](#_ENREF_62)**))** | |
| --- | --- |
| **The Ottawa COPD (chronic obstructive pulmonary disease) Risk Scale (OCRS)** | |
| **Concept/components covered** | |
| 10 items grouped into 3 sections: initial assessment, investigations, re-assessment after ED treatment. | |
| **What the tool measures/does** | |
| Identifies ED patients with acute COPD who are at high risk for short-term serious outcomes. | |
| **Objectivity** | |
| **Process** | Y. Self-explaining. |
| **Evaluation** | N. A scale comprising 10 items from history, physical examination and bedside tests. |
| **Interpretation** | Y. Scoring system, max. score is 16.  Has a table for conversion of a total score to a %-risk and risk class (low, medium, high, very high) |
| **Reliability** | |
| **Inter-rater** | NI |
| **Intra-rater** | NI |
| **Validity** | |
| **Convergent** | Choosing total point scores of 1 or 2 as the threshold for admission would be associated with sensitivities for a short-term serious outcome of 79.3% or 71.9%, respectively. These theoretical admission thresholds would lead to absolute admission rates of 56.6% or 47.9%, respectively, compared with the observed admission rate of 45.0% at the study hospitals.  **Conclusions**  Compared with current practice, an OCRS score threshold of 1 or more would increase sensitivity by 50% but would require 25% more admissions. Alternately, a threshold of 2 or more would improve sensitivity by 38% while leading to only a slight increase in admissions. |
| **Costs** | |
| **Time to completion** | NI |
| **Specific input data required** | Y. For example, ECG, Chest X-ray, Haemoglobin, Urea, Serum |
| **Specific training required** | Y. Attending physicians and residents in emergency medicine were trained locally. |
| **Who completed the tool?** | |
| Physicians, residents in emergency medicine. | |
| **When/where/how tool was/can be completed? (for example: at discharge/at admission, in hospital/in nursing home, retrospective/prospective)** | |
| In ED. Likely prospective use. | |
| **Language** | **Tool can be seen/accessed in:** |
| English | Figure 1: ([62](#_ENREF_62)) |

1. **Comprehensive Geriatric Assessment (was reported as a component in 2 studies/interventions)**

**Table 36. 1. A&E-based geriatric admission-avoidance system: Triage and Rapid Elderly Assessment Team (TREAT) (with focus on CGA only).**

**2. INTERCARE nurse-led model (with focus on CGA only)**

| **Tool name (reported in (**[**63-65**](#_ENREF_63)**))** | |
| --- | --- |
| 1. A&E-based geriatric admission-avoidance system: Triage and Rapid Elderly Assessment Team (TREAT) **(with focus on CGA only) (**[**63**](#_ENREF_63)**).** 2. INTERCARE nurse-led model **(with focus on CGA only) (**[**64**](#_ENREF_64)**,** [**65**](#_ENREF_65)**).** | |
| **Concept/components covered** | |
| The CGA includes the following dimensions: Physical dimension, Functional dimension, Social dimension, Economic dimension, Mental dimension. ([65](#_ENREF_65)) | |
| **What the tool measures/does** | |
| Helps to identify unknown geriatric syndromes or problems, thus helping to manage patients. ([66](#_ENREF_66)) | |
| **Objectivity** | |
| **Process** | NI  CGA is rather a set of components or dimensions that guide an assessment. For each dimension/component a specific assessment instrument is required. ([65](#_ENREF_65), [66](#_ENREF_66)).  The INTERCARE nurse collaborates with the leadership and/or interprofessional team to discuss and define which assessment instrument they work with, for each of the 5 CGA dimensions in their institution. ([65](#_ENREF_65)) |
| **Evaluation** | NI |
| **Interpretation** | NI |
| **Reliability** | |
| **Inter-rater** | NI |
| **Intra-rater** | NI |
| **Validity** | |
| **Convergent** | NI |
| **Costs** | |
| **Time to completion** | Y. CGA is a time-consuming process, which may be problematic in ED. It takes around 25 min. to complete. ([66](#_ENREF_66)). |
| **Specific input data required** | Y. For CGA it is required to use another assessment instruments to assess each CGA dimension/component. ([65](#_ENREF_65), [66](#_ENREF_66)) |
| **Specific training required** | Y. The INTERCARE nurse provides information and guidance to the care team about the 5 different dimensions and can suggest how each dimension can be assessed and evaluated ([65](#_ENREF_65)). |
| **Who completed the tool?** | |
| Any care staff can be involved in the 5 dimensions of the CGA, corresponding to their degree of training and experience. ([65](#_ENREF_65)).  Physicians ([66](#_ENREF_66)).  Consultant geriatrician ([63](#_ENREF_63)). | |
| **When/where/how tool was/can be completed? (for example: at discharge/at admission, in hospital/in nursing home, retrospective/prospective)** | |
| In NHs, Comprehensive geriatric assessment of residents initiated by INTERCARE nurses when a change in condition was observed ([65](#_ENREF_65)).  Accident and Emergency (A&E) department of a hospital, the consultant geriatrician selected patients for TREAT from the A&E admissions, performing a CGA in A&E for these patients ([63](#_ENREF_63)).  In ED, CGA is useful for identifying unknown geriatric syndromes or problems, in order to help ED physicians manage such patients ([66](#_ENREF_66)).  Likely prospective use. | |
| **Language** | **Tool can be seen/accessed in:** |
| English ([65](#_ENREF_65), [66](#_ENREF_66)) | CGA dimensions are presented in Table S1: ([65](#_ENREF_65))  CGA components are presented in Table 1: ([66](#_ENREF_66)) |

1. **Standardised chart review method and its sub-types**

**Table 37. Standardised chart review method with ORIGINAL trigger tool**

| **Tool name (reported in (**[**67**](#_ENREF_67)**))** | |
| --- | --- |
| **Standardised chart review method with ORIGINAL trigger tool** | |
| **Concept/components covered** | |
| Has 3 steps: (i) data abstraction, (ii) screening for triggered events using the newly developed trigger tool, screening for non-triggered events using two screening questions and (iii) adjudication in terms of ADE causality and contribution to hospital admission (DRA).  Original trigger tool from (ii) includes 26 triggers classified into 3 categories: diagnoses triggers - 13  laboratory values triggers - 10  other triggers - 3 | |
| **What the tool measures/does** | |
| Identifies drug related hospital admissions (DRAs). | |
| **Objectivity** | |
| **Process** | Y. Self-explaining. Further details on the standardised chart review method is given in the study.  For each trigger, a list of potentially causative drugs or potential causes for drug underuse was provided. A trigger was positive when the situation and a potential causative drug (or drug lacking in case of underuse) were both present. The whole process followed by the adjudication committee was considered to be the gold standard to define an ADE and a DRA.  The adjudication committee recorded the following data in the Electronic Case Report Forms: presence/absence of(a) each of the 26 triggers, associated ADE for each positive trigger (using WHO causality criteria [20]), medication involved when an ADE was recorded, associated DRA (main reason or contributory reason) and medications involved in each DRA; (b) non-triggered events, associated ADE, associated DRA and type of event(s) and medication(s)involved. Finally, each hospitalisation classified as DRA was also classified by type: adverse drug reactions, overuse, misuse or underuse. Each adjudicated hospitalisation could have more than one trigger, ADE or non-triggered event. |
| **Evaluation** | Y. For example, in one of the triggers (for heart failure exacerbation) there is a question that may involve some subjective judgement when answering it: “use of any drugs (*provided list of drugs*) that could precipitate heart failure exacerbation?”. |
| **Interpretation** | Y. In the three-step standardised chart review procedure it is written that “DRA judged to be due to a medication error is considered preventable”. |
| **Reliability** | |
| **Inter-rater** | NI |
| **Intra-rater** | NI |
| **Validity** | |
| **Convergent** | The overall PPV value [CI 95%] of the tool for detecting DRAs was 0.66 [0.62–0.69].  The tool’s overall PPV value for detecting preventable DRAs was 0.28 [0.25–0.32].  The tool’s overall PPV value for detecting ADEs was 0.87 [0.84–0.89]. |
| **Costs** | |
| **Time to completion** | Y. The trigger tool remains time-consuming. |
| **Specific input data required** | Y. In the three-step standardised chart review procedure a section on “data abstraction” lists necessary data, including but not limited to: laboratory values, medication lists, previous falls, past medical history, and more. |
| **Specific training required** | NI |
| **Who completed the tool?** | |
| Experienced pharmacists and physicians. | |
| **When/where/how tool was/can be completed? (for example: at discharge/at admission, in hospital/in nursing home, retrospective/prospective)** | |
| In medical centres, retrospective using patient records. | |
| **Language** | **Tool can be seen/accessed in:** |
| English | Appendix 1: ([67](#_ENREF_67)) |

**Table 38. Standardised chart review method with REVISED trigger tool**

| **Tool name (reported in (**[**67**](#_ENREF_67)**))** | |
| --- | --- |
| **Standardised chart review method with REVISED trigger tool.** | |
| **Concept/components covered** | |
| Has 3 steps: (i) data abstraction, (ii) screening for triggered events using the newly developed trigger tool, screening for non-triggered events using two screening questions and (iii) adjudication in terms of ADE causality and contribution to hospital admission (DRA).  REVISED trigger tool consists of 21 triggers (step ii of method): diagnoses triggers – 16, laboratory values triggers – 3, other triggers – 2. | |
| **What the tool measures/does** | |
| Identifies drug-related hospital admissions (DRAs). | |
| **Objectivity** | |
| **Process** | Y. Self-explaining. Further details on the standardised chart review method is given in the study.  Logic how the three-step standardised chart review with the revised trigger tool works is the same as in the three-step standardised chart review with the original trigger tool. |
| **Evaluation** | Y. For example, in one of the triggers (for heart failure exacerbation) there is a question that may involve some subjective judgement when answering it: “use of any drugs (*provided list of drugs*) that could precipitate heart failure exacerbation?”. |
| **Interpretation** | Y. In the three-step standardised chart review procedure it is written that “DRA judged to be due to a medication error is considered preventable”. |
| **Reliability** | |
| **Inter-rater** | NI |
| **Intra-rater** | NI |
| **Validity** | |
| **Convergent** | NI |
| **Costs** | |
| **Time to completion** | Y. The revised trigger tool seems to be less time-consuming as compared to the original trigger tool.  The trigger tool remains time-consuming, so we also developed a user-friendly version that could help clinicians to identify DRAs more effectively. |
| **Specific input data required** | Y. In the three-step standardised chart review procedure, step 1 on “data abstraction” lists necessary data, including but not limited to: laboratory values, medication lists, previous falls, past medical history, and more. |
| **Specific training required** | NI |
| **Who completed the tool?** | |
| Revised trigger tool was just proposed based on study results. However, it can be also used by clinicians. | |
| **When/where/how tool was/can be completed? (for example: at discharge/at admission, in hospital/in nursing home, retrospective/prospective)** | |
| In medical centres, retrospective using patient records. | |
| **Language** | **Tool can be seen/accessed in:** |
| English | Revised trigger tool is shown in Table 3. Clinical adaptation of the revised trigger tool is shown in Table 4. For comparison of Original and Revised trigger tools, see Appendix 6. ([67](#_ENREF_67)) |

1. **Tool on appropriate referrals by Bermejo Higuera et al.**

**Table 39. Tool on appropriate referrals by Bermejo Higuera et al**

| **Tool name (reported in (**[**68**](#_ENREF_68)**) )** | |
| --- | --- |
| **Tool on appropriate referrals by Bermejo Higuera et al** | |
| **Concept/components covered** | |
| Tool has 3 criteria relating to duration of observation in a hospital, whether a patient needed to see a specialist and/or needed special diagnostic tests, whether a patient needed special treatment. | |
| **What the tool measures/does** | |
| Identifies appropriate or relevant hospital admissions. | |
| **Objectivity** | |
| **Process** | Y. Self explaining. |
| **Evaluation** | N. Does not seem to include a subjective judgement.  Tool has 3 criteria relating to duration of observation in a hospital, whether a patient needed to see a specialist and/or needed special diagnostic tests, whether a patient needed special treatment. |
| **Interpretation** | Y. Referrals are considered appropriate or relevant to be those that meet one of the following criteria:  1. the patient was admitted to hospital or stayed in observation for more than 24 hours  2. the patient had to be seen by a specialist and/or required diagnostic tests not available in the nursing home  3. the patient required treatment not available in the nursing home |
| **Reliability** | |
| **Inter-rater** | NI |
| **Intra-rater** | NI |
| **Validity** | |
| **Convergent** | NI |
| **Costs** | |
| **Time to completion** | NI |
| **Specific input data required** | N. Since data required relates to duration of observation in a hospital, whether a patient needed to see a specialist and/or needed special diagnostic tests, whether a patient needed special treatment. |
| **Specific training required** | NI |
| **Who completed the tool?** | |
| Likely study authors. | |
| **When/where/how tool was/can be completed? (for example: at discharge/at admission, in hospital/in nursing home, retrospective/prospective)** | |
| In NH, retrospective using medical records. | |
| **Language** | **Tool can be seen/accessed in:** |
| Study was in Spanish language. | Page 2, in text: ([68](#_ENREF_68)) |

1. **Tool by Codde et al. List of Exclusion criteria and potentially avoidable reasons for emergency department (ED) presentation**

**Table 40. Tool by Codde et al. List of Exclusion criteria and potentially avoidable reasons for emergency department (ED) presentation**

| **Tool name (reported in (**[**69**](#_ENREF_69)**))** | |
| --- | --- |
| **Tool by Codde et al. List of Exclusion criteria and potentially avoidable reasons for emergency department (ED) presentation** | |
| **Concept/components covered** | |
| 1. Exclusion criteria (11) for potentially avoidable ED presentations. Criteria justifying ED presentation.  2. Criteria (11) for potentially avoidable ED presentations. Criteria NOT justifying ED presentation (I.e. criteria indicating potentially avoidable ED presentations). | |
| **What the tool measures/does** | |
| Provides list of exclusion criteria and potentially avoidable reasons for emergency department (ED) presentation. | |
| **Objectivity** | |
| **Process** | Y. Self-explaining. |
| **Evaluation** | Y. Some elements may seem subjective in judgement. For example, assessing for “significant neurological changes” or “increasing confusion with no signs of UTI”. |
| **Interpretation** | Y. To determine avoidable presentations, we developed a list of 10 exclusion criteria based on the clinical presentation or other factors such as family request for transfer (see Table 1).  From Table 1 we noticed/understood the following:  1. They presented exclusion criteria (we counted 11) for potentially avoidable ED presentations. We understood these criteria as justifying ED presentation.  2. They also listed criteria (we counted 11) for potentially avoidable ED presentations. We understood these criteria as NOT justifying ED presentation (I.e. criteria indicating potentially avoidable ED presentations). |
| **Reliability** | |
| **Inter-rater** | Analysis of the interrater reliability of a subset of 54 cases demonstrated agreement of intraclass correlation coefficient 0.414 (95% CI 0.277–0.560) between the four raters. In 28 of 54 cases, all raters assessed the case identically. From these 54 cases, the emergency medicine specialist identified 54% as potentially avoidable, the GP 70%, and the two ED nurses 71% and 74%. This equates to a range of 24–33% of all transfers being avoidable. |
| **Intra-rater** | NI |
| **Validity** | |
| **Convergent** | NI |
| **Costs** | |
| **Time to completion** | NI |
| **Specific input data required** | N. Data can be taken from medical records.  In this study authors gathered data from ED Information System and Hospital Morbidity Data System. |
| **Specific training required** | NI |
| **Who completed the tool?** | |
| Single experienced ED nurse. | |
| **When/where/how tool was/can be completed? (for example: at discharge/at admission, in hospital/in nursing home, retrospective/prospective)** | |
| Retrospective using medical records.  Authors analysed data from a single tertiary hospital ED patient database. | |
| **Language** | **Tool can be seen/accessed in:** |
| English | Table 1: ([69](#_ENREF_69)) |

1. **A prediction rule to identify low-risk patients with community-acquired pneumonia (Pneumonia Severity Index, PSI)**

**Table 41. A prediction rule to identify low-risk patients with community-acquired pneumonia**

| **Tool name (reported in (**[**31**](#_ENREF_31)**,** [**70**](#_ENREF_70)**))** | |
| --- | --- |
| **A prediction rule to identify low-risk patients with community-acquired pneumonia (Pneumonia Severity Index, PSI)** | |
| **Concept/components covered** | |
| Covers items such as demographic factors, health conditions, physical examination findings, nursing home residency, laboratory and radiographic findings. | |
| **What the tool measures/does** | |
| Identifies low-risk patients with community-acquired pneumonia (CAP). | |
| **Objectivity** | |
| **Process** | Y. Self –explaining. |
| **Evaluation** | N. Required data relates to demographic factors, coexisting illnesses, physical examination findings, laboratory and radiographic findings. |
| **Interpretation** | Y. A tool has 2 steps which are administered to assign a patient to one of risk classes out of 5 in total:  1. Step 1 is a flow chart form, used to identify whether a patient can be assigned to risk class I. Figure 1.  2. Step 2 is a table form, a point based system to identify whether a patient can be assigned to risk classes from II to V. Table 2.  Points calculated from table 2 are then classified into classes from II to V according to table 3.  Transformation of points into risk classes according to table 3:  II (<=70)  III (71-90)  IV (91-130)  V (>130) |
| **Reliability** | |
| **Inter-rater** | NI |
| **Intra-rater** | NI |
| **Validity** | |
| **Convergent** | There was no significant difference (P = 0.15) in the area under the receiver-operating-characteristic curves between the MedisGroups derivation cohort (0.84) and the MedisGroups validation cohort (0.83). Although the area under the curve was significantly greater in the Pneumonia PORT cohort (0.89) than in either of the MedisGroups cohorts (P=0.001), the absolute differences in area were minimal. |
| **Costs** | |
| **Time to completion** | NI |
| **Specific input data required** | Y. For example, laboratory and radiographic data. |
| **Specific training required** | NI |
| **Who completed the tool?** | |
| Can be used by physicians. | |
| **When/where/how tool was/can be completed? (for example: at discharge/at admission, in hospital/in nursing home, retrospective/prospective)** | |
| Authors validated the tool using a database and also using data from prospective cohort study (patient followed prospectively) using chart review and patient interviews (prospective tool use). Can be used at the time of patient presentation to hospital, but also can be used in outpatients. Likely prospective and retrospective use. | |
| **Language** | **Tool can be seen/accessed in:** |
| English | Figure 1, table 2 & 3: ([70](#_ENREF_70)) |

1. **Tool by Gozalo et al. on Three types of transitions that were classified as being potentially burdensome**

**Table 42. Tool by Gozalo et al. on three types of transitions that were classified as being potentially burdensome.**

| **Tool name (reported in (**[**71**](#_ENREF_71)**))** | |
| --- | --- |
| **Tool by Gozalo et al. on three types of transitions that were classified as being potentially burdensome.** | |
| **Concept/components covered** | |
| Three types of transitions were classified as being potentially burdensome. These types relate to end-of-life transitions, lack of continuity of nursing home facilities, multiple hospitalizations. | |
| **What the tool measures/does** | |
| Classifies transitions as being potentially burdensome. | |
| **Objectivity** | |
| **Process** | Y. Self-explaining. |
| **Evaluation** | Y. For example, one element refers to “any transfer in the last 3 days of life” may involve subjective judgement (i.e. time to death) |
| **Interpretation** | Y. 3 types of transitions classified as potentially burdensome:  1. any transfer in the last 3 days of life,  2. a lack of continuity of nursing home facilities before and after a hospitalization in the last 90 days of life (i.e., going from nursing home A to the hospital and then to nursing home B),  3. and multiple hospitalizations in the last 90 days of life.  A burdensome-transition score was created (range, 0 to 3) on the basis of the occurrence of any event in each category during the last 90 days of life. |
| **Reliability** | |
| **Inter-rater** | NI |
| **Intra-rater** | NI |
| **Validity** | |
| **Convergent** | NI |
| **Costs** | |
| **Time to completion** | NI |
| **Specific input data required** | NI  Specific data is not required by the instrument. However,  access to some data (for example data on ADLs, cognitive impairment) may be needed to make necessary judgements. |
| **Specific training required** | NI |
| **Who completed the tool?** | |
| Likely study authors. | |
| **When/where/how tool was/can be completed? (for example: at discharge/at admission, in hospital/in nursing home, retrospective/prospective)** | |
| Retrospective using MDS data on all NS residents in the USA and Medicare claims data. | |
| **Language** | **Tool can be seen/accessed in:** |
| English | Page 2 in text: ([71](#_ENREF_71)) |

1. **Tool by Ong et al., on time to death as an indication of the inappropriateness of admissions**

**Table 43. Tool by Ong et al., on time to death as an indication of the inappropriateness of admissions**

| **Tool name (reported in (**[**72**](#_ENREF_72)**))** | |
| --- | --- |
| **Tool by Ong et al., on time to death as an indication of the inappropriateness of admissions** | |
| **Concept/components covered** | |
| Assessment based on time to death. | |
| **What the tool measures/does** | |
| Indicates appropriateness of hospitalizations based on time to death. | |
| **Objectivity** | |
| **Process** | Y. Self-explaining. |
| **Evaluation** | Y. Assessment is based on time to death, and judgement on time to death may be subjective. |
| **Interpretation** | Y. Depending on time to death, a patient could be managed either in a care home or in an acute medical setting.  Deaths were then categorized as incurable or likely to be manageable in the care home (deaths occurring within 3 days of index admission), potentially predictable (within 4–7 days of admission) and likely to be appropriate for acute medical intervention (death after 7 days). |
| **Reliability** | |
| **Inter-rater** | NI |
| **Intra-rater** | NI |
| **Validity** | |
| **Convergent** | NI |
| **Costs** | |
| **Time to completion** | NI |
| **Specific input data required** | Y. Specific data is not required by the instrument.  However, access to some data may be required to make necessary judgements.  This study was retrospective and they recorded total number of admissions, cause of death, and the number of days to death from the index admission. |
| **Specific training required** | NI |
| **Who completed the tool?** | |
| Likely study authors. | |
| **When/where/how tool was/can be completed? (for example: at discharge/at admission, in hospital/in nursing home, retrospective/prospective)** | |
| Retrospective use by reviewing hospital admissions from care homes to a hospital. | |
| **Language** | **Tool can be seen/accessed in:** |
| English | Page 2 in text: ([72](#_ENREF_72)) |

1. **Modified Early Warning Score (MEWS)**

**Table 44. Modified Early Warning Score (MEWS).**

| **Tool name (reported in (**[**73**](#_ENREF_73)**))** | |
| --- | --- |
| **Modified Early Warning Score (MEWS).** | |
| **Concept/components covered** | |
| Systolic blood pressure, pulse rate, respiratory rate, temperature and AVPU score ([73](#_ENREF_73)) | |
| **What the tool measures/does** | |
| Identifies patients at risk of deterioration who require increased levels of care in the HDU or ICU. ([73](#_ENREF_73)) | |
| **Objectivity** | |
| **Process** | Y. Self-explaining. |
| **Evaluation** | N. Assessment is made based on systolic blood pressure, pulse rate, respiratory rate, temperature and AVPU score ([73](#_ENREF_73)) |
| **Interpretation** | Y. Scoring system.  Total score of 4 or more was considered as a ward alert by nurses.  Patients with a MEWS of 3 or  4 in the preoperative evaluation or at operating room discharge were transferred to HDU, whereas a MEWS score of 5 or more was considered a criteria for ICU admission. In case of a total MEWS of 3 calculated only on neurological status (subscore = 3) the patient was admitted to the ICU, as well as in case of a patient with a total MEWS of 2 (made by subscore of 2 in heart rate or by subscore of 2 in respiratory rate) in which the HDU was chosen instead of the surgical ward. ([74](#_ENREF_74))  Total points calculated, then MEWS crore of 5 or more is judged as a “critical score”. ([73](#_ENREF_73)) |
| **Reliability** | |
| **Inter-rater** | NI |
| **Intra-rater** | NI |
| **Validity** | |
| **Convergent** | NI |
| **Costs** | |
| **Time to completion** | NI |
| **Specific input data required** | N. The instrument is for bedside evaluation based on 5 physiological parameters. ([74](#_ENREF_74)) |
| **Specific training required** | Y. Following the internal protocol for emergency and not-scheduled surgical patients, nurses of the surgical ward and HDU were trained in MEWS collection during the patient´s routine evaluation. ([74](#_ENREF_74))  Appropriate training was provided to nursing staff. ([73](#_ENREF_73)) |
| **Who completed the tool?** | |
| Anaesthetist, surgical ward nurses. ([74](#_ENREF_74))  Nursing staff. ([73](#_ENREF_73)) | |
| **When/where/how tool was/can be completed? (for example: at discharge/at admission, in hospital/in nursing home, retrospective/prospective)** | |
| Prospective use, tool used before surgical procedure and before discharge from operating room. Likely in surgical ward or HDU. ([74](#_ENREF_74))  At admission / hospital / prospective ([73](#_ENREF_73)) | |
| **Language** | **Tool can be seen/accessed in:** |
| English | Table 1: ([73](#_ENREF_73)) |

1. **The 80+ score**

**Table 45. The 80+ score**

| **Tool name (reported in (**[**37**](#_ENREF_37)**))** | |
| --- | --- |
| **The 80+ score** | |
| **Concept/components covered** | |
| The 80+ score includes the following 7 items:  Estimated glomerular filtration rate (eGFR), Level of social support, Pulmonary disease (asthma or chronic obstruction pulmonary disease), Malignant disease, Prescription of a drug for peptic ulcer or gastro-oesophageal reflux disease, Prescription of an opioid drug, Prescription of an antidepressant drug (except tricyclic antidepressant). ([75](#_ENREF_75)) | |
| **What the tool measures/does** | |
| Prediction of risk of rehospitalisation and mortality in hospital patients. ([75](#_ENREF_75)) | |
| **Objectivity** | |
| **Process** | Y. Self-explaining. ([75](#_ENREF_75)) |
| **Evaluation** | N. Clinical and drug variables were included. See table 1. ([75](#_ENREF_75)) |
| **Interpretation** | Y. Scoring system. Table 4 provides estimate of risk for each point total. ([75](#_ENREF_75)) |
| **Reliability** | |
| **Inter-rater** | NI |
| **Intra-rater** | NI |
| **Validity** | |
| **Convergent** | The goodness-of-fit of the 80+ score was good and is illustrated in figure 1. This was confirmed by the Grønnesby-Borgan test (p=0.49).  The 80+ score demonstrated a satisfying discriminatory ability of the outcome, with a C-statistic of 0.715 (figure 2). The optimism was 0.001, rendering an optimism-corrected C-statistic of 0.714 for the 80+ score. This means that a patient with an event (revisit to the hospital or death) had a 71% probability of being given a higher risk score than a patient with no event. When tested in the control group only, the 80+ score had a C-statistic of 0.71, which is similar to the value for the group as a whole.  The 80+ risks core has a higher discriminatory ability for risk of rehospitalisation and mortality than most other prediction models of today ([75](#_ENREF_75)).  Discrimination: The 80+ score had the lowest AUC (0.506).  Calibration: (Hosmer-Lemeshow χ2 test): 80+ score: X^2^ = 7.89, degree of freedom = 8, p-value = 0.44.  ([37](#_ENREF_37)) |
| **Costs** | |
| **Time to completion** | Y. A simple and user-friendly point score system like this can quickly and easily identify high-risk patients. ([75](#_ENREF_75)) |
| **Specific input data required** | Y. For example, renal function (estimated glomerular filtration rate (eGFR)). ([75](#_ENREF_75)) |
| **Specific training required** | NI However, seems that no specific training required, as the instrument is simple and user-friendly. ([75](#_ENREF_75)) |
| **Who completed the tool?** | |
| Intended for use by clinicians. ([75](#_ENREF_75)) | |
| **When/where/how tool was/can be completed? (for example: at discharge/at admission, in hospital/in nursing home, retrospective/prospective)** | |
| Retrospective using medical records. Likely in hospital. ([37](#_ENREF_37)).  Likely prospective use. ([75](#_ENREF_75)). | |
| **Language** | **Tool can be seen/accessed in:** |
| English ([75](#_ENREF_75)) | Tool with its scoring system is in Table 1, and a conversion table (estimate of risk for each point total) is in Table 4: ([75](#_ENREF_75)) |

1. **The TRST**

**Table 46. The TRST**

| **Tool name (reported in (**[**37**](#_ENREF_37)**))** | |
| --- | --- |
| **The TRST** | |
| **Concept/components covered** | |
| The TRST is a 5 item clinical prediction: History of cognitive impairment, Difficulty walking/transferring or recent falls, Taking five or more medications, ED use in previous 30 days or hospitalisation in previous 90 days, RN (registered nurse) professional recommendation. ([76](#_ENREF_76)) | |
| **What the tool measures/does** | |
| Identifies emergency department (ED) patients at risk for ED revisits, hospitalization, or nursing home (NH) placement within 30 and 120 days following ED discharge. ([76](#_ENREF_76)) | |
| **Objectivity** | |
| **Process** | Y. Self-explaining. ([76](#_ENREF_76)) |
| **Evaluation** | Y. The element “RN professional recommendation” is explained in the study as: “Emergency department (ED) nurse (RN) concern for elder abuse/neglect, substance abuse, medication noncompliance, problems meeting instrumental activities of daily living, or other.”  This element of an instrument is based on nurse´s recommendation, which may involve subjective judgement. ([76](#_ENREF_76)) |
| **Interpretation** | Y. A 5-item tool. Risk factors were assessed categorically (yes/no for the items cognitive impairment, difficulty walking/transferring, and professional recommendation; and yes/no/unable to determine for the remaining items: polypharmacy, and recent ED use or hospitalization). The number of risk factors present were summed.  Subjects were considered to be a high-risk cohort, a priori, if they had cognitive impairment alone, or the presence of two or more TRST risk factors. ([76](#_ENREF_76)) |
| **Reliability** | |
| **Inter-rater** | For the purpose of studying reliability, TRST surveys were completed for 37 patients by two different surveyors. There was one discrepancy out of 222 questions (37 screens using the six TRST items). Kappa was 1.0 for all items except for a single discrepancy regarding professional recommendation (kappa = 0.94). ([76](#_ENREF_76)) |
| **Intra-rater** | NI |
| **Validity** | |
| **Convergent** | Logistic regression modelling revealed that a summed, un-weighted five-item TRST (sans lives alone), with a cut-off score of 2, produced nearly as good a fit (AUC=0.64) in predicting the composite outcome. For the individual outcome hospitalization, the AUC was 0.72 at 30 days and 0.65 at 120 days.  Three hundred ten subjects had both APN and TRST classifications of high- or low-risk recorded. The APN and TRST classifications had 70% agreement (kappa, 0.38; 95% CI¼0.28 to 0.49).  Sensitivity and specificity of the TRST to predict 30-and 120-day composite outcomes are shown in Table3.  **30-day Composite Outcome**  **Sensitivity** (cut-off > 2)**:** 0.64  **Specificity** (cut-off > 2)**:** 0.63  **120-day Composite Outcome**  **Sensitivity** (cut-off > 2)**:** 0.55  **Specificity** (cut-off > 2)**:** 0.66  The TRST cut-off score was designed to be fairly sensitive in detecting at-risk elders and was initially weighted toward cognitive impairment. However, 99% of high-risk elders were positive for at least two TRST items and a simplified cut-off score of 2 would detect this group.  ([76](#_ENREF_76))  **Discrimination:** AUC for TRST was 0.589 (0.524–0.654)  **Calibration** (Hosmer-Lemeshow χ2 test.): TRST χ2= 3.44; degrees of freedom= 4; p-value= 0.49  **Sensitivity** (cut-off > 2)**:** 0.37 (0.31–0.43)  **Specificity** (cut-off > 2)**:** 0.74 (0.68–0.80)  **PPV** (cut-off > 2): 0.59 (0.50–0.67)  **NPV** (cut-off > 2): 0.54 (0.48–0.60)  ([37](#_ENREF_37)) |
| **Costs** | |
| **Time to completion** | Y. 1 to 2 minutes to complete. ([76](#_ENREF_76)) |
| **Specific input data required** | N. Does not seem to require specific data. ([76](#_ENREF_76)) |
| **Specific training required** | Y. May require some educational sessions, however, minimal instructions are sufficient. ([37](#_ENREF_37), [76](#_ENREF_76))  The TRST was standardized and educational sessions were conducted for all nursing personnel prior to initiating the study. Nursing personnel need minimal instruction in its use. ([76](#_ENREF_76)) |
| **Who completed the tool?** | |
| ED staff nurses. ([76](#_ENREF_76)) | |
| **When/where/how tool was/can be completed? (for example: at discharge/at admission, in hospital/in nursing home, retrospective/prospective)** | |
| In ED, prospective use. ([76](#_ENREF_76)) | |
| **Language** | **Tool can be seen/accessed in:** |
| English ([76](#_ENREF_76)) | Figure 1: ([76](#_ENREF_76)) |

1. **ERA index**

**Table 47. ERA index**

| **Tool name (reported in (**[**43**](#_ENREF_43)**))** | |
| --- | --- |
| **ERA index** | |
| **Concept/components covered** | |
| Age, marital status, length of hospital stay, history of diabetes, heart disease, stroke, chronic obstructive pulmonary disease (COPD), neoplasia and dementia. ([77](#_ENREF_77)) | |
| **What the tool measures/does** | |
| Risk of visits, emergency room visits/hospital admissions and hospital stay. ([43](#_ENREF_43)) | |
| **Objectivity** | |
| **Process** | Y. Self-explaining. ([77](#_ENREF_77)) |
| **Evaluation** | N. Does not involve subjective judgement. ([77](#_ENREF_77)) |
| **Interpretation** | Y. Total score calculated based on 10 components. The range of the score varies from -7 to 32, patients with a score ≥ 16 have the highest risk of visits, emergency room visits/hospital admissions and hospital stay. ([43](#_ENREF_43))  Table 3 guides how to transform total score to relative risk of emergency room visits and hospital visits. ([77](#_ENREF_77)) |
| **Reliability** | |
| **Inter-rater** | NI |
| **Intra-rater** | NI |
| **Validity** | |
| **Convergent** | The area under the curve (AUC) for the primary outcome of combined hospitalizations and emergency room visits was 0.678. For hospital visits only, the AUC was 0.705. For emergency room visits only, the AUC was 0.640. ([77](#_ENREF_77)) |
| **Costs** | |
| **Time to completion** | NI |
| **Specific input data required** | N. No specific data required. ([77](#_ENREF_77)) |
| **Specific training required** | NI |
| **Who completed the tool?** | |
| Likely study authors. ([43](#_ENREF_43)) | |
| **When/where/how tool was/can be completed? (for example: at discharge/at admission, in hospital/in nursing home, retrospective/prospective)** | |
| no data / likely in hospital / retrospectively ([43](#_ENREF_43)) | |
| **Language** | **Tool can be seen/accessed in:** |
| English ([77](#_ENREF_77)) | Table 1. Table 3 guides how to transform total score to relative risk of emergency room visits and hospital visits: ([77](#_ENREF_77)) |

1. **Risk prediction model for PARAs**

**Table 48. Risk prediction model for PARAs**

| **Tool name (reported in (**[**44**](#_ENREF_44)**))** | |
| --- | --- |
| **Risk prediction model for PARAs** | |
| **Concept/components covered** | |
| At least one hospitalisation in the 12 months preceding the index admission, cancer diagnosis, blood sodium <135 mmol/, Charlson score >1, length of stay >11 days, and the prescription of at least 15 different medications during the stay. | |
| **What the tool measures/does** | |
| Assessment of 30 day risk of PARA. | |
| **Objectivity** | |
| **Process** | Y. Self-explaining. |
| **Evaluation** | N. Does not involve subjective judgement. |
| **Interpretation** | Y. Scoring system, ranges from 0 to 10.5 points. Total points then transformed into three risk categories of PARA: low (0–1.5 points), intermediate (1.5–5 points), high (>5 points). |
| **Reliability** | |
| **Inter-rater** | NI |
| **Intra-rater** | NI |
| **Validity** | |
| **Convergent** | Area under the ROC curve of 0.696. The p-value of the Hosmer-Lemeshow goodness-of-fit statistic was 0.69. The C statistic for the level of risk was at 0.65. |
| **Costs** | |
| **Time to completion** | NI |
| **Specific input data required** | Y. Requires specific data, laboratory analysis for blood sodium level. |
| **Specific training required** | NI |
| **Who completed the tool?** | |
| Likely study authors. | |
| **When/where/how tool was/can be completed? (for example: at discharge/at admission, in hospital/in nursing home, retrospective/prospective)** | |
| At discharge (last data available before discharge, extracted from hospital data system) / in hospital / retrospective | |
| **Language** | **Tool can be seen/accessed in:** |
| English | Table 3 shows components with associated points. Rule to transform total points to risk classes is shown in text on page 4: ([44](#_ENREF_44)) |

1. Huckfeldt PJ, Kane RL, Yang Z, Engstrom G, Tappen R, Rojido C, et al. Degree of Implementation of the Interventions to Reduce Acute Care Transfers (INTERACT) Quality Improvement Program Associated with Number of Hospitalizations. J Am Geriatr Soc. 2018;66(9):1830-7.

2. Kane RL, Huckfeldt P, Tappen R, Engstrom G, Rojido C, Newman D, et al. Effects of an Intervention to Reduce Hospitalizations From Nursing Homes: A Randomized Implementation Trial of the INTERACT Program. JAMA Internal Medicine. 2017;177(9):1257-64.

3. Tappen RM, Newman D, Huckfeldt P, Yang Z, Engstrom G, Wolf DG, et al. Evaluation of Nursing Facility Resident Safety During Implementation of the INTERACT Quality Improvement Program. J Am Med Dir Assoc. 2018;19(10):907-13.e1.

4. Ouslander JG, Bonner A, Herndon L, Shutes J. The Interventions to Reduce Acute Care Transfers (INTERACT) quality improvement program: an overview for medical directors and primary care clinicians in long term care. J Am Med Dir Assoc. 2014;15(3):162-70.

5. Ouslander JG, Perloe M, Givens JH, Kluge L, Rutland T, Lamb G. Reducing potentially avoidable hospitalizations of nursing home residents: results of a pilot quality improvement project. J Am Med Dir Assoc. 2009;10(9):644-52.

6. Ouslander JG, Reyes B, Shutes J, Engstrom G, Diaz S. INTERACT tools library 2023 [<https://pathway-interact.com/interact-tools/interact-tools-library/.2023> Accessed 20.06.2023

7. Lamb G, Tappen R, Diaz S, Herndon L, Ouslander JG. Avoidability of hospital transfers of nursing home residents: perspectives of frontline staff. J Am Geriatr Soc. 2011;59(9):1665-72.

8. Ouslander JG, Lamb G, Tappen R, Herndon L, Diaz S, Roos BA, et al. Interventions to reduce hospitalizations from nursing homes: evaluation of the INTERACT II collaborative quality improvement project. J Am Geriatr Soc. 2011;59(4):745-53.

9. Popejoy LL, Vogelsmeier AA, Alexander GL, Galambos CM, Crecelius CA, Ge B, et al. Analyzing Hospital Transfers Using INTERACT Acute Care Transfer Tools: Lessons from MOQI. J Am Geriatr Soc. 2019;67(9):1953-9.

10. Unroe KT, Nazir A, Holtz LR, Maurer H, Miller E, Hickman SE, et al. The Optimizing Patient Transfers, Impacting Medical Quality, andImproving Symptoms:Transforming Institutional Care approach: preliminary data from the implementation of a Centers for Medicare and Medicaid Services nursing facility demonstration project. J Am Geriatr Soc. 2015;63(1):165-9.

11. Hullick CJ, Hall AE, Conway JF, Hewitt JM, Darcy LF, Barker RT, et al. Reducing Hospital Transfers from Aged Care Facilities: A Large-Scale Stepped Wedge Evaluation. J Am Geriatr Soc. 2021;69(1):201-9.

12. Hullick C, Conway J, Higgins I, Hewitt J, Dilworth S, Holliday E, et al. Emergency department transfers and hospital admissions from residential aged care facilities: a controlled pre-post design study. BMC Geriatr. 2016;16:102.

13. (ACE) ACES. Clinical guidlines 2023 [<https://ace.hnehealth.nsw.gov.au/guidelines.2023> Accessed 22.06.2023

14. (ACE) ACES. Aged Care Emergency generic manual 2020 [<https://hunterprimarycare.com.au/wp-content/uploads/2021/09/ACE-Manual-Generic_FINAL_20210908.pdf.2020> Accessed 22.06.2023

15. (ACE) ACES. Education resources 2023 [<https://ace.hnehealth.nsw.gov.au/education_resources.2023> Accessed 22.06.2023

16. Sampson EL, Feast A, Blighe A, Froggatt K, Hunter R, Marston L, et al. Pilot cluster randomised trial of an evidence-based intervention to reduce avoidable hospital admissions in nursing home residents (Better Health in Residents of Care Homes with Nursing-BHiRCH-NH Study). BMJ open. 2020;10(12):e040732.

17. Downs M, Blighe A, Carpenter R, Feast A, Froggatt K, Gordon S, et al. A complex intervention to reduce avoidable hospital admissions in nursing homes: a research programme including the BHiRCH-NH pilot cluster RCT. Programme Grants for Applied Research. 2021.

18. Murna Downs AB, Robin Carpenter, Alexandra Feast, Katherine Froggatt, Sally Gordon, Rachael Hunter, Liz Jones, Natalia Lago, Brendan McCormack, Louise Marston, Shirley Nurock, Monica Panca, Helen Permain, Catherine Powell, Greta Rait, Louise Robinson, Barbara Woodward-Carlton, John Wood, John Young and Elizabeth Sampson. A complex intervention to reduce avoidable hospital admissions in nursing homes: a research programme including the BHiRCH-NH pilot cluster RCT. Programme Grants Appl Res. 2021;9(2).

19. Tappen RM, Worch SM, Newman DO, Hain D. Evaluation of a Novel Decision Guide "Go to the Hospital or Stay Here?" for Nursing Home Residents and Families: A Randomized Trial. Res Gerontol Nurs. 2020;13(6):309-19.

20. Tappen RM. Decision Guide n.d. [<http://www.decisionguide.org/.n.d>. Accessed 23.06.2023

21. Selker HP, Beshansky JR, Griffith JL, Aufderheide TP, Ballin DS, Bernard SA, et al. Use of the acute cardiac ischemia time-insensitive predictive instrument (ACI-TIPI) to assist with triage of patients with chest pain or other symptoms suggestive of acute cardiac ischemia. A multicenter, controlled clinical trial. Annals of Internal Medicine. 1998;129(11):845-55.

22. Baré ML, Prat A, Lledo L, Asenjo MA, Salleras L. Appropriateness of admissions and hospitalization days in an acute-care teaching hospital. Rev Epidemiol Sante Publique. 1995;43(4):328-36.

23. Abdoulhadi D, Chevalet P, Moret L, Fix MH, Gégu M, Jaulin P, et al. [Appropriateness of direct admissions to acute care geriatric unit for nursing home patients: an adaptation of the AEPf GRID]. Geriatr Psychol Neuropsychiatr Vieil. 2015;13(1):15-21.

24. Davido A, Nicoulet I, Levy A, Lang T. Appropriateness of admission in an emergency department: reliability of assessment and causes of failure. Qual Assur Health Care. 1991;3(4):227-34.

25. Duflos C, Antoun S, Loirat P, DiPalma M, Minvielle E. Identification of appropriate and potentially avoidable emergency department referrals in a tertiary cancer care center. Support Care Cancer. 2017;25(8):2377-85.

26. Pérès K, Rainfray M, Perrié N, Emeriau JP, Chêne G, Barberger-Gateau P. [Incidence, risk factors and adequation of early readmission among the elderly]. Rev Epidemiol Sante Publique. 2002;50(2):109-19.

27. Almeida A, Serrasqueiro Z, Rogerio A. [Review of the utilization of a Portuguese public hospital]. Acta Med Port. 2006;19(5):381-5.

28. Attena F, Agozzino E, Troisi MR, Granito C, Del Prete U. Appropriateness of admission and hospitalization days in a specialist hospital. Ann Ig. 2001;13(2):121-7.

29. Soria-Aledo V, Carrillo-Alcaraz A, Flores-Pastor B, Moreno-Egea A, Carrasco-Prats M, Aguayo-Albasini JL. Reduction in inappropriate hospital use based on analysis of the causes. BMC Health Serv Res. 2012;12:361.

30. Velasco Díaz L, García Ríos S, Oterino de la Fuente D, Suárez García F, Diego Roza S, Fernández Alonso R. [Impact on hospital days of care due to unnecessary emergency admissions]. Rev Esp Salud Publica. 2005;79(5):541-9.

31. Migliorati PL, Boccoli E, Bracci LS, Sestini P, Melani AS. A survey on hospitalised community-acquired pneumonia in Italy. Monaldi Arch Chest Dis. 2006;65(2):82-8.

32. Angelillo IF, Ricciardi G, Nante N, Boccia A, Bianco A, La Torre G, et al. Appropriateness of hospital utilisation in Italy. Public Health. 2000;114(1):9-14.

33. Aliberti S, Ramirez J, Cosentini R, Brambilla AM, Zanaboni AM, Rossetti V, et al. Low CURB-65 is of limited value in deciding discharge of patients with community-acquired pneumonia. Respir Med. 2011;105(11):1732-8.

34. Karmakar G, Wilsher M. Use of the 'CURB 65' score in hospital practice. Intern Med J. 2010;40(12):828-32.

35. Lim WS, Eerden MMvd, Laing R, Boersma WG, Karalus N, Town GI, et al. Defining community acquired pneumonia severity on presentation to hospital: an international derivation and validation study. Thorax. 2003;58(5):377-82.

36. Arendts G, Etherton-Beer C, Jones R, Bullow K, MacDonald E, Dumas S, et al. Use of a risk nomogram to predict emergency department reattendance in older people after discharge: a validation study. Intern Emerg Med. 2015;10(4):481-7.

37. Schwab C, Le Moigne A, Fernandez C, Durieux P, Sabatier B, Korb-Savoldelli V. External validation of the 80+ score and comparison with three clinical scores identifying patients at least 75 years old at risk of unplanned readmission within 30 days after discharge. Swiss Med Wkly. 2018;148:w14624.

38. Lázaro Cebas A, Caro Teller JM, García Muñoz C, González Gómez C, Ferrari Piquero JM, Lumbreras Bermejo C, et al. Intervention by a clinical pharmacist carried out at discharge of elderly patients admitted to the internal medicine department: influence on readmissions and costs. BMC Health Serv Res. 2022;22(1):167.

39. Aubert CE, Schnipper JL, Williams MV, Robinson EJ, Zimlichman E, Vasilevskis EE, et al. Simplification of the HOSPITAL score for predicting 30-day readmissions. BMJ Qual Saf. 2017;26(10):799-805.

40. Burke RE, Schnipper JL, Williams MV, Robinson EJ, Vasilevskis EE, Kripalani S, et al. The HOSPITAL Score Predicts Potentially Preventable 30-Day Readmissions in Conditions Targeted by the Hospital Readmissions Reduction Program. Med Care. 2017;55(3):285-90.

41. Donzé J, Aujesky D, Williams D, Schnipper JL. Potentially avoidable 30-day hospital readmissions in medical patients: derivation and validation of a prediction model. JAMA Intern Med. 2013;173(8):632-8.

42. Donzé JD, Williams MV, Robinson EJ, Zimlichman E, Aujesky D, Vasilevskis EE, et al. International Validity of the HOSPITAL Score to Predict 30-Day Potentially Avoidable Hospital Readmissions. JAMA Intern Med. 2016;176(4):496-502.

43. De Giorgi A, Boari B, Tiseo R, López-Soto PJ, Signani F, Gallerani M, et al. Hospital readmissions to internal medicine departments: a higher risk for females? Eur Rev Med Pharmacol Sci. 2016;20(21):4557-64.

44. Uhlmann M, Lécureux E, Griesser AC, Duong HD, Lamy O. Prediction of potentially avoidable readmission risk in a division of general internal medicine. Swiss Med Wkly. 2017;147:w14470.

45. Baig M, Zhang E, Robinson R, Ullah E, Whitakker R. Evaluation of Patients at Risk of Hospital Readmission (PARR) and LACE Risk Score for New Zealand Context...Health Informatics Conference, Sydney Australia, 2018. Studies in Health Technology & Informatics. 2018;252:21-6.

46. Walraven Cv, Dhalla IA, Bell C, Etchells E, Stiell IG, Zarnke K, et al. Derivation and validation of an index to predict early death or unplanned readmission after discharge from hospital to the community. Canadian Medical Association Journal. 2010;182(6):551-7.

47. Teh R, Janus E. Identifying and targeting patients with predicted 30-day hospital readmissions using the revised LACE index score and early postdischarge intervention. Int J Evid Based Healthc. 2018;16(3):174-81.

48. Higi L, Lisibach A, Beeler PE, Lutters M, Blanc AL, Burden AM, et al. External validation of the PAR-Risk Score to assess potentially avoidable hospital readmission risk in internal medicine patients. PLoS One. 2021;16(11):e0259864.

49. Horey DE, Street AF, Sands AF. Acceptability and feasibility of end-of-life care pathways in Australian residential aged care facilities. Med J Aust. 2012;197(2):106-9.

50. (BSPCC) BSPCC. Residential aged care end of life care pathway 2023 [<https://metrosouth.health.qld.gov.au/raceolcp.2023> Accessed 02.07.2023

51. Inzitari M, Gual N, Roig T, Colprim D, Pérez-Bocanegra C, San-José A, et al. Geriatric Screening Tools to Select Older Adults Susceptible for Direct Transfer From the Emergency Department to Subacute Intermediate-Care Hospitalization. J Am Med Dir Assoc. 2015;16(10):837-41.

52. McCusker J, Bellavance F, Cardin S, Belzile E, Verdon J. Prediction of hospital utilization among elderly patients during the 6 months after an emergency department visit. Annals of Emergency Medicine. 2000;36(5):438-45.

53. Di Bari M, Salvi F, Roberts AT, Balzi D, Lorenzetti B, Morichi V, et al. Prognostic stratification of elderly patients in the emergency department: a comparison between the "Identification of Seniors at Risk" and the "Silver Code". The journals of gerontology Series A, Biological sciences and medical sciences. 2012;67(5):544-50.

54. Walter LC, Brand RJ, Counsell SR, Palmer RM, Landefeld CS, Fortinsky RH, et al. Development and Validation of a Prognostic Index for 1-Year Mortality in Older Adults After Hospitalization. JAMA. 2001;285(23):2987-94.

55. Johnston JJ, Longman JM, Ewald DP, Rolfe MI, Diez Alvarez S, Gilliland AHB, et al. Validity of a tool designed to assess the preventability of potentially preventable hospitalizations for chronic conditions. Fam Pract. 2020;37(3):390-4.

56. Oddone EZ, Weinberger M, Horner M, Mengel C, Goldstein F, Ginier P, et al. Classifying general medicine readmissions. Are they preventable? Veterans Affairs Cooperative Studies in Health Services Group on Primary Care and Hospital Readmissions. J Gen Intern Med. 1996;11(10):597-607.

57. Saliba D, Kington R, Buchanan J, Bell R, Wang M, Lee M, et al. Appropriateness of the decision to transfer nursing facility residents to the hospital. J Am Geriatr Soc. 2000;48(2):154-63.

58. Patel R, Clancy R, Crowther E, Vannahme M, Pullyblank A. A rectal bleeding algorithm can successfully reduce emergency admissions. Colorectal Dis. 2014;16(5):377-81.

59. Shams I, Ajorlou S, Yang K. A predictive analytics approach to reducing 30-day avoidable readmissions among patients with heart failure, acute myocardial infarction, pneumonia, or COPD. Health Care Manag Sci. 2015;18(1):19-34.

60. Stadler DS, Oliver BJ, Raymond JG, Routzhan GF, Flaherty EA, Stahl JE, et al. Reducing Avoidable Facility Transfers (RAFT): Outcomes of a Team Model to Minimize Unwarranted Emergency Care at Skilled Nursing Facilities. J Am Med Dir Assoc. 2019;20(8):929-34.

61. Stiell IG, Perry JJ, Clement CM, Brison RJ, Rowe BH, Aaron SD, et al. Prospective and Explicit Clinical Validation of the Ottawa Heart Failure Risk Scale, With and Without Use of Quantitative NT-pro BNP. Academic Emergency Medicine. 2017;24(3):316-27.

62. Stiell IG, Perry JJ, Clement CM, Brison RJ, Rowe BH, Aaron SD, et al. Clinical validation of a risk scale for serious outcomes among patients with chronic obstructive pulmonary disease managed in the emergency department. Cmaj. 2018;190(48):E1406-e13.

63. Wright PN, Tan G, Iliffe S, Lee D. The impact of a new emergency admission avoidance system for older people on length of stay and same-day discharges. Age Ageing. 2014;43(1):116-21.

64. Zúñiga F, De Geest S, Guerbaai RA, Basinska K, Nicca D, Kressig RW, et al. Strengthening Geriatric Expertise in Swiss Nursing Homes: INTERCARE Implementation Study Protocol. J Am Geriatr Soc. 2019;67(10):2145-50.

65. Zúñiga F, Guerbaai R-A, de Geest S, Popejoy LL, Bartakova J, Denhaerynck K, et al. Positive effect of the INTERCARE nurse-led model on reducing nursing home transfers: A nonrandomized stepped-wedge design. Journal of the American Geriatrics Society. 2022;70(5):1546-57.

66. Graf CE, Zekry D, Giannelli S, Michel J-P, Chevalley T. Efficiency and applicability of comprehensive geriatric assessment in the Emergency Department: a systematic review. Aging Clinical and Experimental Research. 2011;23(4):244-54.

67. Zerah L, Henrard S, Thevelin S, Feller M, Meyer-Masseti C, Knol W, et al. Performance of a trigger tool for detecting drug-related hospital admissions in older people: analysis from the OPERAM trial. Age & Ageing. 2022;51(1):1-13.

68. Bermejo Higuera Jc CMRD-AHEMACVDM. Derivaciones al Servicio de Urgencias del hospital en una población de ancianos residentes. Estudio retrospectivo sobre sus causas y adecuación. [Hospital transfers from a population of elderly residents. A retrospective study about the causes and suitability]. Gerokomos. 2010;21(3):114-7.

69. Codde J, Frankel J, Arendts G, Babich P. Quantification of the proportion of transfers from residential aged care facilities to the emergency department that could be avoided through improved primary care services. Australas J Ageing. 2010;29(4):167-71.

70. Fine MJ, Auble TE, Yealy DM, Hanusa BH, Weissfeld LA, Singer DE, et al. A prediction rule to identify low-risk patients with community-acquired pneumonia. N Engl J Med. 1997;336(4):243-50.

71. Gozalo P, Teno JM, Mitchell SL, Skinner J, Bynum J, Tyler D, et al. End-of-Life Transitions among Nursing Home Residents with Cognitive Issues. New England Journal of Medicine. 2011;365(13):1212-21.

72. Ong AC, Sabanathan K, Potter JF, Myint PK. High mortality of older patients admitted to hospital from care homes and insight into potential interventions to reduce hospital admissions from care homes: the Norfolk experience. Arch Gerontol Geriatr. 2011;53(3):316-9.

73. Subbe CP, Kruger M, Rutherford P, Gemmel L. Validation of a modified Early Warning Score in medical admissions. QJM: An International Journal of Medicine. 2001;94(10):521-6.

74. Peris A, Zagli G, Maccarrone N, Batacchi S, Cammelli R, Cecchi A, et al. The use of Modified Early Warning Score may help anesthesists in postoperative level of care selection in emergency abdominal surgery. Minerva Anestesiol. 2012;78(9):1034-8.

75. Alassaad A, Melhus H, Hammarlund-Udenaes M, Bertilsson M, Gillespie U, Sundström J. A tool for prediction of risk of rehospitalisation and mortality in the hospitalised elderly: secondary analysis of clinical trial data. BMJ open. 2015;5(2):e007259.

76. Meldon SW, Mion LC, Palmer RM, Drew BL, Connor JT, Lewicki LJ, et al. A brief risk-stratification tool to predict repeat emergency department visits and hospitalizations in older patients discharged from the emergency department. Academic emergency medicine : official journal of the Society for Academic Emergency Medicine. 2003;10(3):224-32.

77. Crane SJ, Tung EE, Hanson GJ, Cha S, Chaudhry R, Takahashi PY. Use of an electronic administrative database to identify older community dwelling adults at high-risk for hospitalization or emergency department visits: The elders risk assessment index. BMC Health Services Research. 2010;10(1):338.
