# Supplementary figures and images for: Assessment tools addressing avoidable care transitions in older adults: a systematic literature review
Source: Eur Geriatr Med. 2024 Nov 29;15(6):1587–601. doi: 10.1007/s41999-024-01106-7 (PMC11632047; doi:10.1007/s41999-024-01106-7)

**Supplementary file 4: RoB table**


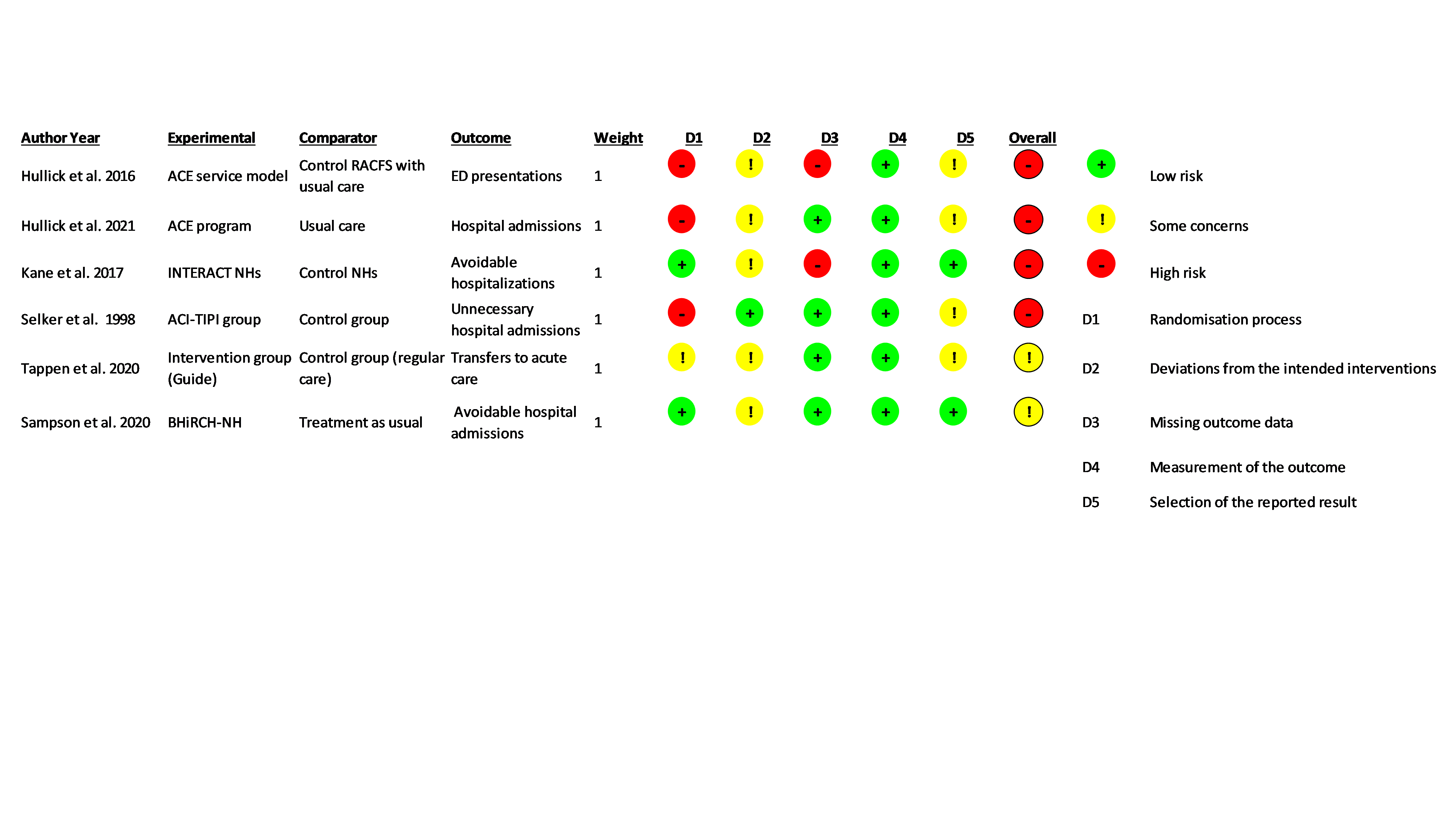

Supplement: Supplementary file 4 — Supplementary file4 (DOCX 180 KB) [file 41999_2024_1106_MOESM4_ESM.docx]
