# Supplementary material for: Assessment tools addressing avoidable care transitions in older adults: a systematic literature review
Source: Eur Geriatr Med. 2024 Nov 29;15(6):1587–601. doi: 10.1007/s41999-024-01106-7 (PMC11632047; doi:10.1007/s41999-024-01106-7)
Supplement: Supplementary file 5 — Supplementary file5 (DOCX 52 KB) [file 41999_2024_1106_MOESM5_ESM.docx]

**Supplementary file 5: List of excluded studies**

| **#** | **Studies Category 1** | **Reason for exclusion** |
| --- | --- | --- |
| 1 | Lewin G, Jiwa M. Prevention of avoidable hospital admissions of older people living at home in Western Australia: a pilot randomized control trial. <https://trialsearchwhoint/Trial2aspx?TrialID=ACTRN12613000907741>. 2013. | Study registration record. |
| 2 | Adam L, Moutzouri E, Baumgartner C, Loewe AL, Feller M, M'Rabet-Bensalah K, et al. Rationale and design of OPtimising thERapy to prevent Avoidable hospital admissions in Multimorbid older people (OPERAM): a cluster randomised controlled trial. BMJ Open. 2019;9(6):e026769. | Study protocol. |
| 3 | Alonso Bouzón C, Petidier Torregrossa R, Marín Larraín PP, Rodriguez Mañas L. [Effectiveness of reevaluation of admission of patients with poor functional status]. Rev Esp Geriatr Gerontol. 2010;45(1):19-21. | Wrong study design.  No assessment tool reported. |
| 4 | Bourke R, Rice C, McMahon G, Cunningham C, Kenny RA, Briggs R. 304 ED-FASU: A Novel 'Front Door' Multidisciplinary Service Assessing Patients with Falls and Syncope in the Emergency Department...67th Annual & Scientific Meeting of the Irish Gerontological Society, Innovation, Advances and Excellence in Ageing, 26–28 September 2019, Cork, Ireland. Age & Ageing. 2019;48:iii17-iii65. | Poster presentation.  No assessment tool reported. |
| 5 | Brühmann BA, Reese C, Kaier K, Ott M, Maurer C, Kunert S, et al. A complex health services intervention to improve medical care in long-term care homes: study protocol of the controlled coordinated medical care (CoCare) study. BMC Health Serv Res. 2019;19(1):332. | Study protocol. |
| 6 | Carter HE, Lee XJ, Farrington A, Shield C, Graves N, Cyarto EV, et al. A stepped-wedge randomised controlled trial assessing the implementation, effectiveness and cost-consequences of the EDDIE+ hospital avoidance program in 12 residential aged care homes: study protocol. BMC Geriatrics. 2021;21(1):347‐. | Study protocol. |
| 7 | Connolly MJ, Boyd M, Broad JB, Kerse N, Lumley T, Whitehead N, et al. The Aged Residential Care Healthcare Utilization Study (ARCHUS): a multidisciplinary, cluster randomized controlled trial designed to reduce acute avoidable hospitalizations from long-term care facilities. J Am Med Dir Assoc. 2015;16(1):49-55. | No assessment tool reported. |
| 8 | Connolly MJ, Broad JB, Boyd M, Kerse N, Foster S, Lumley T, et al. Randomised controlled trial of packaged “evidenced” interventions for reducing hospitalisations from residential aged care (RAC): first results from the ARCHUS study. European Geriatric Medicine. 2013;4:S171‐. | Poster presentation.  No full text. |
| 9 | Connolly MJ, Broad JB, Boyd M, Kerse N, Foster S, Lumley T, et al. CLUSTER-RANDOMISED CONTROLLED TRIAL (RCT) OF A MULTIDISCIPLINARY INTERVENTION PACKAGE FOR REDUCING DISEASE-SPECIFIC HOSPITALISATIONS FROM LONG TERM CARE (LTC). Age & Ageing. 2014;43(suppl_2):ii19-ii. | Poster presentation.  No assessment tool reported. |
| 10 | Crowley EK, Sallevelt BTGM, Huibers CJA, Murphy KD, Spruit M, Shen Z, et al. Intervention protocol: OPtimising thERapy to prevent avoidable hospital Admission in the Multi-morbid elderly (OPERAM): a structured medication review with support of a computerised decision support system. BMC Health Services Research. 2020;20(1):1-12. | Study protocol. |
| 11 | Foster SJ, Boyd M, Broad JB, Whitehead N, Kerse N, Lumley T, et al. Aged Residential Care Health Utilisation Study (ARCHUS): a randomised controlled trial to reduce acute hospitalisations from residential aged care. BMC Geriatr. 2012;12:54. | Study protocol. |
| 12 | Fournaise A, Lauridsen JT, Bech M, Wiil UK, Rasmussen JB, Kidholm K, et al. Prevention of AcuTe admIssioN algorithm (PATINA): study protocol of a stepped wedge randomized controlled trial. BMC Geriatr. 2021;21(1):146. | Study protocol. |
| 13 | Freund T, Peters-Klimm F, Rochon J, Mahler C, Gensichen J, Erler A, et al. Primary care practice-based care management for chronically ill patients (PraCMan): study protocol for a cluster randomized controlled trial. Trials. 2011;12:163. | Study protocol. |
| 14 | Hullick C, Conway J, Hall A, Murdoch W, Cole J, Hewitt J, et al. Video-telehealth to support clinical assessment and management of acutely unwell older people in Residential Aged Care: a pre-post intervention study. BMC Geriatr. 2022;22(1):40. | No assessment tool reported. |
| 15 | Downs M. The better health in residents in care homes study: Pilot study. <https://trialsearchwhoint/Trial2aspx?TrialID=ISRCTN74109734>. 2017. | Study registration record. |
| 16 | Blighe A. Feasibility study to reduce avoidable hospitalisations and promote Better Health in Residents in Care Homes (BHiRCH). <https://trialsearchwhoint/Trial2aspx?TrialID=ISRCTN86811077>. 2017. | Study registration record. |
| 17 | Lamppu P, Finne-Soveri H, Laakkonen ML, Laurila J, Pitkala K. Educating nursing home staff in palliative care to improve end-oflife care and to reduce burdensome hospitalisations: baseline findings and feasibility of a randomised, controlled trial. European Geriatric Medicine. 2018;9:S42‐. | Poster presentation.  No full text. |
| 18 | Mendes A. Multimorbidity, optimising treatment and preventing hospital admissions in older people...Blum M, Sallevelt B, Spinewine A, et al. Optimizing Therapy to Prevent Avoidable Hospital Admissions in Multimorbid Older Adults (OPERAM): cluster randomised controlled trial. BMJ. 2021; 374: n1585. Journal of Prescribing Practice. 2021;3(9):344-5. | Wrong study design.  No assessment tool reported. |
| 19 | Noel K, Yagudayev S, Messina C, Schoenfeld E, Hou W, Kelly G. Tele-transitions of care. A 12-month, parallel-group, superiority randomized controlled trial protocol, evaluating the use of telehealth versus standard transitions of care in the prevention of avoidable hospital readmissions. Contemp Clin Trials Commun. 2018;12:9-16. | No assessment tool reported. |
| 20 | Huibers CJAL. OPtimising thERapy to prevent Avoidable hospital admissions in the Multimorbid elderly. <https://trialsearchwhoint/Trial2aspx?TrialID=NTR6012>. 2016. | Study registration record. |
| 21 | Ouslander JG, Reyes B, Yang Z, Engstrom G, Tappen R, Newman D, et al. Nursing home performance in a trial to reduce hospitalizations: Implications for future trials. J Am Geriatr Soc. 2021;69(8):2316-26. | Wrong study design.  No assessment tool reported. |
| 22 | Palacholla RS, Fischer NC, Agboola S, Nikolova-Simons M, Odametey S, Golas SB, et al. Evaluating the Impact of a Web-Based Risk Assessment System (CareSage) and Tailored Interventions on Health Care Utilization: Protocol for a Randomized Controlled Trial. JMIR Res Protoc. 2018;7(5):e10045. | Study protocol. |
| 23 | Piotrowski A, Meyer M, Burkholder I, Renaud D, Müller MA, Lehr T, et al. Effect of an interprofessional care concept on the hospitalization of nursing home residents: study protocol for a cluster-randomized controlled trial. Trials. 2020;21(1):411. | Study protocol. |
| 24 | Sampson EL, Feast A, Blighe A, Froggatt K, Hunter R, Marston L, et al. Evidence-based intervention to reduce avoidable hospital admissions in care home residents (the Better Health in Residents in Care Homes (BHiRCH) study): protocol for a pilot cluster randomised trial. BMJ Open. 2019;9(5):e026510. | Study protocol. |
| 25 | Jia H, Chuang H, Wu SS, Wang X, Chumbler NR. Long-term effect of home telehealth services on preventable hospitalization use. Journal of Rehabilitation Research & Development. 2009;46(5):557-66. | No assessment tool reported. |
| 26 | Stop and watch tool reduces avoidable hospital readmissions. Remington Report. 2011;19(3):36-8. | No full text. |
| 27 | Vogelsmeier A, Popejoy L, Kist S, Shumate S, Pritchett A, Mueller J, et al. Reducing Avoidable Hospitalizations for Nursing Home Residents: Role of the Missouri Quality Initiative Intervention Support Team. J Nurs Care Qual. 2020;35(1):1-5. | Wrong study design.  No assessment tool reported. |

| **#** | **Studies Category 2** | **Reason for exclusion** |
| --- | --- | --- |
| 1 | Stop and watch tool reduces avoidable hospital readmissions. Remington Report. 2011;19(3):36-8. | No full text. |
| 2 | Lewin G, Jiwa M. Prevention of avoidable hospital admissions of older people living at home in Western Australia: a pilot randomized control trial. <https://trialsearchwhoint/Trial2aspx?TrialID=ACTRN12613000907741>. 2013. | Study registration record. |
| 3 | Allen BR, Simpson GG, Zeinali I, Freitas JT, Chapa JJ, Rawson LJ, et al. Incorporation of the HEART Score Into a Low-risk Chest Pain Pathway to Safely Decrease Admissions. Crit Pathw Cardiol. 2018;17(4):184-90. | Wrong study population. |
| 4 | Alonso Bouzón C, Petidier Torregrossa R, Marín Larraín PP, Rodriguez Mañas L. [Effectiveness of reevaluation of admission of patients with poor functional status]. Rev Esp Geriatr Gerontol. 2010;45(1):19-21. | No assessment tool reported. |
| 5 | Avigni N, Ippoliti M, Muccinelli M, Kubbajeh M, Zanotti C, Tonioli M, et al. [Chest pain in the emergency department: benefits of a management model modified from the ANMCO-SIMEU recommendations]. G Ital Cardiol (Rome). 2011;12(5):365-73. | No assessment tool reported. |
| 6 | Bonner A, Tappen R, Herndon L, Ouslander J. The INTERACT Institute: Observations on Dissemination of the INTERACT Quality Improvement Program Using Certified INTERACT Trainers. Gerontologist. 2015;55(6):1050-7. | Wrong study design. |
| 7 | Briggs R, McDonough A, Ellis G, Bennett K, O'Neill D, Robinson D. Comprehensive Geriatric Assessment for community‐dwelling, high‐risk, frail, older people. Cochrane Database of Systematic Reviews. 2022(5). | Wrong study design. |
| 8 | Broman KK, Poulose BK, Phillips SE, Ehrenfeld JM, Sharp KW, Pierce RA, et al. Unnecessary Transfers for Acute Surgical Care: Who and Why? Am Surg. 2016;82(8):672-8. | No assessment tool reported. |
| 9 | Buitrago I, Seidl KL, Gingold DB, Marcozzi D. Analysis of Readmissions in a Mobile Integrated Health Transitional Care Program Using Root Cause Analysis and Common Cause Analysis. Journal for Healthcare Quality: Promoting Excellence in Healthcare. 2022;44(3):169-77. | No assessment tool reported. |
| 10 | Carnessale G, Staniscia T, Matarrese D, Seccia G, Schioppa F, Di Giovanni P, et al. [Appropriateness of hospitalization in the teaching hospital of Chieti using the P.R.U.O. approach]. Ann Ig. 2003;15(2):117-22. | Wrong study population. |
| 11 | Collins G. Implementation of an integrated, multidisciplinary team model of screening, assessment and intervention for the elderly population in a medical assessment unit in Galway University Hospital, Ireland. International Journal of Integrated Care (IJIC). 2017;17:1-. | Poster presentation. |
| 12 | Downs M, Blighe A, Carpenter R, Feast A, Froggatt K, Gordon S, et al. Programme Grants for Applied Research. A complex intervention to reduce avoidable hospital admissions in nursing homes: a research programme including the BHiRCH-NH pilot cluster RCT. 2021. | Excluded here, because already included in Category 1. |
| 13 | Engel L, Hwang K, Panayiotou A, Watts JJ, Mihalopoulos C, Temple J, et al. Identifying patterns of potentially preventable hospitalisations in people living with dementia. BMC Health Serv Res. 2022;22(1):794. | No assessment tool reported. |
| 14 | Falvey JR, Burke RE, Levy CR, Gustavson AM, Price L, Forster JE, et al. Impaired Physical Performance Predicts Hospitalization Risk for Participants in the Program of All-Inclusive Care for the Elderly. Phys Ther. 2019;99(1):28-36. | No assessment tool reported. |
| 15 | Fluitman KS, van Galen LS, Merten H, Rombach SM, Brabrand M, Cooksley T, et al. Exploring the preventable causes of unplanned readmissions using root cause analysis: Coordination of care is the weakest link. Eur J Intern Med. 2016;30:18-24. | No assessment tool reported. |
| 16 | Franks S. Transitional Care to Reduce 30-day Heart Failure Readmissions Among the Long-Term Care Elderly Population...28th Annual Scientific Session, June 2-6, 2017, Baltimore, Maryland. Nursing Research. 2016;65(2):E37-E8. | Poster presentation. |
| 17 | Fried RA, Main DS, Calonge BN. Appropriateness of hospital use by family physicians. J Am Board Fam Pract. 1994;7(3):229-35. | Wrong study population. |
| 18 | Gujral S, Bell CR, Dare L, Smith PJ, Persad RA, Gujral S, et al. A prospective evaluation of the management of acute pyelonephritis in adults referred to urologists. International Journal of Clinical Practice. 2003;57(3):238-40. | No assessment tool reported.  Wrong study population. |
| 19 | Handler SM, Sharkey SS, Hudak S, Ouslander JG. Incorporating INTERACT II Clinical Decision Support Tools into Nursing Home Health Information Technology. Ann Longterm Care. 2011;19(11):23-6. | Wrong study design |
| 20 | Harriss LR, Thompson F, Lawson K, O'Loughlin M, McDermott R. Preventable hospitalisations in regional Queensland: potential for primary health? Australian Health Review. 2019;43(4):371-81. | No assessment tool reported.  Wrong study population. |
| 21 | Jackson AH, Fireman E, Feigenbaum P, Neuwirth E, Kipnis P, Bellows J. Manual and automated methods for identifying potentially preventable readmissions: a comparison in a large healthcare system. BMC Med Inform Decis Mak. 2014;14:28. | No assessment tool reported. |
| 22 | Jiménez-Puente A, García-Alegría J, Gómez-Aracena J, Hidalgo-Rojas L, Lorenzo-Nogueiras L, Fernández-Crehuet-Navajas J. [Analysis of the causes and potential avoidability of readmissions in an acute patients' hospital]. Med Clin (Barc). 2002;118(13):500-5. | Wrong study population. |
| 23 | Johnson PC, Xiao Y, Wong RL, D'Arpino S, Moran SMC, Lage DE, et al. Potentially Avoidable Hospital Readmissions in Patients With Advanced Cancer. J Oncol Pract. 2019;15(5):e420-e7. | No assessment tool reported. |
| 24 | Keawpugdee J, Silpasuwan P, Viwatwongkasem C, Boonyamalik P, Amnatsatsue K. Hospital Readmission Risks Screening for Older Adult with Stroke: Tools Development and Validation of a Prediction. Inquiry. 2021;58:469580211018285. | Wrong study population.. |
| 25 | Knighton A, Martin G, Sounderajah V, Warren L, Markiewicz O, Riga C, et al. Avoidable 30-day readmissions in patients undergoing vascular surgery. BJS Open. 2019;3(6):759-66. | No assessment tool reported. |
| 26 | Knox S, Downer B, Haas A, Middleton A, Ottenbacher KJ. Dementia Severity Associated With Increased Risk of Potentially Preventable Readmissions During Home Health Care. J Am Med Dir Assoc. 2020;21(4):519-24.e3. | No assessment tool reported. |
| 27 | Krolak-Salmon P, Roubaud C, Finne-Soveri H, Riolacci-Dhoyen N, Richard G, Rouch I, et al. Evaluation of a mobile team dedicated to behavioural disorders as recommended by the Alzheimer Cooperative Valuation in Europe joint action: observational cohort study. Eur J Neurol. 2016;23(5):979-88. | No assessment tool reported. |
| 28 | Lagoe RJ, Nanno DS, Luziani ME. Quantitative tools for addressing hospital readmissions. BMC Res Notes. 2012;5:620. | No assessment tool reported. |
| 29 | Latus J, Schwab M, Tacconelli E, Pieper FM, Wegener D, Rettenmaier B, et al. Acute kidney injury and tools for risk-stratification in 456 patients with hantavirus-induced nephropathia epidemica. Nephrol Dial Transplant. 2015;30(2):245-51. | Wrong study population. |
| 30 | Leendertse AJ, Van Den Bemt PM, Poolman JB, Stoker LJ, Egberts AC, Postma MJ. Preventable hospital admissions related to medication (HARM): cost analysis of the HARM study. Value Health. 2011;14(1):34-40. | No assessment tool reported. |
| 31 | Liang JW, Cifrese L, Ostojic LV, Shah SO, Dhamoon MS. Preventable Readmissions and Predictors of Readmission After Subarachnoid Hemorrhage. Neurocritical Care. 2018;29(3):336-43. | No assessment tool reported. |
| 32 | Lledó R, Martín E, Jiménez C, Roca R, Gil A, Godoy E, et al. Characteristics of elderly inpatients at high risk of needing supportive social and health care services. Eur J Epidemiol. 1997;13(8):903-7. | No assessment tool reported. |
| 33 | Lohman MC, Scherer EA, Whiteman KL, Greenberg RL, Bruce ML. Factors Associated With Accelerated Hospitalization and Re-hospitalization Among Medicare Home Health Patients. Journals of Gerontology Series A: Biological Sciences & Medical Sciences. 2018;73(9):1280-6. | No assessment tool reported. |
| 34 | Martin C, Hinkley N, Stockman K, Campbell D. Capitated Telehealth Coaching Hospital Readmission Service in Australia: Pragmatic Controlled Evaluation. J Med Internet Res. 2020;22(12):e18046. | No assessment tool reported. |
| 35 | Martin C, Hinkley N, Stockman K, Campbell D. Potentially preventable hospitalizations—The 'pre‐hospital syndrome': Retrospective observations from the MonashWatch self‐reported health journey study in Victoria, Australia. Journal of Evaluation in Clinical Practice. 2021;27(2):228-35. | No assessment tool reported. |
| 36 | Maust DT, Kim HM, Chiang C, Langa KM, Kales HC. Predicting Risk of Potentially Preventable Hospitalization in Older Adults with Dementia. J Am Geriatr Soc. 2019;67(10):2077-84. | No assessment tool reported. |
| 37 | McAna JF, Crawford AG, Novinger BW, Sidorov J, Din FM, Maio V, et al. A predictive model of hospitalization risk among disabled medicaid enrollees. Am J Manag Care. 2013;19(5):e166-74. | No assessment tool reported.  Wrong study population. |
| 38 | McAuliffe LH, Zullo AR, Dapaah-Afriyie R, Berard-Collins C. Development and validation of a transitions-of-care pharmacist tool to predict potentially avoidable 30-day readmissions. Am J Health Syst Pharm. 2018;75(3):111-9. | Wrong study population. |
| 39 | Mi R, Hollander MM, Jones CMC, DuGoff EH, Caprio TV, Cushman JT, et al. A randomized controlled trial testing the effectiveness of a paramedic-delivered care transitions intervention to reduce emergency department revisits. BMC Geriatr. 2018;18(1):104. | No assessment tool reported. |
| 40 | Mihaljevic SE, Howard VM. Incorporating Interprofessional Evidenced-Based Sepsis Simulation Education for Certified Nursing Assistants (CNAs) and Licensed Care Providers Within Long-term Care Settings for Process and Quality Improvement. Crit Care Nurs Q. 2016;39(1):24-33. | No assessment tool reported.  Wrong study design. |
| 41 | Morris JN, Howard EP, Steel K, Schreiber R, Fries BE, Lipsitz LA, et al. Predicting risk of hospital and emergency department use for home care elderly persons through a secondary analysis of cross-national data. BMC Health Serv Res. 2014;14:519. | No assessment tool reported. |
| 42 | Mulder BJ, Tzeng HM, Vecchioni ND. Preventing avoidable rehospitalizations by understanding the characteristics of "frequent fliers". J Nurs Care Qual. 2012;27(1):77-82. | No assessment tool reported. |
| 43 | Nct. OPtimising thERapy to Prevent Avoidable Hospital Admissions in the Multimorbid Older People. <https://clinicaltrialsgov/show/NCT02986425>. 2016. | Study registration record. |
| 44 | Olson CH, Dierich M, Westra BL. Automation of a high risk medication regime algorithm in a home health care population. J Biomed Inform. 2014;51:60-71. | No assessment tool reported. |
| 45 | O'Malley AS, Reschovsky JD, Saiontz-Martinez C. Interspecialty communication supported by health information technology associated with lower hospitalization rates for ambulatory care-sensitive conditions. J Am Board Fam Med. 2015;28(3):404-17. | No assessment tool reported. |
| 46 | O'Riordan Y, Bernard P, Maloney P, Enright A, McGrath C. Safer transitioning Optimising Frail Elderly Patients Care From Hospital to Home. International Journal of Integrated Care (IJIC). 2017;17:1-2. | Poster presentation. |
| 47 | Ouslander JG, Handler SM. Consensus-Derived Interventions to Reduce Acute Care Transfer (INTERACT)-Compatible Order Sets for Common Conditions Associated with Potentially Avoidable Hospitalizations. J Am Med Dir Assoc. 2015;16(6):524-6. | Wrong study design. |
| 48 | Palacholla RS, Fischer NC, Agboola S, Nikolova-Simons M, Odametey S, Golas SB, et al. Evaluating the Impact of a Web-Based Risk Assessment System (CareSage) and Tailored Interventions on Health Care Utilization: Protocol for a Randomized Controlled Trial. JMIR Res Protoc. 2018;7(5):e10045. | Study protocol. |
| 49 | Passey ME, Longman JM, Johnston JJ, Jorm L, Ewald D, Morgan GG, et al. Diagnosing Potentially Preventable Hospitalisations (DaPPHne): protocol for a mixed-methods data-linkage study. BMJ Open. 2015;5(11):e009879. | Study protocol. |
| 50 | Patel KK, Vakharia N, Pile J, Howell EH, Rothberg MB. Preventable Admissions on a General Medicine Service: Prevalence, Causes and Comparison with AHRQ Prevention Quality Indicators-A Cross-Sectional Analysis. J Gen Intern Med. 2016;31(6):597-601. | No assessment tool reported. |
| 51 | Pérez-Rubio A, Santos S, Luquero FJ, Tamames S, Cantón B, Castrodeza JJ. [Evaluation of the appropriateness of stays in a third level hospital]. An Sist Sanit Navar. 2007;30(1):29-36. | Wrong study population. |
| 52 | Peris A, Zagli G, Maccarrone N, Batacchi S, Cammelli R, Cecchi A, et al. The use of Modified Early Warning Score may help anesthesists in postoperative level of care selection in emergency abdominal surgery. Minerva Anestesiol. 2012;78(9):1034-8. | Wrong study population. |
| 53 | Pileggi C, Bianco A, Di Stasio SM, Angelillo IF. Inappropriate hospital use by patients needing urgent medical attention in Italy. Public Health. 2004;118(4):284-91. | Wrong study population. |
| 54 | Porath A, Schlaeffer F, Lieberman D, Porath A, Schlaeffer F, Lieberman D. Appropriateness of hospitalization of patients with community-acquired pneumonia. Annals of Emergency Medicine. 1996;27(2):176-83. | Wrong study population. |
| 55 | Salzman BE, Knuth RV, Cunningham AT, LaNoue MD. Identifying Older Patients at High Risk for Emergency Department Visits and Hospitalization. Popul Health Manag. 2019;22(5):394-8. | No assessment tool reported. |
| 56 | Sánchez-García S, Juárez-Cedillo T, Mould-Quevedo JF, García-González JJ, Contreras-Hernández I, Espinel-Bermudez MC, et al. The hospital appropriateness evaluation protocol in elderly patients: a technique to evaluate admission and hospital stay. Scand J Caring Sci. 2008;22(2):306-13. | Wrong study population. |
| 57 | Sarmento J, Alves C, Oliveira P, Sebastião R, Santana R. [Characterization and Evolution of Avoidable Admissions in Portugal: The Impact of Two Methodologic Approaches]. Acta Med Port. 2015;28(5):590-600. | Wrong study population. |
| 58 | Selker HP, Beshansky JR, Griffith JL, Aufderheide TP, Ballin DS, Bernard SA, et al. Use of the acute cardiac ischemia time-insensitive predictive instrument (ACI-TIPI) to assist with triage of patients with chest pain or other symptoms suggestive of acute cardiac ischemia. A multicenter, controlled clinical trial. Annals of Internal Medicine. 1998;129(11):845-55. | Excluded here because already included in Category 1. |
| 59 | Sengupta R, Loftus TM, Doers M, Jandarov RA, Phillips M, Ko J, et al. Resting Borg score as a predictor of safe discharge of chronic obstructive pulmonary disease from the emergency department observation unit. Academic Emergency Medicine. 2020;27(12):1302-11. | Wrong study population. |
| 60 | Shanahan TAG, Fuller GW, Sheldon T, Turton E, Quilty FMA, Marincowitz C. External validation of the Dutch prediction model for prehospital triage of trauma patients in South West region of England, United Kingdom. Injury. 2021;52(5):1108-16. | No assessment tool reported.  Wrong study population. |
| 61 | Stiell IG, Perry JJ, Clement C, Brison RJ, Rowe BH, Aaron S, et al. Creation of the Canadian heart failure risk scale for acute heart failure patients. Academic emergency medicine Conference: 2017 annual meeting of the society for academic emergency medicine, SAEM 2017 United states. 2017;24:S23. | Poster presentation. |
| 62 | Stober MJ, Hager K, Rinker G. Assessment and Management Tools for Advancing Disease. Home Healthc Now. 2022;40(3):159-66. | No full text. |
| 63 | Sutherland T, David-Kasdan JA, Beloff J, Mueller A, Whang EE, Bleday R, et al. Patient and Provider-Identified Factors Contributing to Surgical Readmission After Colorectal Surgery. J Invest Surg. 2016;29(4):195-201. | No assessment tool reported.  Wrong study population. |
| 64 | Tinetti ME, Charpentier P, Gottschalk M, Baker DI. Effect of a restorative model of posthospital home care on hospital readmissions. J Am Geriatr Soc. 2012;60(8):1521-6. | No assessment tool reported. |
| 65 | Tuso P, Watson HL, Garofalo-Wright L, Lindsay G, Jackson A, Taitano M, et al. Complex case conferences associated with reduced hospital admissions for high-risk patients with multiple comorbidities. Perm J. 2014;18(1):38-42. | No assessment tool reported. |
| 66 | Ukert B, David G, Smith‐McLallen A, Chawla R, Smith-McLallen A. Do payor-based outreach programs reduce medical cost and utilization? Health Economics. 2020;29(6):671-82. | No assessment tool reported. |
| 67 | van der Does AMB, Kneepkens EL, Uitvlugt EB, Jansen SL, Schilder L, Tokmaji G, et al. Preventability of unplanned readmissions within 30 days of discharge. A cross-sectional, single-center study. PLoS One. 2020;15(4):e0229940. | No assessment tool reported. |
| 68 | Victor CR, Khakoo AA. Is hospital the right place? A survey of 'inappropriate' admissions to an inner London NHS trust. J Public Health Med. 1994;16(3):286-90. | Wrong study population. |
| 69 | Vigod SN, Kurdyak PA, Seitz D, Herrmann N, Fung K, Lin E, et al. READMIT: a clinical risk index to predict 30-day readmission after discharge from acute psychiatric units. J Psychiatr Res. 2015;61:205-13. | No assessment tool reported.  Wrong study population. |
| 70 | Vogelsmeier A, Popejoy L, Kist S, Shumate S, Pritchett A, Mueller J, et al. Reducing Avoidable Hospitalizations for Nursing Home Residents: Role of the Missouri Quality Initiative Intervention Support Team. J Nurs Care Qual. 2020;35(1):1-5. | No assessment tool reported. |
| 71 | Weinberg DS, Kraay MJ, Fitzgerald SJ, Sidagam V, Wera GD. Are Readmissions After THA Preventable? Clin Orthop Relat Res. 2017;475(5):1414-23. | No assessment tool reported. |
| 72 | Weiss M, Yakusheva O, Bobay K. Nurse and patient perceptions of discharge readiness in relation to postdischarge utilization. Med Care. 2010;48(5):482-6. | No assessment tool reported. |
| 73 | Weissman GE, Kerlin MP, Yuan Y, Kohn R, Anesi GL, Groeneveld PW, et al. Potentially Preventable Intensive Care Unit Admissions in the United States, 2006-2015. Ann Am Thorac Soc. 2020;17(1):81-8. | No assessment tool reported. |
| 74 | Zhang Y, Zhang Y, Sholle E, Abedian S, Sharko M, Turchioe MR, et al. Assessing the impact of social determinants of health on predictive models for potentially avoidable 30-day readmission or death. PLoS One. 2020;15(6):e0235064. | Wrong study population. |
| 75 | Zografakis‐Sfakianakis M, De Bree E, Linardakis M, Messaritaki A, Askitopoulou H, Papaioannou A, et al. The value of the Modified Early Warning Score for unplanned Intensive Care Unit admissions of patients treated in hospital general wards. International Journal of Nursing Practice (John Wiley & Sons, Inc). 2018;24(3):1-. | Wrong study population. |
