# Supplementary material for: Assessment tools addressing avoidable care transitions in older adults: a systematic literature review
Source: Eur Geriatr Med. 2024 Nov 29;15(6):1587–601. doi: 10.1007/s41999-024-01106-7 (PMC11632047; doi:10.1007/s41999-024-01106-7)
Supplement: Supplementary file 7 — Supplementary file7 (DOCX 12 KB) [file 41999_2024_1106_MOESM7_ESM.docx]

**Supplementary file 7: Final search string**

**(("tool" OR "tools" OR "toolkit" OR "toolkits" OR intervention* OR "instrument" OR "instruments" OR guideline OR "guidelines") AND (("avoidable" AND ("transition*" OR "transitions" OR "transfer" OR "transfers" OR "hospitalization" OR "hospitalizations" OR "admission" OR "admissions" OR "readmission" OR "readmissions")) OR "inappropriate transfer" OR "inappropriate transfers" OR "inappropriate hospitalization" OR "inappropriate hospitalizations" OR "inappropriate admission" OR "inappropriate admissions" OR "burdensome transition*" OR "burdensome transitions" OR "preventable hospitalization" OR "preventable hospitalizations" OR "preventable admission" OR "preventable admissions" OR "preventable readmission" OR "preventable readmissions" OR "inadequate transition*" OR "inadequate transfer" OR "inadequate admission" OR "inadequate admissions" OR "unnecessary transition*" OR "unnecessary transitions" OR "unnecessary transfer" OR "unnecessary transfers" OR "unnecessary hospitalization" OR "unnecessary hospitalizations" OR "unnecessary admission" OR "unnecessary admissions" OR "unnecessary readmission" OR "unnecessary readmissions" OR "ineffective transition*" OR "ineffective transitions" OR "ineffective transfer" OR "inefficient transition*" OR "inefficient transfer" OR "inefficient transfers")) AND ("senior" OR "seniors" OR "older adults" OR "elderly" OR "elderlies" OR "aged")**

**PubMed: No human and time restriction 721 results, 23.06.2022**

**CINAHL: No expanders (no equivalent subjects): 349 results, 23.06.2022**

**CENTRAL: No search word variations, 196 results, 23.06.2022**
